# Synthesis and Evaluation of Aromatic A-Ring 23-Oxavitamin D_3_ Analogues as Hedgehog Pathway Inhibitors

**DOI:** 10.3390/ijms26041631

**Published:** 2025-02-14

**Authors:** Wang Chen, Feifan Lai, Jianghe Xu

**Affiliations:** 1 School of Biological Science and Engineering, Shaanxi University of Technology, Hanzhong 723000, China; 18791050493@163.com (F.L.); 18717419777@163.com (J.X.); 2 Shaanxi Engineering Research Center of Natural Active Products Industrialization, Hanzhong 723000, China

**Keywords:** 23-oxavitamin D_3_ analogues, Hedgehog pathway, smoothened, inhibitors

## Abstract

The Hedgehog (Hh) signaling pathway plays a crucial role in the initiation and progression of tumors, and Hh inhibitors have been used as potential chemotherapeutic agents for the treatment of basal cell carcinomas (BCCs). Vitamin D_3_ (VD_3_) and its derivatives have been identified as potent Hh inhibitors. However, the selectivity of VD_3_ derivatives to vitamin D receptor (VDR) and the Hh signaling pathway still needs optimization. In this study, a series of aromatic A-ring mimics VD_3_ analogues that contain a C-23 oxygen atom or incorporate C-25 hydroxyl on side chains were designed and synthesized. These compounds were tested in various cell lines for anti-Hh activity, with analogues **3j** and **4i** identified as potent inhibitors. Mechanism studies showed their anti-Hh effects are mainly due to targeting Smoothened (Smo) without binding to the cyclopamine site. Structure-activity relationship (SAR) studies revealed that VD_3_-based inhibitors enhance anti-Hh activity by adding a hydroxyl group at C25 while reducing VDR activity by incorporating an oxygen atom into the side chain.

## 1. Introduction

The Hedgehog (Hh) signaling pathway is essential for directing normal growth and tissue patterning during embryonic development. In adults, the Hh signaling pathway plays an important role in tissue repair after injury, maintaining homeostasis in various tissues and organs, and tumorigenesis [1]. The pathway consists of two transmembrane receptor proteins, Patched (Ptch) and Smoothened (Smo), along with several cytosolic proteins, such as fusion suppressor factor (SuFu) and the Gli family of transcription factors associated with gliomas [2]. In the absence of Hh ligands, Ptch1 binds to Smo, inhibiting its activity and negatively regulating the Hh signaling pathway. When Hh ligands bind to Ptch1, Ptch1 dissociates from Smo, thereby relieving the inhibition, allowing Smo to activate the Hh signaling pathway through PKA-dependent cleavage of Gli proteins [3]. Consequently, Ptch is regarded as a tumor suppressor, whereas the constitutively active form of Smo is oncogenic and can operate independently of ligand binding to Ptch. The constitutive activation of the Hh signaling pathway caused by activating mutations of Smo and inactivating mutations of Ptch1 or the SuFu can lead to basal cell carcinomas (BCCs) and medulloblastoma (MB) [4]. Due to their role in cancer development, Hh inhibitors have been used as potential chemotherapeutic agents for the treatment of Hh-driven tumors.

**Figure 1:**
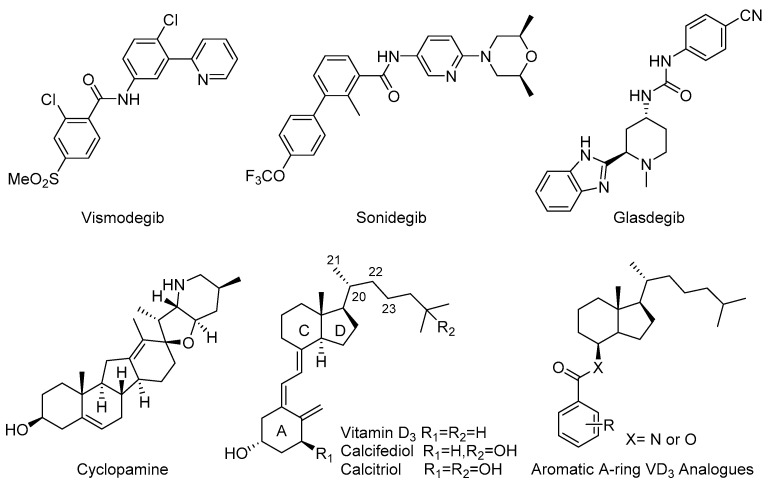
Hh signaling pathway inhibitors.

Over the past decade, several categories of Hh inhibitors (Figure 1) have been developed that block the Hh signaling pathway at different steps, including Hh-directed acyltransferase inhibitors, small molecule Hh inhibits, Smo inhibitors, and Gli inhibitors [5]. Multiple Hh inhibitors are under preclinical and clinical development, with Smo inhibitors being the most promising in terms of druggability. Vismodegib [6] and Sonidegib [7] were approved by the FDA in 2012 and 2015, respectively, for the treatment of locally advanced BCCs, and Vismodegib was also approved for metastatic BCCs. Glasdegib was approved for the treatment of newly diagnosed AML in combination with low-dose cytarabine in 2018 [8]. In addition to the drugs in clinics, BMS-833923, Taladegib, Saridegib (analogue of the first Hh inhibitor cyclopamine), TAK-441, and Itraconazole are also Smo inhibitors currently undergoing various clinical trials and have demonstrated promising clinical responses [9]. However, the limited efficacy against other types of cancers, along with the development of acquired resistance and relapse of Vismodegib and Sonidegib due to mutations in the amino acid of Smo binding site [10], has spurred ongoing efforts to discover alternative Smo inhibitors.

Vitamin D_3_ (VD_3_), a metabolic precursor of calcifediol and calcitriol, was identified as a Hh inhibitor, suggested to exert its effects by directly binding to Smo in a patched-dependent manner [11]. VD_3_ and calcitriol down-regulate the Hh signaling pathway in Hh-dependent cell culture, as well as in murine models of BCCs [12,13]. To evaluate the potential antitumor effects of VD_3_, relevant clinical trials were conducted on patients with BCCs, pancreatic cancer, acute myeloid leukemia (AML), non-Hodgkin lymphoma, and lymphoid tissue lymphoma [14]. However, VD_3_ concurrently activates the canonical vitamin D receptor (VDR) signaling, resulting in detrimental side effects. In previous studies, to minimize adverse effects on the VDR system while enhancing Hh inhibition activity, a series of aromatic A-ring VD_3_ analogues were designed, synthesized, and evaluated as Hh inhibitors [15,16,17,18,19,20]. Initial structure-activity relationship (SAR) studies of VD_3_ scaffold demonstrated the natural CD ring is the essential structure for inhibiting Hh signaling pathway, while substituting the A ring can reduce VDR agonism and enhance selectivity [15,21]. Incorporating an oxygen atom into the side chain of VD_3_ can significantly reduce its affinity for VDR and calcemic activity [22,23]. Herein, we report the design, synthesis, and evaluation of aromatic A-ring 23-oxa-VD_3_ analogues as potent selective Hh inhibitors.

## 2. Results and Discussion

### 2.1. Chemistry

Previous reports demonstrated that both calcifediol and calcitriol are more potent Hh inhibitors compared to VD_3_. Notable observations from SAR of VD_3_ showed that increasing the rigidity of the A ring and introducing heteroatoms into the side chain would reduce the affinity for VDR [22]. Therefore, a series of ester-linked aromatic A-ring VD_3_ analogues that incorporated 23-oxa and C25 hydroxyl in the side chain were synthesized and evaluated to determine the anti-Hh activity and selectivity between VDR and the Hh signaling pathway.

**Scheme 1:**
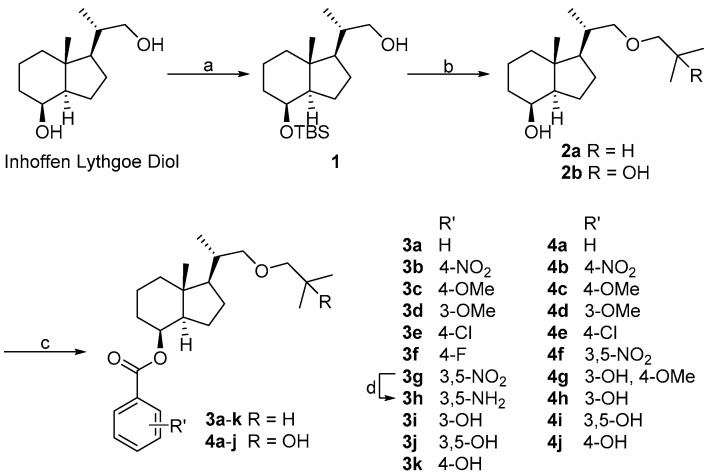
Synthetic route of aromatic A-ring 23-oxavitamin D_3_ analogues. Reagents and conditions: (**a**) i: TBSCl, Imidazole, DMF, 60 °C, 48 h; ii: HCl, THF, r.t., 1 h, yield 95%; (**b**) i: NaH, THF, isobutylbromide, reflux, 48 h for **2a**; *t*BuOK, 18-Crown-6-ether, isobutylene oxide, benzene, reflux, 2 h for **2b**; ii: HF (40%, aq), CH_3_CN, CH_2_Cl_2_, r.t., 6 h, yield 63.1% for **2a** and 78.4% for **2b** from **1**; (**c**) DMAP, EDC∙HCl, Benzoic acid derivatives (benzyl protected derivatives for **3i**–**k** and **4g**–**j**), CH_2_Cl_2_, r.t., 12 h, yields 66.9~91.5% for **3a**–**g** and **4a**–**f**; for **3h**–**k** and **4g**–**j** then condition d; (**d**) 5% Pd/C, H_2_, EtOH, r.t., 12 h, yields 53.4~66.8% for two steps.

In this study, 21 aromatic A-ring 23-oxa-VD_3_ analogues (**3a**–**3k** and **4a**–**4j**) were designed and synthesized (Scheme 1). Commercially available Inhoffen-Lythgoe diol was treated with TBSCl, and subsequent selective deprotection of the primary silyl ether afforded alcohol **1** in 95% yield. Reaction of alcohol **1** with isobutylbromide and NaH followed by deprotection of intermediate gave alcohol **2a** in 63.1% overall yield. Introduction of the C-25 hydroxyl group was performed by reaction of **1** with isobutylene oxide under alkaline condition, resulting in a yield of 78.4% for compound **2b**. Finally, coupling of **2a** or **2b** to the requisite benzoic acids using standard conditions provides the final analogues. For **3i**–**k** and **4g**–**j**, phenolic hydroxyl group protected benzoic acids were used, respectively, followed by deprotection benzyl under H_2_/Pd condition.

### 2.2. Bioactivity

#### 2.2.1. Hh Signaling Pathway and Activity Evaluation

The VD_3_ analogues were initially assessed for their ability to inhibit the Hh signaling pathway by evaluating the effects on endogenous Gli1 mRNA levels at a single concentration (5 μM) co-administration of oxysterols (OHCs; 20(*S*)-hydroxycholesterol, 22(*S*)-hydroxycholesterol at 5 μM each) in C3H10T1/2 cells [20], an Hh-dependent mouse embryonic fibroblast (MEF) cell line. Gli1 is a well-characterized target gene of the Hh signaling pathway and is robustly up-regulated in the MEFs by OHCs, which are known as potent agonists of Hh [24]. VD_3_ derivatives may retain some VDR agonistic activity, thereby compromising the selectivity of these compounds as Hh inhibitors. The selectivity profile of the analogues for Hh inhibition, compared to typical VDR activation, was assessed by measuring the ability to up-regulate Cyp24a1 mRNA levels in the same cellular system and by evaluating their binding to VDR using a fluorescence polarization-based competition assay (Table 1).

The synthesized intermediates **2a** and **2b** exhibit anti-Hh activity comparable to that of VD_3_; however, their activation of VDR signaling is weak, indicating relatively strong selectivity. As with the SAR results with previous aromatic A-ring ester-linked Grundmann’s alcohol analogues [16], masking or substitution of the hydrophilic group on A-ring (**3a**–**g**, **4a**–**f**) resulted in attenuation of anti-Hh activity. Furthermore, C-25 hydroxylation analogues (**4g**–**j**) demonstrate better anti-Hh activity compared to the non-hydroxylation at C-25 (**3h**–**k**). After confirming the anti-Hh activity, the selectivity profile of all the analogues was evaluated by measuring their capacity to bind recombinant VDR by a fluorescence polarization-based competition assay in vitro and up-regulate Cyp24A1 mRNA levels in C3H10T1/2 cells. The complete VD scaffold is the foundation for the compounds binding to VDR, and all these analogues exhibit low affinity for VDR binding (>100 μM) in vitro. Different from literature reports [20], the addition of the C-25 hydroxyl group (**4a**–**j**) to the aromatic A-ring 23-oxa-VD_3_ analogues (**3a**–**k**) had minimal Cyp24A1 up-regulation in C3H10T1/2 cells (10–20 fold). Overall, C-25 hydroxylation analogues demonstrated increases in anti-Hh potency as well as little increase in the up-regulation of Cyp24A1. It appears that incorporating an oxygen atom into the side chain is sufficient to mitigate VDR-mediated effects associated with C-25 hydroxylation.

Following the single concentration studies, the analogues showing potent anti-Hh activity (inhibitory rate > 80% at 5 μM) were selected for concentration-dependent evaluation in C3H10T1/2 and ASZ001 (murine BCC) cells. The ASZ001 cells demonstrate loss of the wild-type Ptch1 allele, overexpression of Gli1, and a cellular morphology characteristic of Hh-dependent BCC tumors [25], which has shown initial promise as an in vitro model of the Hh signaling pathway. Gli1 and Ptch expression were chosen to provide further support that down-regulation of Gli1 is related to Hh inhibition in these cultured cancer cells. The analogues down-regulated Gli1 and Ptch in a concentration-dependent manner in C3H10T1/2 cells (Table 2), and IC_50_ values for down-regulation of Ptch (IC_50_ 1.9–10.3 μM) closely followed those obtained for Gli1 (IC_50_ 1.1–3.8 μM). The results highlight the correlation between Gli1 and Ptch genes in C3H10T1/2 cells, providing strong evidence that A-ring 23-oxavitamin D_3_ analogues reduce Gli1 expression through the inhibition of the Hh signaling pathway. The IC_50_ for down-regulation of Gli1 in ASZ001 for VD_3_ and the analogues (IC_50_ 1.8–6.2 μM) more closely mirrored those obtained in C3H10T1/2 cells. It is reasonable that Gli1 down-regulation in ASZ001 cells correlates well with their reported activities in the Hh-dependent assay in MEFs [15]. The results of the Cyp24A1 mRNA expression analysis in ASZ001 cells were consistent with those in C3H10T1/2 cells, indicating that these analogues have minimal activation of VDR. This is consistent with the above experimental results, in that none of the analogues tested displace the tight binding fluorescent VDR ligand at 100 μM.

#### 2.2.2. In Vitro Anti-Proliferation Activity Evaluation

Analogues that demonstrated potent Hh inhibition in C3H10T1/2 and ASZ001 cells were further evaluated for their anti-proliferative activity in a primary Hh-dependent MB derived from conditional Patched knockout mouse (Ptch-CKO MB) [20]. The GI_50_ value (Table 3) for each of these compounds was significantly higher than the IC_50_ values for Gli1 down-regulation in the C3H10T1/2 and ASZ001 cells. Compounds (**4i**, **4g**, **3j**, **3i**) inhibited proliferation of the primary Ptch-CKO MB cells with GI_50_ value of 2.2–8.1 μM, further highlighting its ability to regulate the canonical Hh signaling pathway. This result was also validated in the ASZ001 cell line.

#### 2.2.3. Mechanism of Inhibiting Hh Signaling Pathway

**Figure 2:**
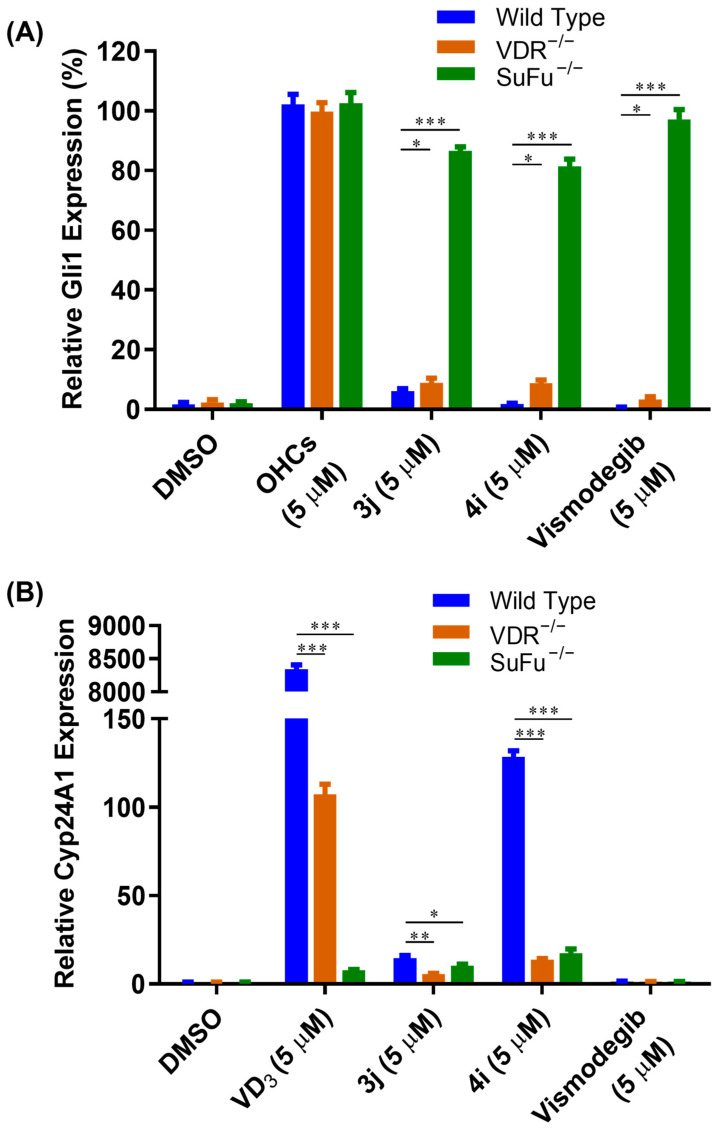
Effects of aromatic A-ring 23-oxavitamin D_3_ analogues on Gli1 down-regulation (**A**) and Cyp24A1 up-regulation (**B**) in C3H10T1/2 cells under 24 h. (*) *p* < 0.05; (**) *p* < 0.01; (***) *p* < 0.001. Gli1 down-regulation of **3j** and **4i** was modestly reduced in VDR^−/−^ cells, whereas it was mostly abolished in SuFu^−/−^ cells. Cyp24A1 up-regulation of **3j** and **4i** was significantly reduced in both VDR^−/−^ and SuFu^−/−^ cells.

Multiple reports have suggested that VD_3_ analogues as Hh inhibitors also exhibit a correlated activation of VDR [16,20]. To further investigate whether the anti-Hh activity for 23-oxavitamin D_3_ analogues is an effect of VDR activation, a series of studies in knockout C3H10T1/2 cells were performed. Compounds **3j** and **4i** were chosen because they exhibit similar Gli1 IC_50_ values across cell lines, despite showing markedly different abilities to up-regulate Cyp24A1 mRNA. The ability of these compounds to regulate Hh and VDR signaling was studied in VDR^−/−^ and SuFu^−/−^ C3H10T1/2 cells. If the compounds regulate Hh signaling independently of VDR, their ability to down-regulate Gli1 mRNA expression should be completely abolished in the SuFu^−/−^ C3H10T1/2 cells [12,20]. The ability of compounds **3j** and **4i** to inhibit Hh signaling was modestly reduced in VDR^−/−^ C3H10T1/2 cells compared to the wild-type cells (Figure 2A). However, the ability of Hh signaling inhibition of compounds **3j** and **4i** was mostly abolished in SuFu^−/−^ C3H10T1/2 cells. Moreover, the well-known Smo antagonist Vismodegib effectively inhibited Hh signaling in the VDR^−/−^ C3H10T1/2 cells but exhibited no activity in the SuFu^−/−^ C3H10T1/2 cells. Furthermore, the ability of compounds **3j** and **4i** to up-regulate Cyp24A1 was significantly reduced in the VDR^−/−^ C3H10T1/2 cells, while similar trends were observed in the SuFu^−/−^ cells (Figure 2B). This may be the effect of Sufu/VDR cross-talk mechanism in MEFs. The result indicated compounds **3j** and **4i** down-regulate Gli1 mainly through the Hh signaling pathway by targeting Smo. These results strongly suggest that the aromatic A-ring 23-oxavitamin D_3_ analogues primarily regulate the canonical Hh signaling pathway.

**Figure 3:**
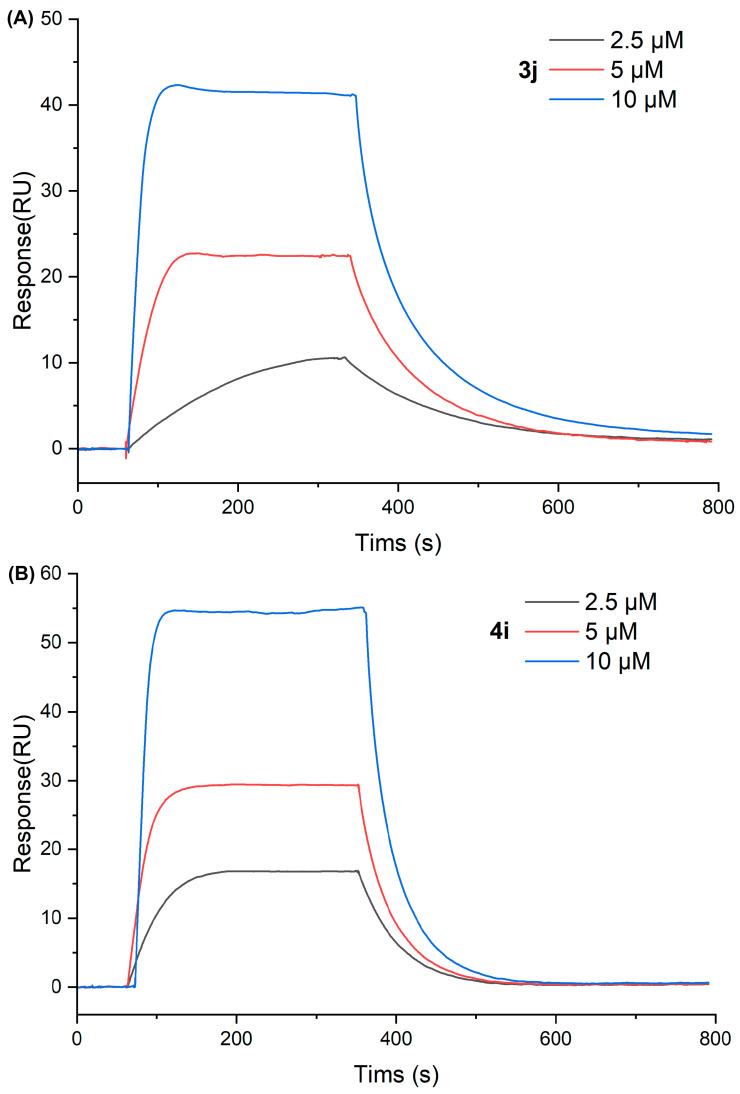
SPR sensorgram for **3j** (**A**) and **4i** (**B**) at concentrations ranging from 2.5 μM to 10 μM for interaction with Smo.

**Figure 4:**
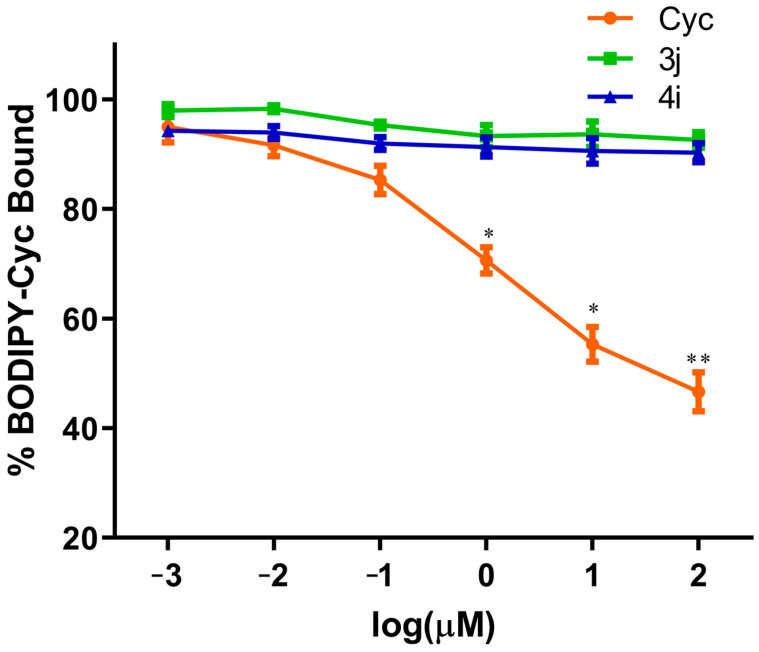
Displacement of BODIPY-Cyc by **3j** and **4i** in HEK293T cells overexpressing Smo. (*) *p* < 0.05; (**) *p* < 0.01 vs. control. Cyc displaced BODIPY-Cyc in a dose-dependent manner, and neither **3j** nor **4i** affected BODIPY-Cyc-Smo binding interactions up to 10 μM.

Surface plasmon resonance (SPR) was used to evaluate the binding capacity of the compounds **3j** and **4i** to Smo. The results showed that compounds **3j** and **4i** exhibited interactions with Smo (Figure 3), indicating that **3j** and **4i** may bind with Smo. To investigate the binding sites of the analogues on Smo, the ability of **3j** and **4i** to displace BODIPY-Cyc from HEK293T cells overexpressing full-length human Smo was evaluated. Cyclopamine displaced BODIPY-Cyc in a concentration-dependent manner, whereas neither **3j** nor **4i** affected BODIPY-Cyc-Smo binding interactions, even at concentrations up to 10 μM (Figure 4). In addition, a combination of **3j** or **4i** with a single concentration of vismodegib (100 nM) enhanced the overall efficacy of **3j** and **4i** but did not change the IC_50_ value of either **3j** or **4i**. Similarly, **3j** or **4i** did not affect the anti-Hh activity of vismodegib. These results suggest that **3j** and **4i** inhibit Hh activity by targeting Smo without binding to the cyclopamine binding site. Smo contains two small molecule binding sites, one located in the extracellular cysteine-rich domain (CRD) and the other in the 7-transmembrane helix (7-TM) region [26]. The 7-TM domain is the binding site for most nonsteroidal Smo inhibitors, such as vismodegib and cyclopamine. Endogenous oxidized sterols can regulate Smo activity by binding CRD [27]. These analogues, as mimetics of steroid hormones, may interact with Smo by binding to CRD.

## 3. Materials and Methods

### 3.1. Chemistry

All chemicals, unless otherwise noted, were acquired from Aladdin (Shanghai, China) and were used as received without further purification. Inhoffen-Lythgoe diol was purchased from Xi’an Qin Kai Biotechnology Co., Ltd. (Xi’an, China). All water employed was ultrapure (>18.2 MΩ·cm^−1^ at 25 °C, Milli-Q, Millipore, Burlington, MA, USA). Solvents were analytical grade, and, when necessary, they were purified and dried by standard methods. ^1^H NMR and ^13^C NMR spectra were obtained on a Brucker Avance-III HD 600 MHz NMR-spectrometer (Billerica, MA, USA) in the indicated solvents. Chemical shifts are expressed in ppm (δ units) relative to TMS signal as internal reference. The high-resolution mass spectrometry (HRMS) was carried out on UPLC-Q-Orbitrap (Thermo Fisher Scientific, Waltham, MA, USA). TLC was performed on Silica Gel GF254, and spots were visualized by iodine vapors or irradiation with UV light (254 nm). Flash chromatography was performed on a column packed with Silica Gel 60 (200–300 mesh). Melting points of compounds were determined by the X4 micro melting point apparatus. The concentration of the reaction solutions involved the use of a rotary evaporator at reduced pressure.

#### 3.1.1. Synthesis of Intermediate **1**

The synthesis of compound **1** is improved from the methods reported in literature [28]. Inhoffen Lythgoe diol (8.48 g, 40 mmol), imidazole (10.88 g, 160 mmol), and TBSCl (18 g, 120 mmol) were dissolved in DMF (100 mL), and the resulting mixture was stirred under 60 °C for 48 h. Then, the solution was cooled to room temperature and quenched with saturated brine (400 mL). The aqueous fraction was extracted with petroleum ether (200 mL × 2). The combined organic layer was washed with water (200 mL × 2) and concentrated. The crude intermediate product was dissolved in THF (300 mL) and treated with concentrated HCl (4 mL). The reaction mixture was stirred at room temperature for 1 h. The reaction was quenched with saturated aqueous NaHCO_3_ (50 mL). The mixture was evaporated to dryness under reduced pressure. The residue was extracted with water (200 mL) and ethyl acetate (100 mL × 2). The combined organic layer was washed with saturated brine (200 mL × 2), dried over anhydrous sodium sulfate, filtered, and concentrated. The residue was purified by flash chromatography using petroleum ether/ethyl acetate (*v:v* = 10:1) as eluent to give **1** (13 g, yield: 95% from Inhoffen Lythgoe diol) as colorless oil.

#### 3.1.2. Synthesis of Intermediate **2a** and **2b**

Compound **1** (6.2 g, 19 mmol) was dissolved in anhydrous THF (150 mL), then NaH (60%, 2.28 g, 57 mmol) was added to the solution. The reaction mixture was stirred at room temperature for 0.5 h, and then 1-bromo-2-methylpropane (8.22 g, 60 mmol) was added. The reaction mixture was brought to reflux and stirred under N_2_ for 48 h. The reaction mixture was cooled to room temperature and quenched with saturated brine (200 mL). The aqueous fraction was extracted with ethyl acetate (100 mL × 2). The combined organic layer was washed with water (100 mL × 2) and concentrated. The crude intermediate product was dissolved in acetonitrile/CH_2_Cl_2_ (*v:v* = 7:3, 100 mL) and treated with 40% HF (2.2 mL, 50 mmol). The reaction mixture was stirred at room temperature for 8 h and then quenched with saturated aqueous NaHCO_3_ (50 mL). The mixture was concentrated and extracted with water (100 mL) and ethyl acetate (50 mL × 2). The combined organic layer was washed with saturated brine (100 mL), dried over anhydrous sodium sulfate, filtered, and concentrated. The residue was purified by flash chromatography using petroleum ether/ethyl acetate (*v:v* = 10:1) as eluent to give **2a** (3.2 g, yield: 63.1% from **1**) as colorless oil.

Compound **1** (4.95 g, 15 mmol), 18-Crown-6 (1.95 g, 7.5 mmol), isobutylene oxide (3.6 g, 45 mmol), and *t*BuOK (8.4 g, 75 mmol) were dissolved in anhydrous benzene (100 mL), and the resulting mixture was stirred under reflux for 2 h. The reaction mixture was cooled to room temperature and quenched with saturated brine (100 mL). The aqueous fraction was extracted with ethyl acetate (100 mL × 2). The combined organic layer was washed with water (100 mL × 2) and concentrated. The crude intermediate product was dissolved in acetonitrile/CH_2_Cl_2_ (*v:v* = 7:3, 80 mL) and treated with 40% HF (1.76 mL, 40 mmol). The reaction mixture was stirred at room temperature for 8 h and then quenched with saturated aqueous NaHCO_3_ (40 mL). The mixture was concentrated and extracted with water (100 mL) and ethyl acetate (50 mL × 2). The combined organic layer was washed with saturated brine (100 mL), dried over anhydrous sodium sulfate, filtered, and concentrated. The residue was purified by flash chromatography using petroleum ether/ethyl acetate (*v:v* = 5:1) as eluent to give **2b** (3.3 g, yield: 78.4% from **1**) as colorless oil.

#### 3.1.3. General Procedure for the Synthesis of Target Compounds

The synthesis of **3a**–**k** and **4a**–**j** is depicted in Scheme 1. Compound **2a** or **2b** (1 mmol), EDC∙HCl (230 mg, 1.2 mmol), DMAP (catalytic quantity), and appropriate benzoic acid derivatives (1.2 mmol) were dissolved respectively in anhydrous CH_2_Cl_2_ (15 mL). The resulting mixtures were stirred at room temperature for 12 h until the reaction was complete, which was detected by TLC. Then, the reaction mixtures were treated with water (30 mL) and CH_2_Cl_2_ (20 mL × 2). The combined organic layer was dried over anhydrous sodium sulfate, filtered, and concentrated. Purification by chromatography using petroleum ether/ethyl acetate (*v:v* = 20:1–5:1) yielded the target product. For **3i**–**k** and **4g**–**j**, the phenolic hydroxyl group of appropriate benzoic acid derivatives should be protected by benzyl. The general procedure for removing benzyl protection was catalytic by 5% Pd/C under hydrogen in EtOH.

#### 3.1.4. Characterization Data

Characterization data (NMR data, NMR spectrum, HPLC chromatogram) of all compounds can be found in the Supplementary Materials (including Figures S1–S56).

### 3.2. Bioactivity Evaluation

#### 3.2.1. Cellular Studies

Mouse embryonic fibroblast cell line (C3H10T1/2, wild-type, VDR^−/−^, SuFu^−/−^), mouse keratinocyte cell line (ASZ001), and human embryonic kidney cell line (HEK293T) were purchased from China Center for Type Culture Collection (CCTCC; Wuhan, Hubei, China) and cultured under standard protocols [16]. OHCs, Vismodegib, and VD_3_ were purchased from Sigma-Aldrich, St. Louis, MO, USA. C3H10T1/2 cells are Hh-dependent MEFs and exhibit a good response to both Hh and VDR signaling pathways. Therefore, they can be used to assess the inhibitory strength of the Hh signaling and the selectivity of the VDR signaling. VDR^−/−^ and SuFu^−/−^ C3H10T1/2 cells were used to study compounds selectivity for Hh and VDR signaling. If compounds regulate Hh signaling independently of VDR, their ability to down-regulate Gli1 mRNA expression should be abolished in SuFu^−/−^ C3H10T1/2 cells. ASZ001 cells lack the wild-type Ptch1 allele, overexpress Gli1, and exhibit a morphology typical of Hh-dependent BCC tumors. The loss of Ptch in ASZ001 cells prevents Smo inhibition, so down-regulating Gli1 reflects direct Smo inhibition by the compound. Comparing results from ASZ001 and C3H10T1/2 cells highlights the compound’s inhibition of the Hh signaling pathway.

RNA isolation in C3H10T1/2 cells was performed in 24-well plates (50,000 cells plated; TRIZOL Reagent); in ASZ001, cells were done in 35 mm dishes (300,000 cells plated; TRIZOL Reagent). cDNA synthesis was performed utilizing the SweScript All-in-One RT SuperMix Kit (Servicebio, Wuhan, China) per the manufacturer’s instructions on a BioRad S1000 Thermal Cycler (Hercules, CA, USA). qPCR (Hh signaling pathway and VDR) analysis of target genes was performed on Step OnePlus (ABI) using the Taqman Gene Expression Assays [18,29]: mouse ActB (Mm00607939_s1), mouse GLI1 (Mm00494645_ml), mouse PTCH1(Mm00436026_m1), and mouse CYP24A1 (Mm00487244_ml). Relative gene expression levels were computed via the ΔΔCt method. ActinB mRNA expression levels of all samples were normalized to DMSO control ActinB mRNA levels (vehicle; set at 1.00). DMSO control Gli1 mRNA levels were set to 1.0, and Gli1 mRNA levels for OHC-treated samples (up-regulated) were set to 100% relative to DMSO control. The mRNA expression levels from drug-treated samples were compared to OHCs levels (Gli1, PTCH) and DMSO control (Cyp24A1).

#### 3.2.2. VDR Binding Assay

VDR binding assay was performed using a PolarScreen VDR Competitor Assay Red kit (Thermo Fisher Scientific, Carlsbad, CA, USA) per the manufacturer’s instructions. Compounds were evaluated at 100 μM in 2.5% final DMSO in triplicate. Calcitriol is utilized as a positive control for VDR binding [15].

#### 3.2.3. In Vitro Cell Proliferation Assay

Ptch-CKO MB cells, derived from a conditional Patched knockout mouse, lack Ptch-like ASZ001 cells. Consequently, the inhibitory effect of the compound on Ptch-CKO MB cells directly reflects its suppression of the canonical Hh signaling pathway. Cell viability was assessed by MTS proliferation assay kit (Promega, Madison, WI, USA) according to the manufacturer’s protocol. Culture conditions and experimental protocols to determine GI_50_ values in the primary murine Ptch-CKO MB cells were as previously described [30,31].

#### 3.2.4. Surface Plasmon Resonance (SPR) Analysis

SPR analysis was conducted as described in papers [32,33]. The analyses of interactions between Smo protein (ACRO Biosystems, SMO-H52P3, Newark, DE, USA) and compounds at different concentrations (2.5, 5, 10 μM) was performed in PBS and a flow rate of 2 μL/s at 4 °C. The sensor chip surface was regenerated by injection of 0.5% SDS: Glycine-HCl (pH = 2.0) = 1:1 solution.

#### 3.2.5. Fluorescence Binding Assay

HEK293T cells are highly efficient in transfection and exogenous gene expression, making them ideal for fluorescence labeling experiments. They can easily express exogenous Smo to bind with fluorescently labeled cyc [16]. For flow cytometry experiments [16,32], HEK293T cells were transfected with Smo-Myc3, co-incubated with BODIPY Cyc and different compounds, and then incubated with anti-Myc antibody, followed by Alexa Flour-647 donkey anti-rabbit antibody. Finally, cells were analyzed for green fluorescence using flow cytometry.

#### 3.2.6. Statistical Analysis

Statistical analysis was performed using the GraphPad Prism 8 software. Statistical tests were appropriately chosen for each experiment. For all other experiments, *p*-values were determined using Student’s *t*-test, and statistical significance was set at *p* < 0.05. Results are expressed as mean ± SEM for at least two separate experiments performed in triplicate.

## 4. Conclusions

Currently, the key challenges for VD_3_ and its analogues as Hh inhibitors are enhancing potency and selectivity. Compared to previous aromatic A-ring VD_3_ derivatives, this study primarily targets modifications to the side chain region, specifically the introduction of an oxygen atom at C23 and a hydroxyl group at C25. In the present research, a series of aromatic A-ring 23-oxavitamin D_3_ analogues were designed and synthesized as Hh inhibitors. The preparation of these analogues was performed by linking aromatic functionalities to the Inhoffen Lythgoe diol-derived CD ring section via an ester bond. Single-dose experiment in C3H10T1/2 cells and VDR binding assay showed initial selective anti-Hh activity. Concentration-dependent analysis in Hh-dependent cancer cell lines and knockout cell line comparative studies of several analogues further demonstrated the ability of these analogues to selectively inhibit Hh signaling in vitro. The analogues showing potent anti-Hh activity in C3H10T1/2 cells with an IC_50_ value of 1.1–4.2 μM were further evaluated in Ptch-CKO MB and ASZ001cells to verify their anti-proliferative activity in vitro. The ability of **3j** and **4i** to inhibit Hh was modestly reduced in VDR^−/−^ MEFs and mostly abolished in SuFu^−/−^ MEFs, suggesting that the anti-Hh properties of these compounds are primarily mediated by targeting Smo. Fluorescence binding assay further suggests these compounds did not bind to the cyclopamine binding site.

There is well-established crosstalk between VDR and Hh signaling, and the activation of VDR may disrupt the activation/transcription of Hh target genes [20,34]. However, VDR activation becomes an undesired effect when VD_3_ derivatives function as Hh inhibitors. SAR for this class of compounds demonstrated that innovated hydrophilic residues in the aromatic ring resulted in enhanced anti-Hh activity, and the preferred position of the hydrophilic group was 3-position. The addition of the C-25 hydroxyl group in the analogues resulted in enhanced anti-Hh activity; however, no substantial increase in VDR agonistic activity was observed. As expected, the incorporation of oxygen atom at C23 is sufficient to reduce the VDR activity [23] associated with C-25 hydroxylation. Future VD_3_-based Hh inhibitors could enhance selectively anti-Hh activity by introducing hydroxyl group at C25, while reducing VDR activity by incorporating oxygen atom into the side chain.

In summary, 21 aromatic A-ring 23-oxavitamin D_3_ derivatives were successfully synthesized in this study. These compounds were evaluated in multiple cell lines for their anti-Hh activity, and the analogues **3j** and **4i** were identified as potent Hh inhibitors. These analogues primarily regulate the canonical Hh signaling pathway by binding to the CRD of Smo and represent novel structural modalities of VD_3_-based Hh inhibitors.

## Figures and Tables

**Table 1:** Selective inhibition of the Hh signaling pathway for aromatic A-ring 23-oxavitamin D_3_.

Cpd ^a^	Gli1 mRNA (%) ^b^	Cyp24A1 mRNA ^c^	VDR Binding (µM) ^d^
DMSO	1	1	-
OHCs	100	-	-
VD_3_	32.5 ± 0.6	8346 ± 102	>100
**2a**	36.8 ± 3.6	1.6 ± 0.3	>100
**2b**	42.7 ± 3.4	76.8 ± 6.3	>100
**3a**	56.8 ± 3.5	35.7 ± 2.6	>100
**3b**	74.2 ± 4.1	24.6 ± 2.1	>100
**3c**	89.6 ± 4.3	36.7 ± 2.9	>100
**3d**	102.3 ± 8.4	13.5 ± 1.1	>100
**3e**	23.5 ± 2.7	67.8 ± 4.8	>100
**3f**	45.4 ± 3.1	46.5 ± 3.4	>100
**3g**	32.1 ± 2.4	9.8 ± 1.4	>100
**3h**	5.6 ± 0.9	6.7 ± 0.8	>100
**3i**	6.1 ± 1.1	13.8 ± 1.4	>100
**3j**	2.4 ± 0.4	11.4 ± 1.3	>100
**3k**	7.3 ± 1.9	32.1 ± 3.2	>100
**4a**	43.8 ± 3.6	94.6 ± 12.7	>100
**4b**	54.6 ± 3.8	92.7 ± 15.9	>100
**4c**	76.5 ± 7.5	120.4 ± 18.6	>100
**4d**	62.3 ± 4.8	86.8 ± 11.3	>100
**4e**	26.1 ± 2.1	105.1 ± 21.4	>100
**4f**	30.7 ± 2.5	131.2 ± 19.7	>100
**4g**	2.5 ± 0.5	83.6 ± 15.6	>100
**4h**	3.8 ± 0.4	87.4 ± 13.7	>100
**4i**	1.7 ± 0.3	128.9 ± 16.8	>100
**4j**	4.6 ± 0.8	126.8 ± 20.5	>100

^a^ All analogues evaluated at 5 μM co-administration of OHCs 5 μM under 24 h. ^b^ Relative Gli1mRNA levels compared to OHCs (set to 100%). ^c^ Fold up-regulation of Cyp24A1 mRNA levels relative to DMSO (set to 1.0). ^d^ Calcitriol is utilized as a positive control for VDR binding.

**Table 2:** Concentration-dependent inhibition of the Hh signaling pathway for aromatic A-ring 23-oxavitamin D_3_ analogues in C3H10T1/2 and ASZ001 cells.

Cpd	C3H10T1/2		ASZ001	
	Gli1 ^a^	Ptch ^a^	Gli1 ^a^	Cyp24A1 ^b^
VD_3_	4.2 ± 0.4	3.3 ± 0.4	2.1 ± 0.2	821.4 ± 26.3
**3h**	3.2 ± 0.3	7.8 ± 0.6	4.7 ± 0.4	5.1 ± 0.3
**3i**	2.3 ± 0.7	5.6 ± 0.6	5.2 ± 0.3	2.3 ± 0.2
**3j**	1.8 ± 0.3	3.6 ± 0.5	5.5 ± 0.7	2.1 ± 0.3
**3k**	3.8 ± 0.4	10.3 ± 1.1	6.2 ± 1.1	5.8 ± 0.3
**4g**	2.1 ± 0.2	3.8 ± 0.4	3.7 ± 0.7	56.8 ± 5.1
**4h**	2.8 ± 0.2	5.7 ± 0.5	4.1 ± 0.5	60.3 ± 6.4
**4i**	1.1 ± 0.1	1.9 ± 0.3	1.8 ± 0.3	87.4 ± 10.6

^a^ IC_50_ values (μM) represent the mean ± SEM of at least two separate experiments performed in triplicate. ^b^ Cyp24A1 mRNA levels relative to DMSO (set to 1.0), analogues evaluated at 5 μM under 24 h.

**Table 3:** In vitro anti-proliferation activity in primary Ptch-CKO MB and ASZ001 cells.

Cpd	Ptch-CKO MB (GI_50_, µM)	ASZ001 (GI_50_, µM)
Vismodegib	0.78 ± 0.1	2.1
VD_3_	6.4 ± 0.7	15.6 ± 1.7
**3h**	>10	>20
**3i**	8.1 ± 1.1	>20
**3j**	6.7 ± 0.8	13.4 ± 1.4
**3k**	>10	>20
**4g**	3.4 ± 0.5	7.8 ± 1.1
**4h**	>10	>20
**4i**	2.2 ± 0.3	5.8 ± 0.8
**4j**	>10	>20

All analogues evaluated following 48 h incubation. All values represent the mean ± SEM of at least two separate experiments performed in triplicate.

## Data Availability

The data presented in this study are available on request from the corresponding author.

## Supplementary Materials

The supporting information can be downloaded at: https://www.mdpi.com/article/10.3390/ijms26041631/s1.
