# Supplementary material for: Synthesis and Evaluation of Aromatic A-Ring 23-Oxavitamin D3 Analogues as Hedgehog Pathway Inhibitors
Source: Int J Mol Sci. 2025 Feb 14;26(4):1631. doi: 10.3390/ijms26041631 (PMC11855207; doi:10.3390/ijms26041631)
Supplement: Supplementary file 1 [file ijms-26-01631-s001.zip › ijms-3466581-supplementary.pdf]

Supplementary Materials for

Synthesis and Evaluation of Aromatic A-Ring 23-Oxavitamin D_3 Analogues as Hedgehog Pathway Inhibitors

Wang Chen [1,2,*], Feifan Lai [1,2] and Jianghe Xu [1,2]

[1] School of Biological Science and Engineering, Shaanxi University of Technology, Hanzhong 723000, China; 18791050493@163.com (F.L.); 18717419777@163.com (J.X.)
[2] Shaanxi Engineering Research Center of Natural Active Products Industrialization, Hanzhong 723000, China
* Correspondence: chenwang@snut.edu.cn; Tel.: +86-0916-2641716

Contents

1. Characterization data 1

2. ^{1}H-NMR and ^{13}C NMR spectrum 8

3. HPLC chromatogram 32

1. Characterization data

*1.1. (S)-2-((1R,3aR,4S,7aR)-4-((tert-butyldimethylsilyl)oxy)-7a-methyloctahydro-1H-inden-1-yl)propan-1-ol (**1**).* Yield: 95% for two steps; colorless oil. ESI-MS: *m/z* 327.4 $[M+H]^+$. ^{1}H NMR (600 MHz, $CDCl_3$) δ 4.00 (d, *J* = 2.5 Hz, 1H), 3.63 (dd, *J* = 10.5, 3.2 Hz, 1H), 3.37 (dd, *J* = 10.5, 6.8 Hz, 1H), 1.95 (d, *J* = 12.5 Hz, 1H), 1.79 (ddt, *J* = 16.2, 9.4, 3.4 Hz, 2H), 1.70-1.64 (m, 1H), 1.59-1.51 (m, 2H), 1.37 (ddd, *J* = 12.1, 6.0, 3.6 Hz, 4H), 1.32-1.22 (m, 2H), 1.17-1.10 (m, 2H), 1.02 (d, *J* = 6.6 Hz, 3H), 0.93 (s, 3H), 0.89 (s, 9H), -0.00 (d, *J* = 2.4 Hz, 6H); ^{13}C NMR (150 MHz, $CDCl_3$) δ 69.37, 68.04, 53.15, 52.88, 42.18, 40.61, 38.31, 34.44, 26.83, 26.00, 25.84, 23.13, 18.05, 17.66, 16.69, 13.79, -4.78, -5.13.

*1.2. (1R,3aR,4S,7aR)-1-((S)-1-isobutoxypropan-2-yl)-7a-methyloctahydro-1H-inden-4-ol (**2a**).* Yield: 63.1% for two steps; colorless oil. $[\alpha]_D$=+36.5° (*c*=1.0 g/100mL, 25℃, in MeOH). HRMS: *m/z* 269.4412 $[M+H]^+$. ^{1}H NMR (600 MHz, $CDCl_3$) δ 4.01 (d, *J* = 2.5 Hz, 1H), 3.30 (dd, *J* = 9.1, 3.3 Hz, 1H), 3.13 (dd, *J* = 9.2, 6.5 Hz, 1H), 3.00 (ddd, *J* = 9.2, 7.4, 5.6 Hz, 2H), 1.96-1.89 (m, 1H), 1.82-1.70 (m, 4H), 1.57 (dd, *J* = 8.8, 5.3 Hz, 1H), 1.43-1.34 (m, 3H), 1.32-1.24 (m, 4H), 1.12-1.05 (m, 3H), 0.94 (d, *J* = 6.6 Hz, 3H), 0.88 (s, 3H), 0.83 (d, *J* = 4.0 Hz, 3H), 0.82 (d, *J* = 4.0 Hz, 3H); ^{13}C NMR (150 MHz, $CDCl_3$) δ 77.03, 74.98, 68.29, 52.58, 51.31, 40.92, 39.19, 35.30, 32.57, 27.39, 25.69, 21.60, 18.45, 18.39, 16.39, 16.32, 12.58.

*1.3. (1R,3aR,4S,7aR)-1-((S)-1-(2-hydroxy-2-methylpropoxy)propan-2-yl)-7a-methyloctahydro-1H-inden-4-ol (**2b**).* Yield: 78.4% for two steps; colorless oil. $[\alpha]_D$=+38.3° (*c*=1.0 g/100mL, 25℃, in MeOH). HRMS: *m/z* 285.2435 $[M+H]^+$. ^{1}H NMR (600 MHz, $CDCl_3$) δ 4.08 (s, 1H), 3.42 (dd, *J* = 9.1, 3.4 Hz, 1H), 3.25 (d, *J* = 8.7 Hz, 1H), 3.23-3.18 (m, 1H), 3.16 (d, *J* = 8.7 Hz, 1H), 2.35 (s, 1H), 2.02-1.96 (m, 1H), 1.85 – 1.78 (m, 3H), 1.70-1.65 (m, 1H), 1.60-1.54 (m, 1H), 1.50-1.42 (m, 3H), 1.37-1.32 (m, 2H), 1.29 (d, *J* = 2.5 Hz, 1H), 1.20 (s, 3H), 1.20 (s, 3H), 1.18 (s, 2H), 1.02 (d, *J* = 6.6 Hz, 3H), 0.95 (s, 3H); ^{13}C NMR (150 MHz, $CDCl_3$) δ 79.30, 76.62, 70.33, 69.27, 53.59, 52.38, 41.96, 40.29, 36.27, 33.64, 26.74, 26.15, 26.14, 22.62, 17.43, 17.36, 13.61.

*1.4. (1R,3aR,4S,7aR)-1-((S)-1-isobutoxypropan-2-yl)-7a-methyloctahydro-1H-inden-4-yl benzoate (**3a**).* Yield: 91.5%; white powder; mp: 114-116°C. HRMS: *m/z* 373.2434 $[M+H]^+$. ^{1}H NMR (600 MHz, $CDCl_3$) δ 8.05 (d, *J* = 7.4 Hz, 2H), 7.55 (t, *J* = 7.4 Hz, 1H), 7.45 (t, *J* = 7.7 Hz, 2H), 5.42 (d, *J* = 1.8 Hz, 1H), 3.37 (dd, *J* = 9.1, 3.3 Hz, 1H), 3.19 (dd, *J* = 9.2, 6.5 Hz, 1H), 3.08 (ddd, *J* = 13.5, 9.0, 7.4 Hz, 2H), 2.07 (d, *J* = 12.6 Hz, 1H), 2.00 (d, *J* = 12.9 Hz, 1H), 1.88-1.77 (m, 2H), 1.70-1.63 (m, 1H), 1.56 (ddd, *J* = 14.1, 6.7, 3.5 Hz, 2H), 1.50-1.46 (m, 2H), 1.46-1.43 (m, 1H), 1.34-1.28 (m, 1H), 1.27-1.22 (m, 3H), 1.06 (s, 3H), 1.05 (d, *J* = 7.6 Hz, 3H), 0.90 (d, *J* = 6.5 Hz, 3H), 0.89 (d, *J* = 6.5 Hz, 3H); ^{13}C NMR (150 MHz, $CDCl_3$) δ 165.48, 131.66, 129.93, 128.55,

127.34, 77.12, 74.96, 71.13, 52.47, 50.41, 41.03, 38.81, 35.52, 29.59, 27.43, 25.64, 21.76, 18.44, 18.39, 17.02, 16.39, 12.60.

*1.5. (1R,3aR,4S,7aR)-1-((S)-1-isobutoxypropan-2-yl)-7a-methyloctahydro-1H-inden-4-yl 4-nitrobenzoate (**3b**).* Yield: 88.6%; white powder; mp: 155-158°C. HRMS: *m/z* 418.2536 $[M+H]^+$. ^1H NMR (600 MHz, $CDCl_3$) δ 8.30 (d, J = 8.8 Hz, 2H), 8.20 (d, J = 8.8 Hz, 2H), 5.45 (d, J = 2.0 Hz,, 1H), 3.37 (dd, J = 9.1, 3.3 Hz, 1H), 3.19 (dd, J = 9.2, 6.5 Hz, 1H), 3.09 (ddd, J = 19.5, 9.0, 7.3 Hz, 2H), 2.09 (d, J = 12.9 Hz, 1H), 2.02 (d, J = 13.3 Hz, 1H), 1.88-1.78 (m, 1H), 1.69-1.64 (m, 1H), 1.61-1.53 (m, 4H), 1.46-1.40 (m, 1H), 1.37-1.31 (m, 1H), 1.29 (dd, J = 13.3, 3.2 Hz, 1H), 1.27-1.22 (m, 2H), 1.06 (d, J = 6.6 Hz, 3H), 1.04 (s, 3H), 0.90 (d, J = 6.3 Hz, 3H), 0.89 (d, J = 6.4 Hz, 3H); ^{13}C NMR (150 MHz, $CDCl_3$) δ 164.60, 150.47, 136.30, 130.63, 123.60, 78.17, 75.93, 73.58, 53.42, 51.29, 42.05, 39.64, 36.52, 30.49, 28.46, 26.57, 22.79, 19.46, 19.41, 17.97, 17.43, 13.64.

*1.6. (1R,3aR,4S,7aR)-1-((S)-1-isobutoxypropan-2-yl)-7a-methyloctahydro-1H-inden-4-yl 4-methoxybenzoate (**3c**).* Yield: 90.3%; white powder; mp: 146-148°C. HRMS: *m/z* 403.2847 $[M+H]^+$. ^1H NMR (600 MHz, $CDCl_3$) δ 7.93 (d, J = 8.9 Hz, 2H), 6.86 (d, J = 8.9 Hz, 2H), 5.31 (d, J = 1.8 Hz,, 1H), 3.79 (s, 3H), 3.30 (dd, J = 9.0, 3.3 Hz, 1H), 3.12 (dd, J = 9.2, 6.5 Hz, 1H), 3.04-2.98 (m, 2H), 2.00 (d, J = 12.7 Hz, 1H), 1.92 (d, J = 15.3 Hz, 1H), 1.78 (dt, J = 13.4, 6.7 Hz, 1H), 1.76-1.68 (m, 2H), 1.62-1.56 (m, 1H), 1.49 (dd, J = 6.9, 2.4 Hz, 1H), 1.42-1.36 (m, 1H), 1.27-1.21 (m, 2H), 1.18-1.14 (m, 3H), 0.98 (d, J = 4.2 Hz, 3H), 0.98 (s, 3H), 0.83 (d, J = 6.6 Hz, 3H), 0.82 (d, J = 6.6 Hz, 3H);^{13}C NMR (150 MHz, $CDCl_3$) δ 165.25, 162.18, 130.53, 122.39, 112.61, 77.12, 74.98, 70.71, 54.41, 52.49, 50.44, 41.04, 38.85, 35.54, 29.64, 27.43, 25.66, 21.77, 18.45, 18.39, 17.05, 16.39, 12.61.

*1.7. (1R,3aR,4S,7aR)-1-((S)-1-isobutoxypropan-2-yl)-7a-methyloctahydro-1H-inden-4-yl 3-methoxybenzoate (**3d**).* Yield: 87.2%; white powder; mp: 141-144°C. HRMS: *m/z* 403.2847 $[M+H]^+$. ^1H NMR (600 MHz, $CDCl_3$) δ 7.58 (d, J = 7.7 Hz, 1H), 7.53 – 7.50 (m, 1H), 7.28 (t, J = 7.9 Hz, 1H), 7.03 (dd, J = 8.1, 2.2 Hz, 1H), 5.34 (d, J = 2.0 Hz, 1H), 3.78 (s, 3H), 3.30 (dd, J = 9.1, 3.3 Hz, 1H), 3.12 (dd, J = 9.2, 6.5 Hz, 1H), 3.06 – 2.96 (m, 2H), 2.00 (d, J = 12.6 Hz, 1H), 1.93 (d, J = 15.2 Hz, 1H), 1.79 (dd, J = 13.3, 6.7 Hz, 1H), 1.76-1.71 (m, 2H), 1.62-1.56 (m, 1H), 1.51 (dd, J = 6.9, 2.6 Hz, 1H), 1.50-1.43 (m, 3H), 1.43-1.38 (m, 1H), 1.29-1.23 (m, 1H), 1.20 (dd, J = 13.5, 3.8 Hz, 1H), 1.18-1.12 (m, 2H), 0.99 (s, 3H), 0.98 (d, J = 6.6 Hz, 3H), 0.83 (d, J = 6.2 Hz, 3H), 0.82 (d, J = 6.3 Hz, 3H); ^{13}C NMR (150 MHz, $CDCl_3$) δ 165.32, 158.56, 131.24, 128.37, 120.94, 118.24, 113.03, 77.11, 74.96, 71.23, 54.36, 52.47, 50.40, 41.04, 38.80, 35.52, 29.57, 27.43, 25.64, 21.76, 18.44, 18.39, 17.02, 16.39, 12.59.

*1.8. (1R,3aR,4S,7aR)-1-((S)-1-isobutoxypropan-2-yl)-7a-methyloctahydro-1H-inden-4-yl 4-chlorobenzoate (**3e**).* Yield: 85.3%; white powder; mp: 153-156°C. ESI-MS: *m/z* 407.2353

[M+H]$^+$. ^{1}H NMR (600 MHz, $CDCl_3$) δ 7.90 (d, J = 8.6 Hz, 2H), 7.35 (d, J = 8.6 Hz, 2H), 5.33 (d, J = 2.0 Hz, 1H), 3.30 (dd, J = 9.1, 3.3 Hz, 1H), 3.12 (dd, J = 9.2, 6.5 Hz, 1H), 3.02 (ddd, J = 16.0, 9.0, 7.3 Hz, 2H), 2.00 (d, J = 12.6 Hz, 1H), 1.92 (d, J = 15.2 Hz, 1H), 1.82-1.75 (m, 1H), 1.75-1.68 (m, 2H), 1.62-1.56 (m, 1H), 1.51-1.44 (m, 3H), 1.39-1.34 (m, 1H), 1.28-1.21 (m, 2H), 1.18-1.12 (m, 2H), 0.98 (d, J = 6.6 Hz, 3H), 0.96 (s, 3H), 0.83 (d, J = 6.4 Hz, 3H), 0.82 (d, J = 6.4 Hz, 3H); ^{13}C NMR (150 MHz, $CDCl_3$) δ 164.62, 138.13, 129.92, 128.36, 127.70, 77.13, 74.94, 71.53, 52.44, 50.35, 41.02, 38.74, 35.51, 29.54, 27.43, 25.60, 21.76, 18.44, 18.39, 16.99, 16.40, 12.60.

*1.9. (1R,3aR,4S,7aR)-1-((S)-1-isobutoxypropan-2-yl)-7a-methyloctahydro-1H-inden-4-yl 4-fluorobenzoate (**3f**).* Yield: 72.8%; white powder; mp: 164-166°C. HRMS: *m/z* 391.2654 [M+H]$^+$. ^{1}H NMR (600 MHz, $CDCl_3$) δ 8.02-7.95 (m, 2H), 7.04 (t, J = 8.6 Hz, 2H), 5.33 (d, J = 2.1 Hz, 1H), 3.30 (dd, J = 9.1, 3.3 Hz, 1H), 3.12 (dd, J = 9.2, 6.5 Hz, 1H), 3.02 (ddd, J = 14.3, 9.0, 7.4 Hz, 2H), 2.00 (d, J = 12.7 Hz, 1H), 1.92 (d, J = 15.2 Hz, 1H), 1.81-1.76 (m, 1H), 1.76-1.68 (m, 2H), 1.62-1.57 (m, 1H), 1.50-1.44 (m, 3H), 1.41-1.35 (m, 1H), 1.23 (dd, J = 19.9, 8.4 Hz, 1H), 1.18-1.12 (m, 2H), 0.98 (d, J = 6.6 Hz, 3H), 0.97 (s, 3H), 0.83 (d, J = 6.4 Hz, 3H), 0.82 (d, J = 6.5 Hz, 3H); ^{13}C NMR (150 MHz, $CDCl_3$) δ 164.53, 163.79, 131.05, 130.99, 126.16, 114.54, 114.39, 77.13, 74.95, 71.37, 52.45, 50.36, 41.03, 38.76, 35.52, 29.56, 27.43, 25.61, 21.76, 18.44, 18.39, 17.00, 16.40, 12.61.

*1.10. (1R,3aR,4S,7aR)-1-((S)-1-isobutoxypropan-2-yl)-7a-methyloctahydro-1H-inden-4-yl 3,5-dinitrobenzoate (**3g**).* Yield: 86.4%; light yellow powder; mp: 182-185°C. HRMS: *m/z* 463.2464 [M+H]$^+$. ^{1}H NMR (600 MHz, $CDCl_3$) δ 9.22 (t, J = 2.1 Hz, 1H), 9.17 (d, J = 2.1 Hz, 2H), 5.51 (d, J = 2.1 Hz, 1H), 3.36 (dd, J = 9.1, 3.3 Hz, 1H), 3.19 (dd, J = 9.2, 6.5 Hz, 1H), 3.14 (dd, J = 9.0, 7.3 Hz, 1H), 3.08 (dd, J = 9.2, 6.9 Hz, 1H), 2.12 (d, J = 13.2 Hz, 1H), 2.04 (d, J = 14.1 Hz, 1H), 1.89-1.78 (m, 3H), 1.71-1.66 (m, 1H), 1.65-1.63 (m, 1H), 1.62-1.60 (m, 1H), 1.60-1.54 (m, 2H), 1.47-1.40 (m, 1H), 1.40-1.34 (m, 1H), 1.32 (dd, J = 13.4, 3.2 Hz, 1H), 1.28 (d, J = 9.5 Hz, 1H), 1.09 (s, 3H), 1.06 (d, J = 6.6 Hz, 3H), 0.91 (d, J = 4.7 Hz, 3H), 0.89 (d, J = 4.7 Hz, 3H); ^{13}C NMR (150 MHz, $CDCl_3$) δ 162.39, 148.76, 134.60, 129.35, 122.16, 78.16, 75.88, 75.02, 53.38, 51.21, 42.08, 39.47, 36.44, 30.41, 28.46, 26.51, 22.80, 19.46, 19.42, 17.90, 17.42, 13.63.

*1.11. (1R,3aR,4S,7aR)-1-((S)-1-isobutoxypropan-2-yl)-7a-methyloctahydro-1H-inden-4-yl 3,5-diaminobenzoate (**3h**).* Yield: 61.8% from **3g**; white powder; mp: decomposed at 178°C. HRMS: *m/z* 403.2937 [M+H]$^+$. ^{1}H NMR (600 MHz, DMSO-d_6) δ 6.43 (d, J = 1.8 Hz, 2H), 6.03 (t, J = 1.8 Hz, 1H), 5.21 (d, J = 2.2 Hz, 1H), 4.97 (s, 4H), 3.30 (dd, J = 9.1, 3.2 Hz, 1H), 3.14 (dd, J = 9.2, 6.6 Hz, 1H), 3.11-3.07 (m, 1H), 3.05 (dd, J = 9.1, 6.7 Hz, 1H), 1.97 (d, J = 12.7 Hz, 1H), 1.79 (dd, J = 13.2, 6.8 Hz, 2H), 1.77-1.70 (m, 2H), 1.58 (ddd, J = 16.3, 8.1, 4.6 Hz, 1H), 1.53 (dd, J =

12.7, 9.6 Hz, 2H), 1.50-1.42 (m, 2H), 1.38-1.31 (m, 1H), 1.29-1.21 (m, 2H), 1.21-1.16 (m, 1H), 0.99 (d, J = 6.6 Hz, 3H), 0.98 (s, 3H), 0.86 (d, J = 2.4 Hz, 3H), 0.85 (d, J = 2.6 Hz, 3H); ^{13}C NMR (150 MHz, DMSO-d_6) δ 166.64, 149.24, 131.35, 103.72, 103.61, 77.12, 75.19, 70.55, 52.75, 50.62, 41.39, 35.93, 30.05, 28.01, 27.93, 26.19, 22.37, 19.26, 19.23, 17.64, 17.28, 13.49.

*1.12. (1R,3aR,4S,7aR)-1-((S)-1-isobutoxypropan-2-yl)-7a-methyloctahydro-1H-inden-4-yl 3-hydroxybenzoate (**3i**).* Yield: 62.7% for two steps; white powder; mp: 172-175°C. HRMS: *m/z* 389.2646 [M+H]$^+$. ^{1}H NMR (600 MHz, DMSO-d_6) δ 9.86 (s, 1H), 7.43 (d, J = 7.7 Hz, 1H), 7.41-7.39 (m, 1H), 7.36 (t, J = 7.9 Hz, 1H), 7.06 (dd, J = 7.7, 2.1 Hz, 1H), 5.31 (d, J = 2.5 Hz, 1H), 3.34 (dd, J = 9.1, 3.5 Hz, 1H), 3.18 (dd, J = 9.1, 6.5 Hz, 1H), 3.12 (dd, J = 8.9, 7.3 Hz, 1H), 3.08 (dd, J = 9.1, 6.7 Hz, 1H), 2.02 (d, J = 11.8 Hz, 1H), 1.88 (d, J = 13.5 Hz, 1H), 1.82 (dd, J = 13.2, 6.6 Hz, 1H), 1.78-1.74 (m, 1H), 1.62 (d, J = 12.7 Hz, 3H), 1.55-1.48 (m, 2H), 1.44 (s, 1H), 1.40-1.36 (m, 1H), 1.35-1.32 (m, 1H), 1.29-1.27 (m, 1H), 1.27-1.23 (m, 1H), 1.03 (d, J = 4.0 Hz, 3H), 1.03 (d, J = 2.2 Hz, 3H), 0.90 (d, J = 2.9 Hz, 3H), 0.88 (s, 3H). ^{13}C NMR (150 MHz, DMSO-d_6) δ 165.53, 157.50, 131.45, 129.77, 120.11, 119.68, 115.59, 77.12, 75.16, 71.45, 52.73, 50.47, 41.38, 35.88, 29.89, 27.93, 26.11, 25.78, 22.29, 19.27, 19.23, 17.56, 17.28, 13.35.

*1.13. (1R,3aR,4S,7aR)-1-((S)-1-isobutoxypropan-2-yl)-7a-methyloctahydro-1H-inden-4-yl 3,5-dihydroxybenzoate (**3j**).* Yield: 63.8% for two steps; white powder; mp: decomposed at 180°C. HRMS: *m/z* 405.2638 [M+H]$^+$. ^{1}H NMR (600 MHz, DMSO-d_6) δ 9.65 (s, 2H), 6.87 (d, J = 2.1 Hz, 2H), 6.47 (t, J = 2.2 Hz, 1H), 5.27 (d, J = 2.5 Hz, 1H), 3.34 (dd, J = 9.2, 3.3 Hz, 1H), 3.18 (dd, J = 9.1, 6.5 Hz, 1H), 3.12 (dd, J = 8.9, 7.3 Hz, 1H), 3.08 (dd, J = 9.1, 6.7 Hz, 1H), 2.04-1.99 (m, 1H), 1.89-1.84 (m, 1H), 1.81 (dd, J = 13.3, 6.7 Hz, 1H), 1.79 – 1.72 (m, 1H), 1.64-1.55 (m, 3H), 1.54-1.47 (m, 2H), 1.39-1.34 (m, 1H), 1.34-1.28 (m, 1H), 1.28-1.20 (m, 2H), 1.03 (d, J = 6.8 Hz, 3H), 1.02 (s, 3H), 0.90 (d, J = 2.8 Hz, 3H), 0.89 (d, J = 2.9 Hz, 3H); ^{13}C NMR (150 MHz, DMSO-d_6) δ 165.60, 158.47, 131.95, 107.11, 106.99, 77.12, 75.18, 71.29, 52.74, 50.48, 41.37, 35.88, 29.90, 27.93, 26.12, 22.29, 19.26, 19.22, 17.57, 17.28, 13.66, 13.40.

*1.14. (1R,3aR,4S,7aR)-1-((S)-1-isobutoxypropan-2-yl)-7a-methyloctahydro-1H-inden-4-yl 4-hydroxybenzoate (**3k**).* Yield: 65.6% for two steps; white powder; mp: 186-189°C. HRMS: *m/z* 389.2646 [M+H]$^+$. ^{1}H NMR (600 MHz, DMSO-d_6) δ 10.33 (s, 1H), 7.85 (d, J = 8.7 Hz, 2H), 6.90 (d, J = 8.7 Hz, 2H), 5.28 (d, J = 2.5 Hz, 1H), 3.33 (dd, J = 9.1, 3.2 Hz, 1H), 3.17 (dd, J = 9.1, 6.5 Hz, 1H), 3.14-3.10 (m, 1H), 3.08 (dd, J = 9.1, 6.7 Hz, 1H), 2.01 (d, J = 12.6 Hz, 1H), 1.86 (d, J = 14.6 Hz, 1H), 1.82 (dd, J = 13.3, 6.6 Hz, 1H), 1.80-1.71 (m, 2H), 1.64-1.54 (m, 3H), 1.53-1.46 (m, 2H), 1.41-1.35 (m, 1H), 1.34-1.28 (m, 1H), 1.27-1.20 (m, 2H), 1.03 (d, J = 5.5 Hz, 3H), 1.02 (s, 3H), 0.89 (d, J = 2.8 Hz, 3H), 0.88 (d, J = 2.7 Hz, 3H); ^{13}C NMR (150 MHz, DMSO-d_6) δ 165.37, 161.82, 131.23, 120.85, 115.31, 77.12, 75.17, 70.77, 52.76, 50.55, 41.39, 35.87, 30.00, 27.93, 26.11, 25.78, 22.30, 19.25, 19.22, 17.59, 17.26, 13.31.

1.15.

*(1R,3aR,4S,7aR)-1-((S)-1-(2-hydroxy-2-methylpropoxy)propan-2-yl)-7a-methyloctahydro-1H-inden-4-yl benzoate (**4a**).* Yield: 76.8%; white powder; mp: 135-138°C. HRMS: *m/z* 389.2653 $[M+H]^+$. ^{1}H NMR (600 MHz, $CDCl_3$) δ 8.08 – 8.03 (m, 2H), 7.56 (t, J = 7.4 Hz, 1H), 7.45 (t, J = 7.7 Hz, 2H), 5.42 (s, 1H), 3.43 (dd, J = 9.0, 3.3 Hz, 1H), 3.26 (d, J = 8.7 Hz, 1H), 3.23-3.21 (m, 1H), 3.17 (d, J = 8.7 Hz, 1H), 2.37 (s, 1H), 2.10-2.05 (m, 1H), 2.04-1.98 (m, 1H), 1.84-1.77 (m, 2H), 1.72-1.66 (m, 1H), 1.58-1.52 (m, 2H), 1.50-1.45 (m, 1H), 1.33-1.29 (m, 1H), 1.27-1.24 (m, 2H), 1.21 (s, 3H), 1.21 (s, 3H), 1.18 (s, 3H), 1.07 (s, 2H), 1.06 (d, J = 4.9 Hz, 3H); ^{13}C NMR (150 MHz, $CDCl_3$) δ 166.48, 132.72, 130.86, 129.55, 128.37, 79.29, 76.51, 72.08, 70.33, 53.35, 51.37, 42.01, 39.80, 36.43, 30.55, 26.66, 26.14, 26.13, 22.73, 18.01, 17.41, 13.62.

1.16.

*(1R,3aR,4S,7aR)-1-((S)-1-(2-hydroxy-2-methylpropoxy)propan-2-yl)-7a-methyloctahydro-1H-inden-4-yl 4-nitrobenzoate (**4b**).* Yield: 74.5%; white powder; mp: 161-164°C. HRMS: *m/z* 434.2547 $[M+H]^+$. ^{1}H NMR (600 MHz, $CDCl_3$) δ 8.30 (d, J = 8.9 Hz, 2H), 8.21 (d, J = 8.9 Hz, 2H), 5.45 (s, 1H), 3.43 (dd, J = 9.0, 3.4 Hz, 1H), 3.25 (dd, J = 8.7, 3.7 Hz, 1H), 3.24-3.22 (m, 1H), 3.19-3.16 (m, 1H), 2.32 (s, 1H), 2.11-2.06 (m, 1H), 2.03 (dd, J = 11.9, 7.4 Hz, 1H), 1.86-1.78 (m, 2H), 1.64-1.55 (m, 4H), 1.48-1.40 (m, 1H), 1.30-1.25 (m, 2H), 1.21 (s, 3H), 1.21 (s, 3H), 1.18 (s, 3H), 1.07 (s, 2H), 1.04 (d, J = 4.9 Hz, 3H); ^{13}C NMR (150 MHz, $CDCl_3$) δ 164.59, 150.45, 136.25, 130.62, 123.60, 79.37, 76.49, 73.51, 70.35, 53.33, 51.27, 42.03, 39.64, 36.43, 30.45, 26.58, 26.15, 22.75, 17.95, 17.43, 13.64.

1.17.

*(1R,3aR,4S,7aR)-1-((S)-1-(2-hydroxy-2-methylpropoxy)propan-2-yl)-7a-methyloctahydro-1H-inden-4-yl 4-methoxybenzoate (**4c**).* Yield: 79.5%; white powder; mp: 156-158°C. HRMS: *m/z* 419.2743 $[M+H]^+$. ^{1}H NMR (600 MHz, $CDCl_3$) δ 8.00 dd, J = 9.3, 2.3 Hz, 2H), 6.93 (dd, J = 9.3, 2.3 Hz, 2H), 5.39 (s, 1H), 3.86 (s, 3H), 3.43 (dd, J = 9.0, 3.4 Hz, 1H), 3.25 (d, J = 8.8 Hz, 1H), 3.21 (dd, J = 8.5, 7.0 Hz, 1H), 3.18-3.15 (m, 1H), 2.35 (s, 1H), 2.08-2.04 (m, 1H), 2.01-1.96 (m, 1H), 1.83-1.77 (m, 2H), 1.72-1.67 (m, 1H), 1.58-1.51 (m, 4H), 1.33-1.29 (m, 1H), 1.27-1.24 (m, 1H), 1.21 (s, 3H), 1.20 (s, 3H), 1.18 (s, 2H), 1.06 (d, J = 6.7 Hz, 3H), 1.05 (s, 2H); ^{13}C NMR (150 MHz, $CDCl_3$) δ 166.25, 163.20, 131.54, 123.35, 113.62, 79.34, 76.56, 71.66, 70.33, 55.43, 53.41, 51.44, 42.03, 39.86, 36.45, 30.62, 26.68, 26.16, 26.15, 22.75, 18.05, 17.42, 13.63.

1.18.

*(1R,3aR,4S,7aR)-1-((S)-1-(2-hydroxy-2-methylpropoxy)propan-2-yl)-7a-methyloctahydro-1H-inden-4-yl 3-methoxybenzoate (**4d**).* Yield: 72.7%; white powder; mp: 153-155°C. HRMS: *m/z* 419.2743 $[M+H]^+$. ^{1}H NMR (600 MHz, $CDCl_3$) δ 7.65 (d, J = 7.7 Hz, 1H), 7.60-7.57 (m, 1H), 7.35 (t, J = 7.9 Hz, 1H), 7.12-7.08 (m, 1H), 5.41 (s, 1H), 3.85 (s, 3H), 3.43 (dd, J = 9.1, 3.4 Hz,

1H), 3.25 (d, J = 8.7 Hz, 1H), 3.23-3.20 (m, 1H), 3.17 (d, J = 8.8 Hz, 1H), 2.33 (s, 1H), 2.09-2.05 (m, 1H), 2.03-1.97 (m, 1H), 1.84-1.77(m, 2H), 1.72-1.66 (m, 1H), 1.57-1.51(m, 3H), 1.34-1.30 (m, 1H), 1.27-1.23 (m, 2H), 1.21 (s, 3H), 1.20 (s, 3H), 1.18 (s, 2H), 1.07 (s, 3H), 1.06 (d, J = 6.6 Hz, 3H), ^{13}C NMR (150 MHz, $CDCl_3$) δ 166.32, 159.56, 132.21, 129.40, 121.95, 119.26, 114.05, 79.33, 76.54, 72.19, 70.34, 55.38, 53.39, 51.40, 42.04, 39.81, 36.46, 30.54, 26.67, 26.16, 22.74, 18.02, 17.41, 13.61.

1.19.

*(1R,3aR,4S,7aR)-1-((S)-1-(2-hydroxy-2-methylpropoxy)propan-2-yl)-7a-methyloctahydro-1H-inden-4-yl 4-chlorobenzoate (**4e**).* Yield: 68.9%; white powder; mp: 165-168°C. HRMS: *m/z* 423.2256 $[M+H]^+$. ^{1}H NMR (600 MHz, $CDCl_3$) δ 7.98 (dd, J = 8.8, 2.1 Hz, 2H), 7.42 (dd, J = 8.8, 2.1 Hz, 2H), 5.41 (s, 1H), 3.43 (dd, J = 9.1, 3.4 Hz, 1H), 3.26-3.24 (m, 1H), 3.23-3.20 (m, 1H), 3.17 (d, J = 8.7 Hz, 1H), 2.32 (s, 1H), 2.10-2.05 (m, 1H), 2.02-1.96 (m, 1H), 1.85-1.76 (m, 1H), 1.72-1.66 (m, 1H), 1.57-1.52 (m, 3H), 1.34-1.29 (m, 1H), 1.28-1.23 (m, 2H), 1.21 (s, 3H), 1.20 (s, 3H), 1.17 (s, 1H), 1.06 (d, J = 6.6 Hz, 3H), 1.03 (s, 3H); ^{13}C NMR (150 MHz, $CDCl_3$) δ 165.63, 139.17, 130.93, 129.34, 128.73, 79.35, 76.52, 72.49, 70.34, 53.37, 51.35, 42.03, 39.76, 36.44, 30.52, 26.63, 26.16, 26.15, 22.74, 18.00, 17.42, 13.62.

1.20.

*(1R,3aR,4S,7aR)-1-((S)-1-(2-hydroxy-2-methylpropoxy)propan-2-yl)-7a-methyloctahydro-1H-inden-4-yl 3,5-dinitrobenzoate (**4f**).* Yield: 66.9%; light yellow powder; mp: 194-196°C. HRMS: *m/z* 479.2354 $[M+H]^+$. ^{1}H NMR (600 MHz, $CDCl_3$) δ 9.23 (t, J = 2.1 Hz, 1H), 9.17 (d, J = 2.1 Hz, 2H), 5.52 (s, 1H), 3.43 (dd, J = 9.1, 3.4 Hz, 1H), 3.26-3.22 (m, 2H), 3.18 (d, J = 8.7 Hz, 1H), 2.33 (s, 1H), 2.12 (d, J = 13.2 Hz, 1H), 2.05 (d, J = 13.6 Hz, 1H), 1.86-1.80 (m, 2H), 1.72 (ddd, J = 13.5, 6.7, 3.4 Hz, 1H), 1.65-1.59 (m, 5H), 1.48-1.40 (m, 1H), 1.37 (ddd, J = 9.7, 6.2, 1.9 Hz, 1H), 1.31 (dd, J = 11.5, 7.6 Hz, 2H), 1.21 (s, 3H), 1.21 (s, 3H), 1.10 (s, 3H), 1.08 (d, J = 6.6 Hz, 3H). ^{13}C NMR (150 MHz, $CDCl_3$) δ 162.37, 148.72, 134.52, 129.36, 122.19, 79.33, 76.41, 74.93, 70.36, 53.27, 51.16, 42.06, 39.44, 36.37, 30.34, 26.52, 26.14, 22.74, 17.87, 17.40, 13.62.

1.21.

*(1R,3aR,4S,7aR)-1-((S)-1-(2-hydroxy-2-methylpropoxy)propan-2-yl)-7a-methyloctahydro-1H-inden-4-yl 3-hydroxy-4-methoxybenzoate (**4g**).* Yield: 64.2% for two steps; white powder; mp: 188-190°C. HRMS: *m/z* 435.2743 $[M+H]^+$. ^{1}H NMR (600 MHz, $CDCl_3$) δ 7.63 (dd, J = 8.4, 2.0 Hz, 1H), 7.59 (d, J = 2.0 Hz, 1H), 6.89 (d, J = 8.5 Hz, 1H), 5.78 (s, 1H), 5.37 (s, 1H), 3.95 (s, 3H), 3.43 (dd, J = 9.1, 3.4 Hz, 1H), 3.25 (d, J = 8.8 Hz, 1H), 3.23-3.19 (m, 1H), 3.17 (d, J = 8.7 Hz, 1H), 2.37 (s, 1H), 2.06 (d, J = 12.7 Hz, 1H), 2.01-1.95 (m, 1H), 1.83-1.76 (m, 2H), 1.72-1.65 (m, 1H), 1.56-1.50 (m, 4H), 1.34-1.26 (m, 2H), 1.21 (s, 3H), 1.21 (s, 3H), 1.19-1.17 (m, 2H), 1.06 (d, J = 5.4 Hz, 3H), 1.05 (s, 3H); ^{13}C NMR (150 MHz, $CDCl_3$) δ 166.21, 150.30, 145.26, 124.15,

122.77, 115.59, 109.91, 79.31, 76.56, 71.85, 70.39, 56.05, 53.40, 51.43, 42.03, 39.86, 36.43, 30.59, 26.67, 26.15, 26.14, 22.75, 18.04, 17.41, 13.67.

1.22.

*(1R,3aR,4S,7aR)-1-((S)-1-(2-hydroxy-2-methylpropoxy)propan-2-yl)-7a-methyloctahydro-1H-inden-4-yl 3-hydroxybenzoate (**4h**).* Yield: 65.6% for two steps; white powder; mp: 183-185°C. HRMS: *m/z* 405.2638 $[M+H]^+$. ^{1}H NMR (600 MHz, $CDCl_3$) δ 7.59 (d, J = 7.7 Hz, 1H), 7.57-7.56 (m, 1H), 7.30 (t, J = 7.9 Hz, 1H), 7.07-7.05 (m, 1H), 6.74 (s, 1H), 5.39 (s, 1H), 3.42 (dd, J = 9.0, 3.5 Hz, 1H), 3.28-3.25 (m, 1H), 3.22-3.20 (m, 1H), 3.19-3.17 (m, 1H), 2.52 (s, 1H), 2.04 (d, J = 11.4 Hz, 1H), 2.01-1.96 (m, 1H), 1.86-1.75 (m, 3H), 1.68-1.64 (m, 1H), 1.57-1.51 (m, 4H), 1.48-1.41 (m, 2H), 1.23 (s, 3H), 1.22 (s, 3H), 1.04 (d, J = 6.6 Hz, 3H), 1.03 (s, 3H); ^{13}C NMR (150 MHz, $CDCl_3$) δ 166.64, 156.27, 132.12, 129.65, 121.63, 120.19, 116.37, 79.21, 76.56, 72.47, 70.75, 53.36, 51.34, 42.02, 39.78, 36.43, 30.51, 26.62, 26.12, 26.09, 22.75, 18.00, 17.40, 13.69.

1.23.

*(1R,3aR,4S,7aR)-1-((S)-1-(2-hydroxy-2-methylpropoxy)propan-2-yl)-7a-methyloctahydro-1H-inden-4-yl 3,5-dihydroxybenzoate (**4i**).* Yield: 56.3% for two steps; white powder; mp: decomposed at 180°C. HRMS: *m/z* 421.2536 $[M+H]^+$. ^{1}H NMR (600 MHz, DMSO-d_6) δ 9.61 (s, 2H), 6.83 (d, J = 2.2 Hz, 2H), 6.44 (t, J = 2.2 Hz, 2H), 5.23 (s, 1H), 3.35 (dd, J = 9.2, 3.3 Hz, 1H), 3.14 (d, J = 8.9 Hz, 1H), 3.12-3.07 (m, 1H), 3.04 (d, J = 8.9 Hz, 1H), 1.98 (d, J = 12.6 Hz, 1H), 1.82 (d, J = 13.8 Hz, 1H), 1.77-1.67 (m, 2H), 1.62 – 1.52 (m, 2H), 1.50-1.48 (m, 1H), 1.34-1.26 (m, 2H), 1.24-1.16 (m, 2H), 1.09 (d, J = 2.6 Hz, 1H), 1.07 (s, 6H), 1.04 (s, 1H), 0.99 (d, J = 6.6 Hz, 3H), 0.98 (s, 3H); ^{13}C NMR (150 MHz, DMSO-d_6) δ 166.10, 158.97, 132.44, 107.61, 107.49, 80.19, 76.34, 71.78, 69.55, 53.27, 50.97, 41.88, 36.36, 30.40, 27.34, 27.30, 27.26, 26.62, 22.81, 18.07, 17.71, 13.91.

1.24.

*(1R,3aR,4S,7aR)-1-((S)-1-(2-hydroxy-2-methylpropoxy)propan-2-yl)-7a-methyloctahydro-1H-inden-4-yl 4-hydroxybenzoate (**4j**).* Yield: 66.8% for two steps; white powder; mp: 190-192°C. HRMS: *m/z* 405.2638 $[M+H]^+$. ^{1}H NMR (600 MHz, $CDCl_3$) δ 7.95 (d, J = 8.7 Hz, 2H), 6.98 (s, 1H), 6.88 (d, J = 8.7 Hz, 2H), 5.38 (s, 1H), 3.44 (dd, J = 9.1, 3.4 Hz, 1H), 3.55-3.28 (m, 1H), 3.22-3.19 (m, 1H), 3.18 (d, J = 8.8 Hz, 1H), 2.55 (s, 1H), 2.08-2.03 (m, 1H), 2.00-1.96 (m, 1H), 1.83-1.76 (m, 3H), 1.71-1.67 (m, 1H), 1.57-1.49 (m, 4H), 1.33-1.27 (m, 2H),1.23 (s, 3H), 1.23 (s, 3H), 1.18 (s, 1H), 1.06 (d, J = 6.7 Hz, 3H), 1.05 (s, 3H); ^{13}C NMR (150 MHz, $CDCl_3$) δ 166.57, 160.35, 131.83, 122.90, 115.28, 79.18, 76.60, 71.84, 70.83, 53.40, 51.41, 42.04, 39.84, 36.45, 30.59, 26.68, 26.09, 26.06, 22.75, 18.04, 17.41, 13.65.

2. ^{1}H-NMR and ^{13}C NMR spectrum

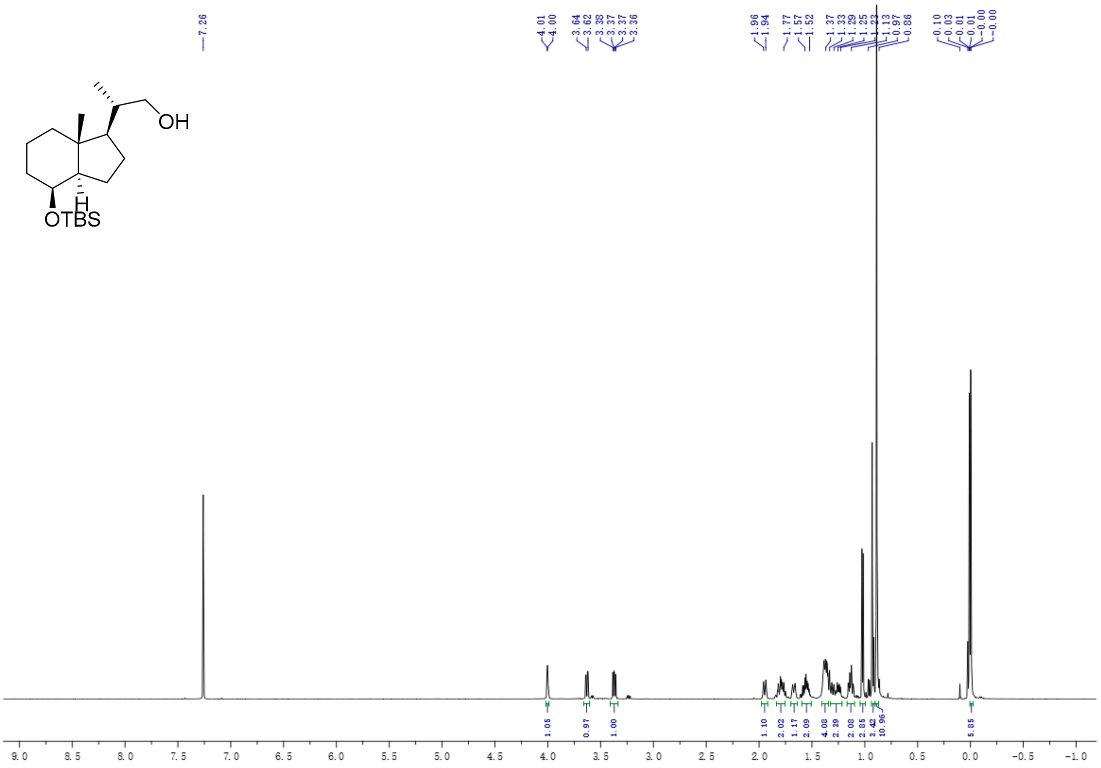

Figure S1. ^{1}H NMR (600 MHz, $CDCl_3$, r.t.) spectrum for **1**

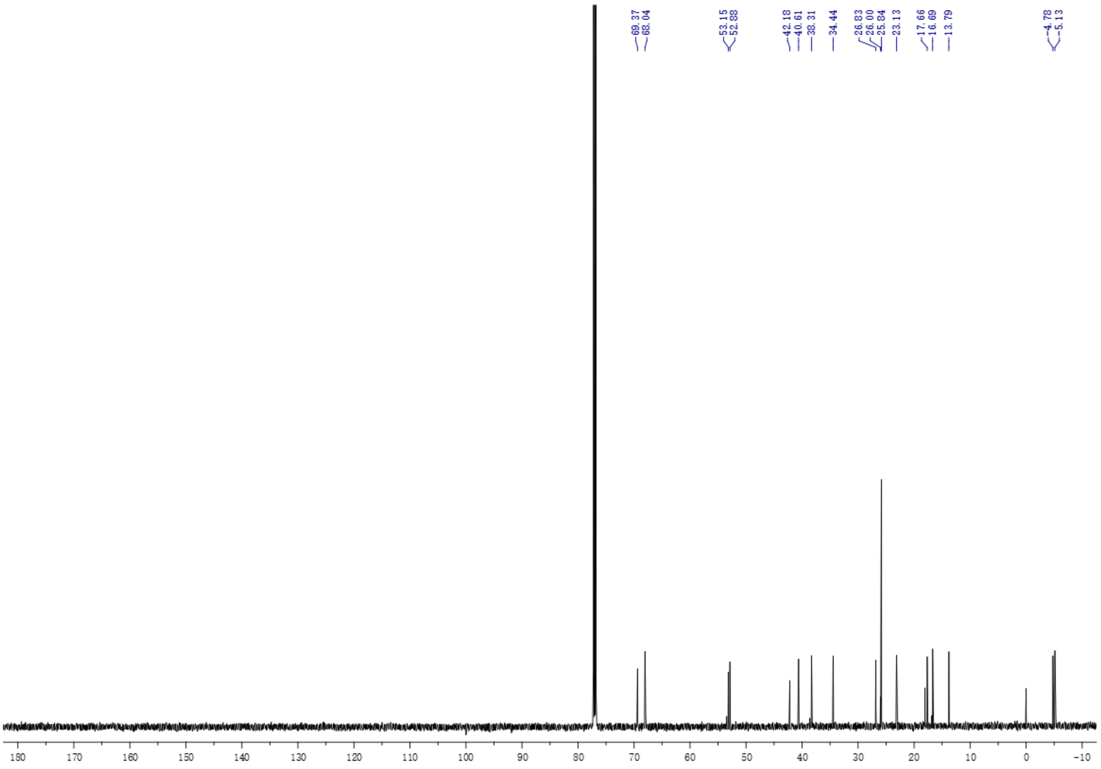

Figure S2. ^{13}C NMR (150 MHz, $CDCl_3$, r.t.) spectrum for **1**

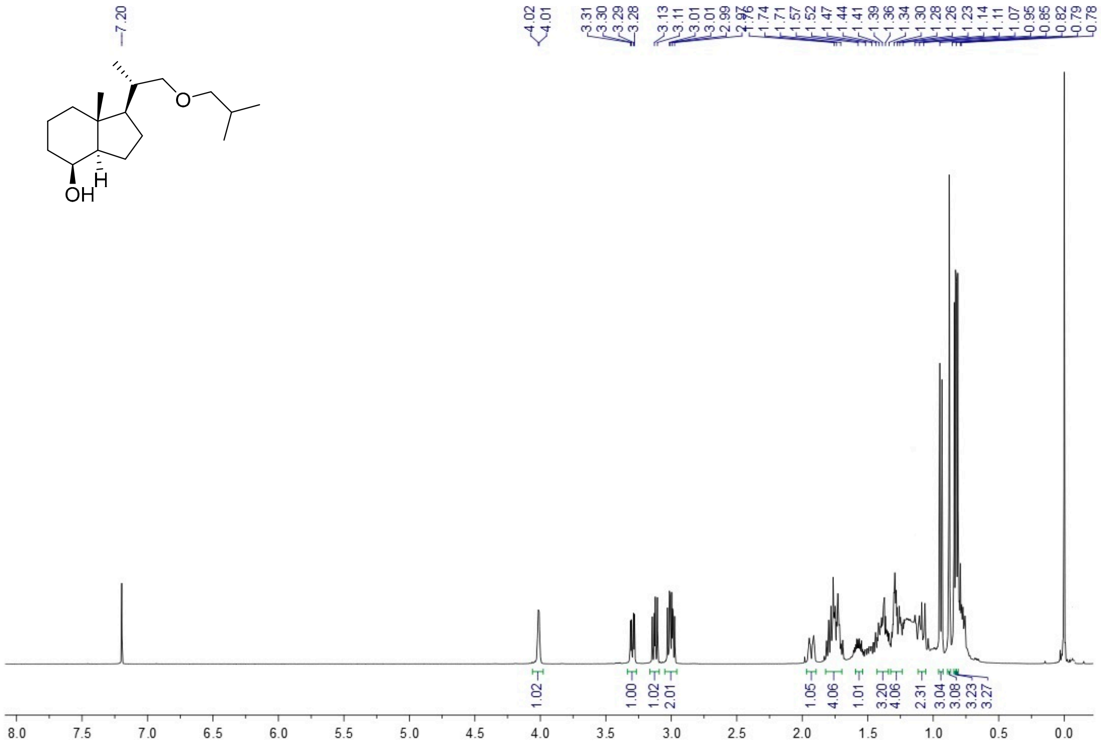

Figure S3. ^{1}H NMR (600 MHz, $CDCl_3$, r.t.) spectrum for **2a**

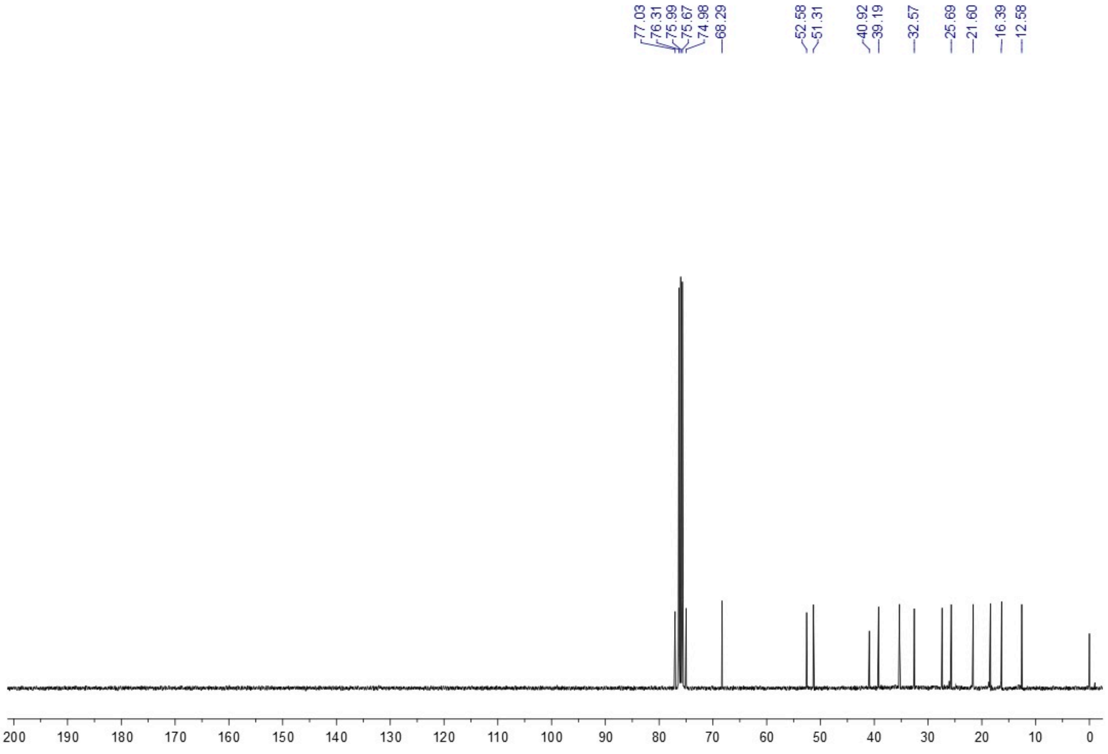

Figure S4. ^{13}C NMR (150 MHz, $CDCl_3$, r.t.) spectrum for **2a**

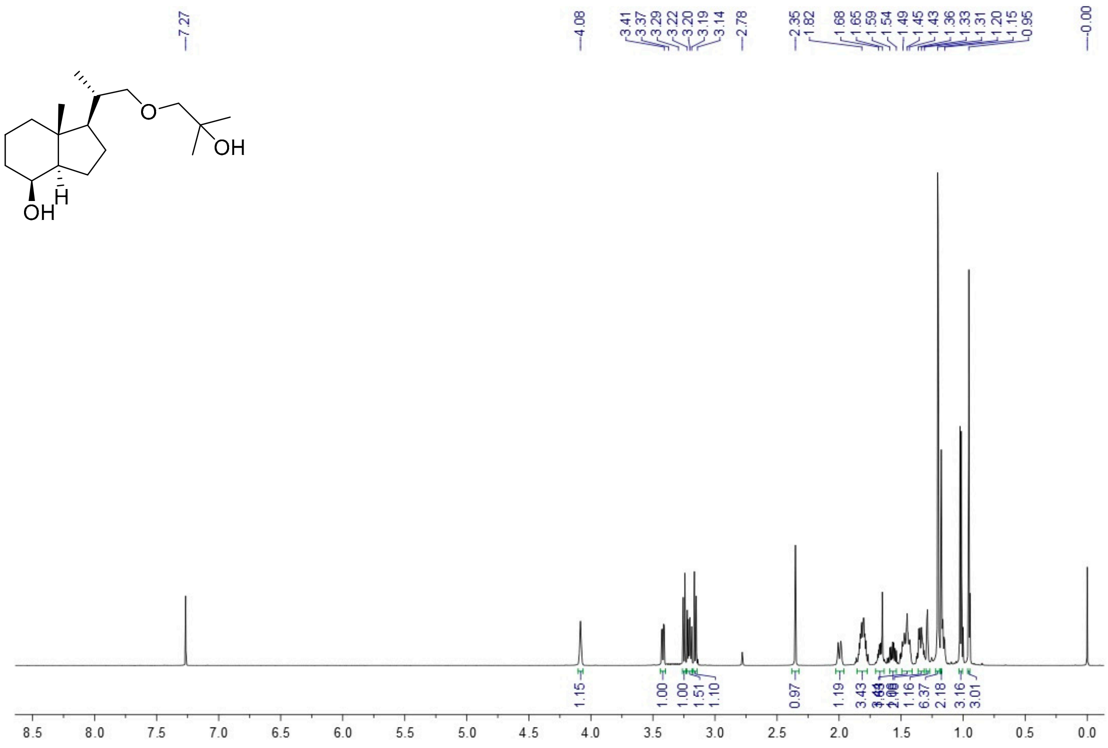

Figure S5. ^{1}H NMR (600 MHz, $CDCl_3$, r.t.) spectrum for **2b**

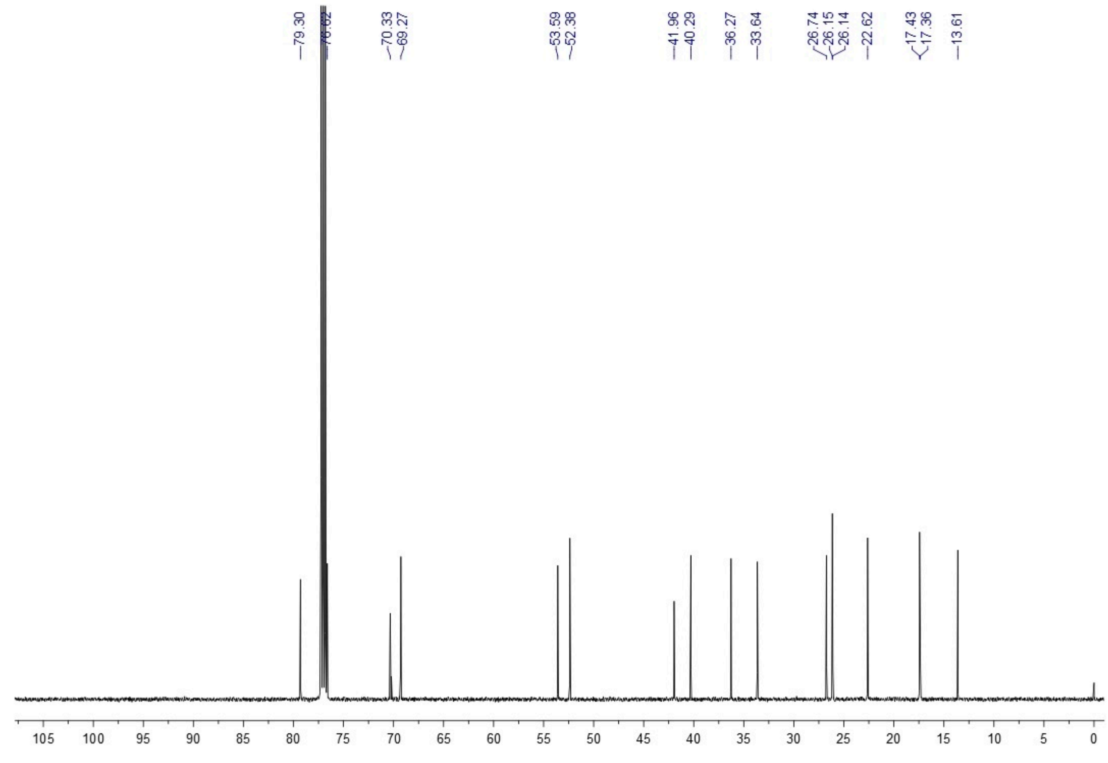

Figure S6. ^{13}C NMR (150 MHz, $CDCl_3$, r.t.) spectrum for **2b**

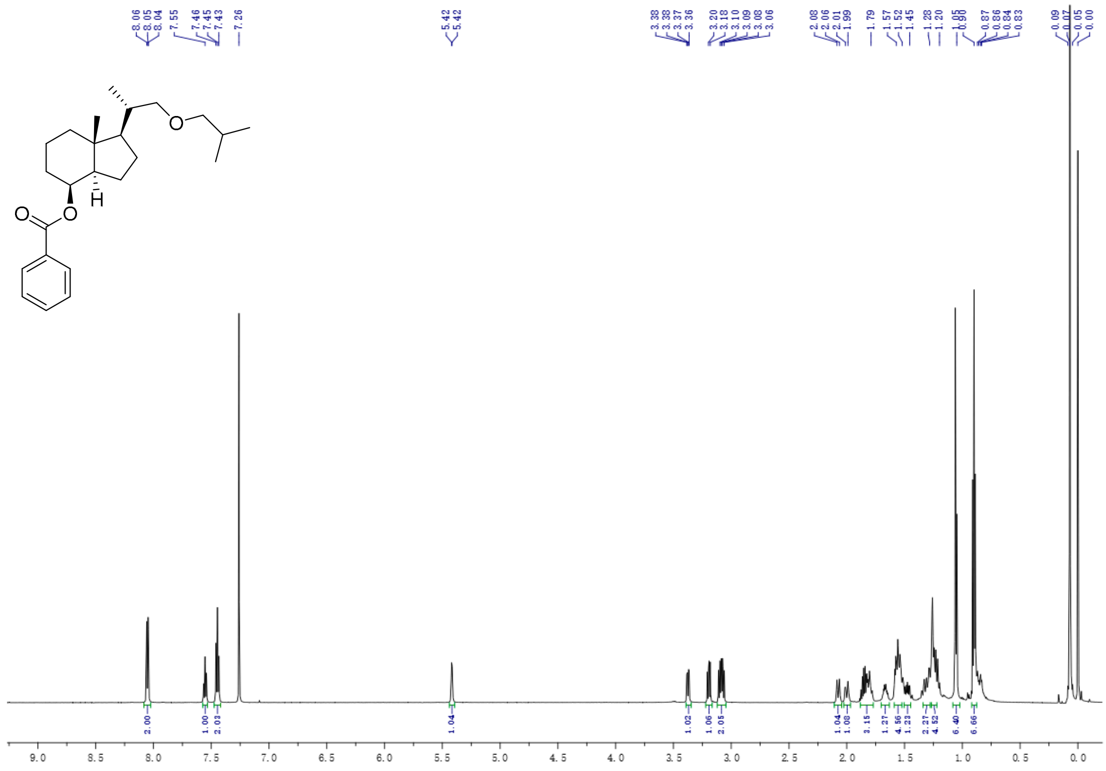

Figure S7. ^{1}H NMR (600 MHz, $CDCl_3$, r.t.) spectrum for **3a**

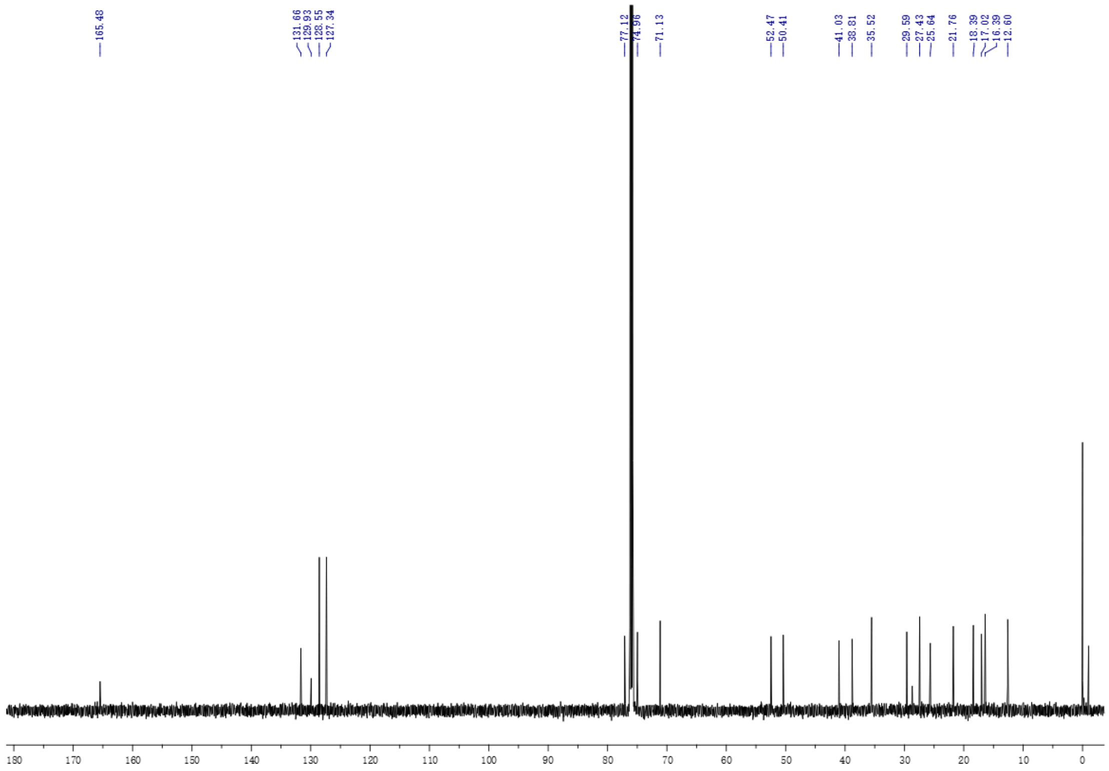

Figure S8. ^{13}C NMR (150 MHz, $CDCl_3$, r.t.) spectrum for **3a**

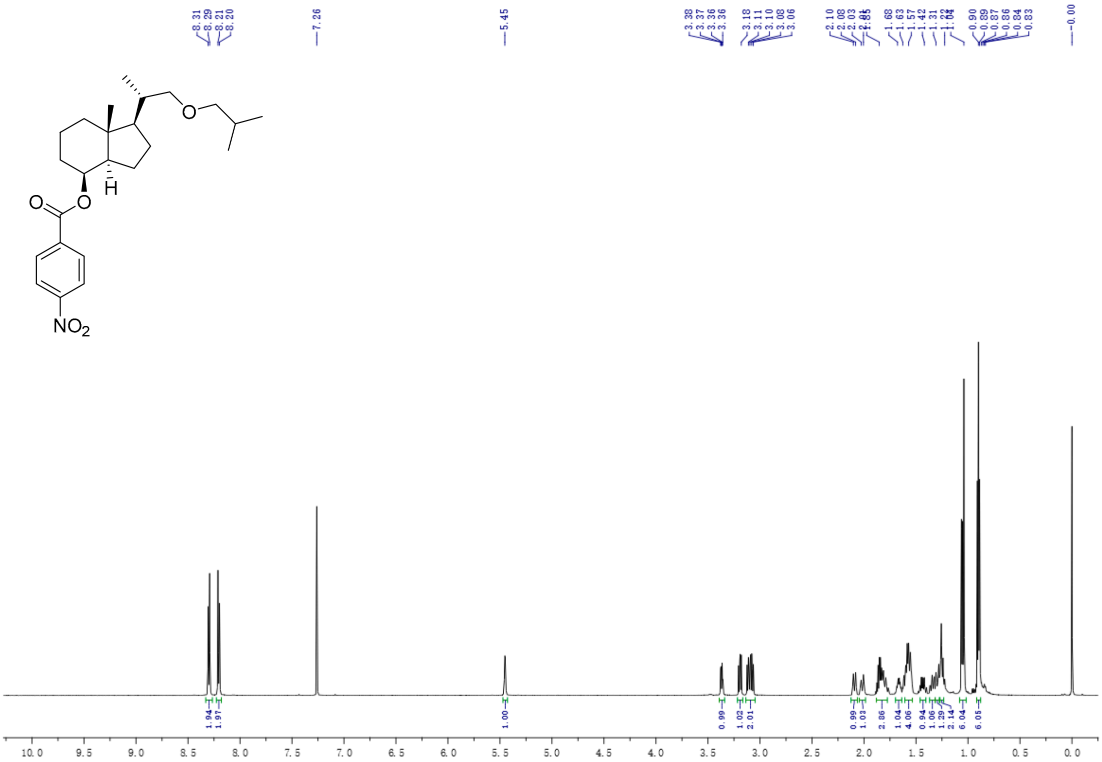

Figure S9. ^{1}H NMR (600 MHz, $CDCl_3$, r.t.) spectrum for **3b**

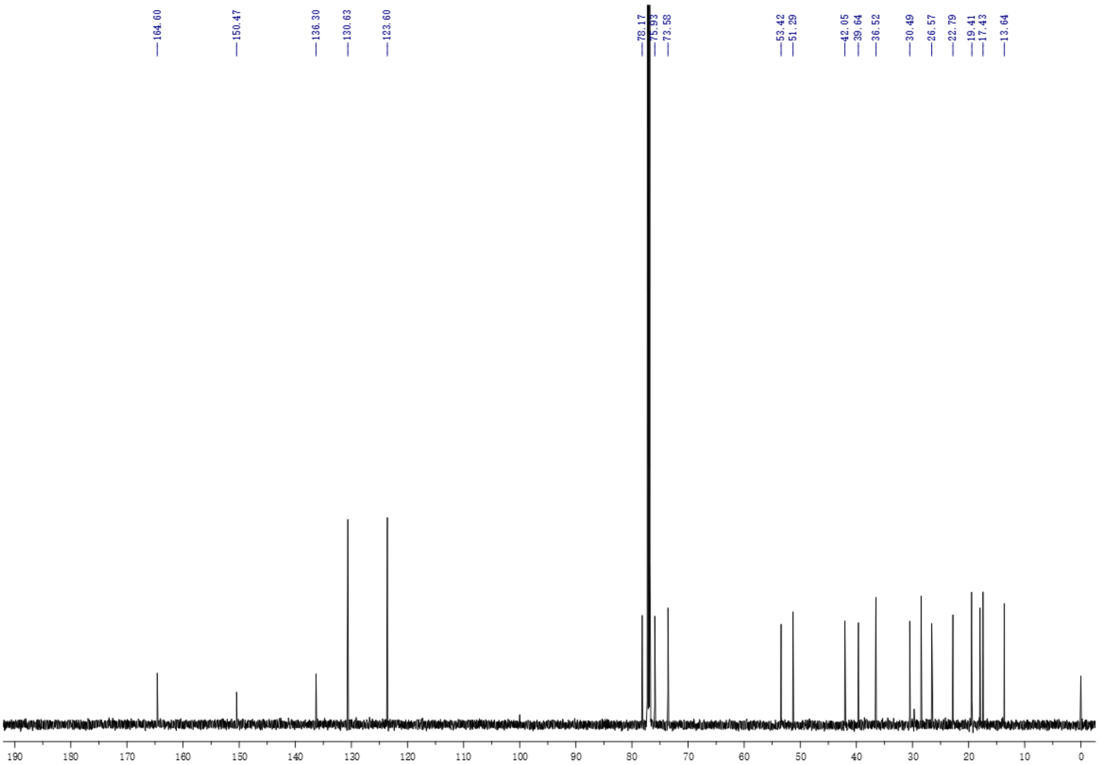

Figure S10. ^{13}C NMR (150 MHz, $CDCl_3$, r.t.) spectrum for **3b**

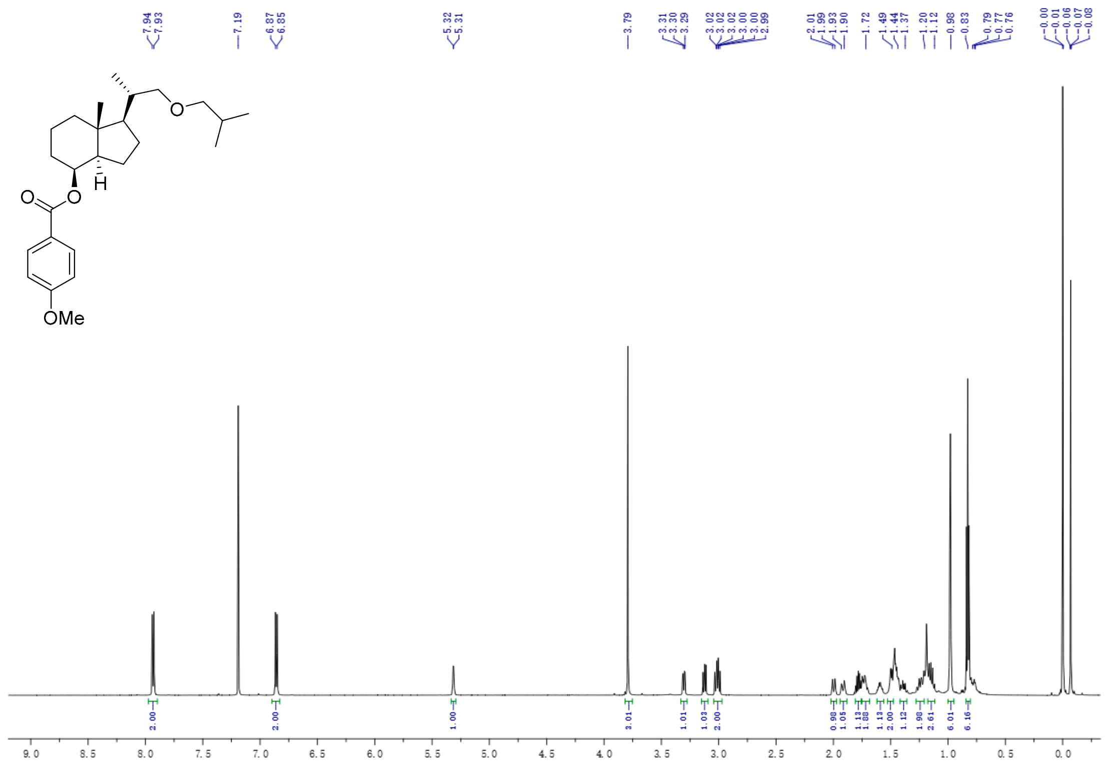

Figure S11. ^{1}H NMR (600 MHz, $CDCl_3$, r.t.) spectrum for **3c**

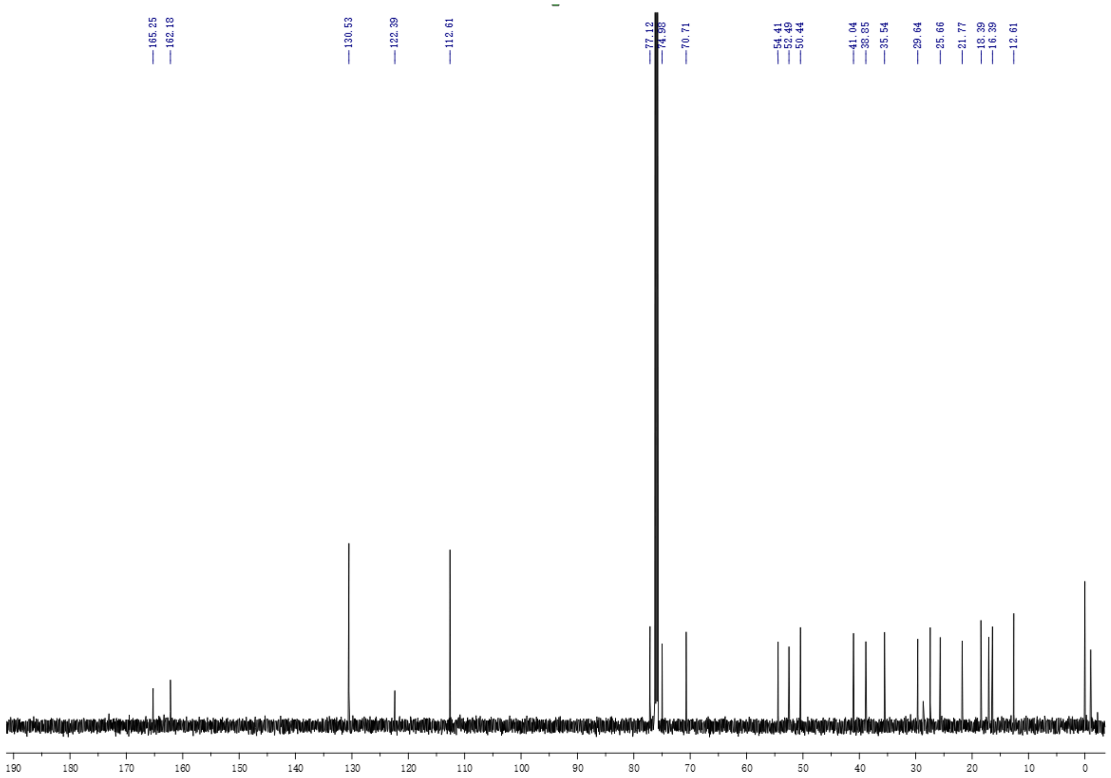

Figure S12. ^{13}C NMR (150 MHz, $CDCl_3$, r.t.) spectrum for 3**c**

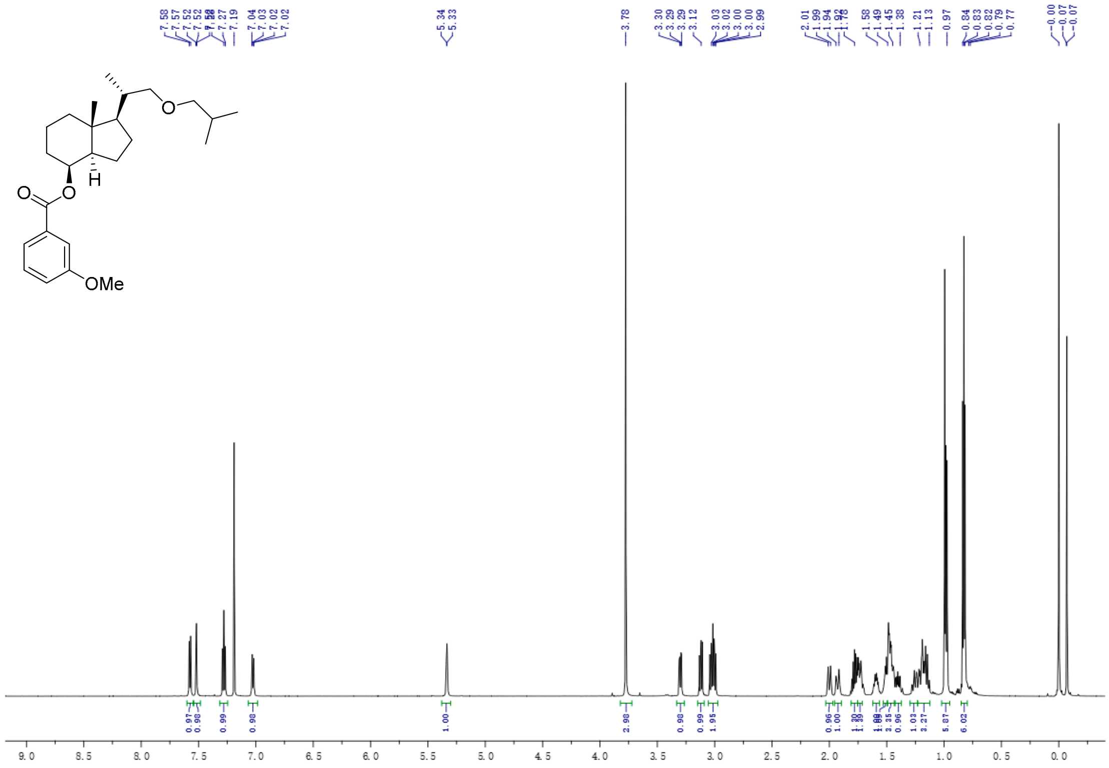

Figure S13. ^{1}H NMR (600 MHz, $CDCl_3$, r.t.) spectrum for **3d**

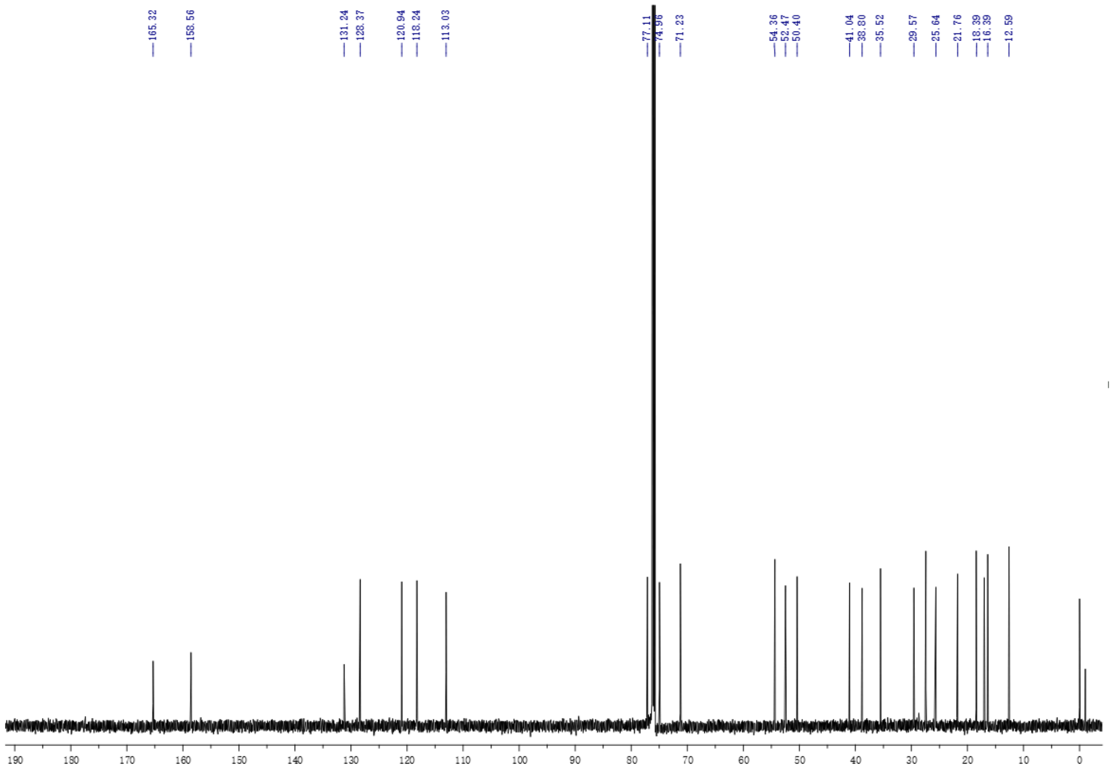

Figure S14. ^{13}C NMR (150 MHz, $CDCl_3$, r.t.) spectrum for **3d**

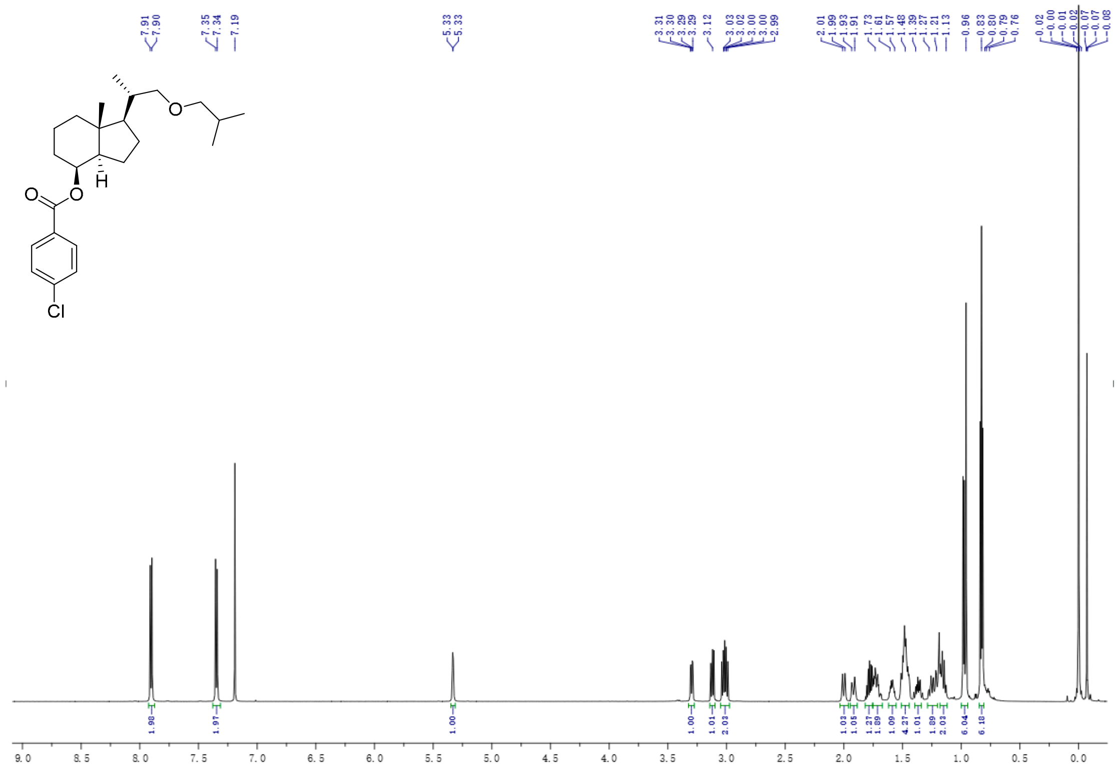

Figure S15. ^{1}H NMR (600 MHz, $CDCl_3$, r.t.) spectrum for **3e**

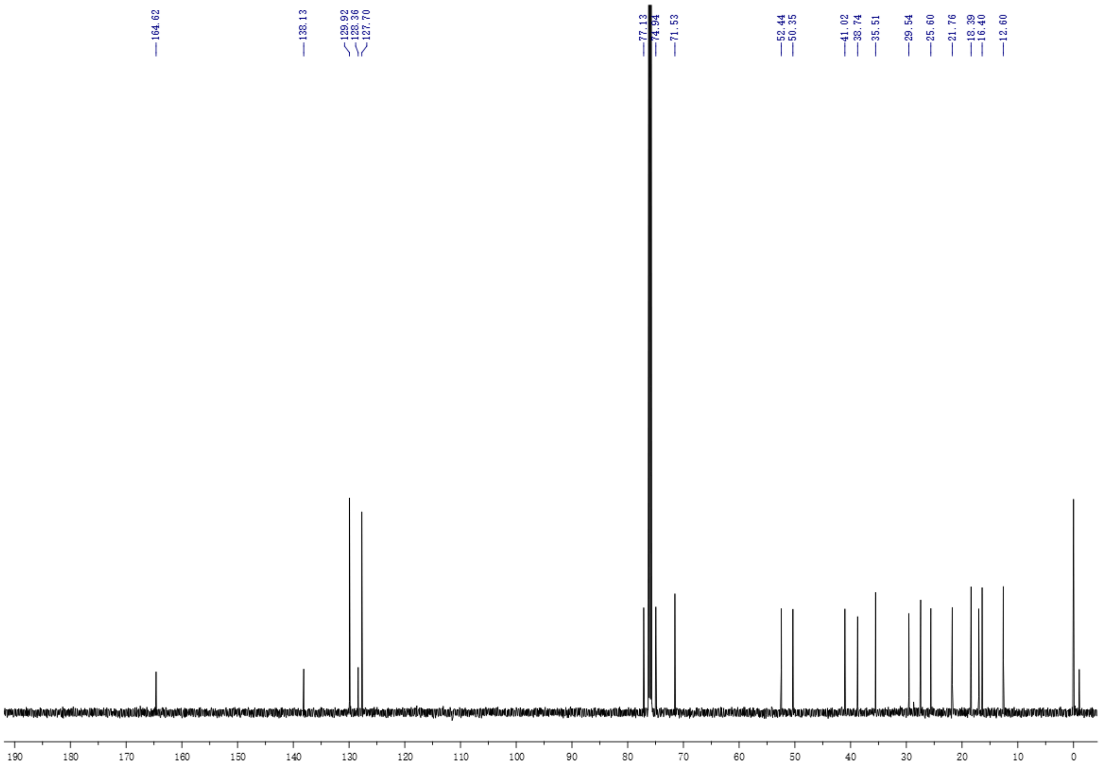

Figure S16. ^{13}C NMR (150 MHz, $CDCl_3$, r.t.) spectrum for **3e**

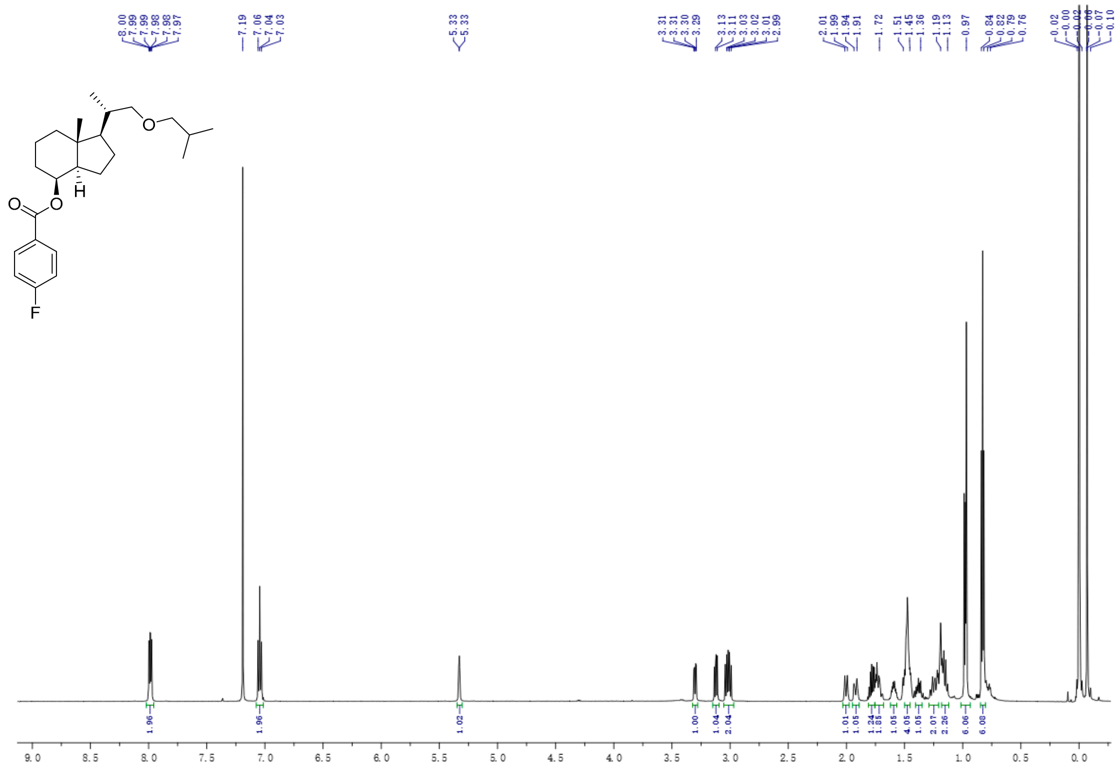

Figure S17. ^{1}H NMR (600 MHz, $CDCl_3$, r.t.) spectrum for **3f**

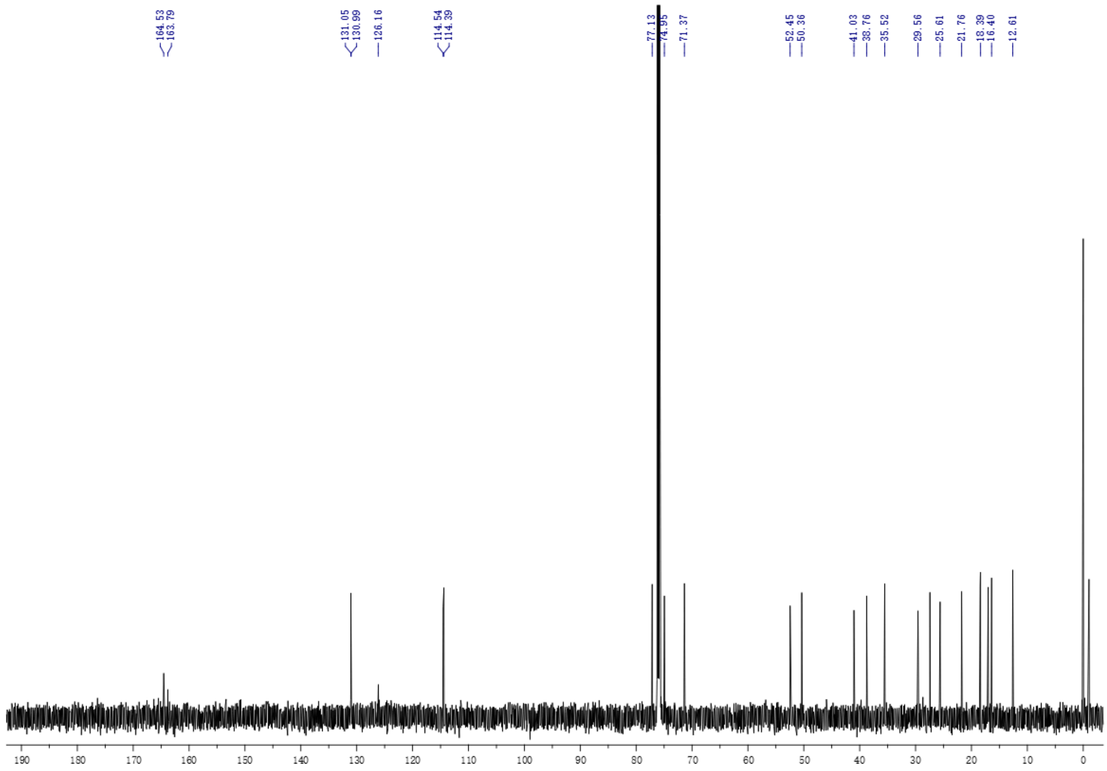

Figure S18. ^{13}C NMR (150 MHz, $CDCl_3$, r.t.) spectrum for **3f**

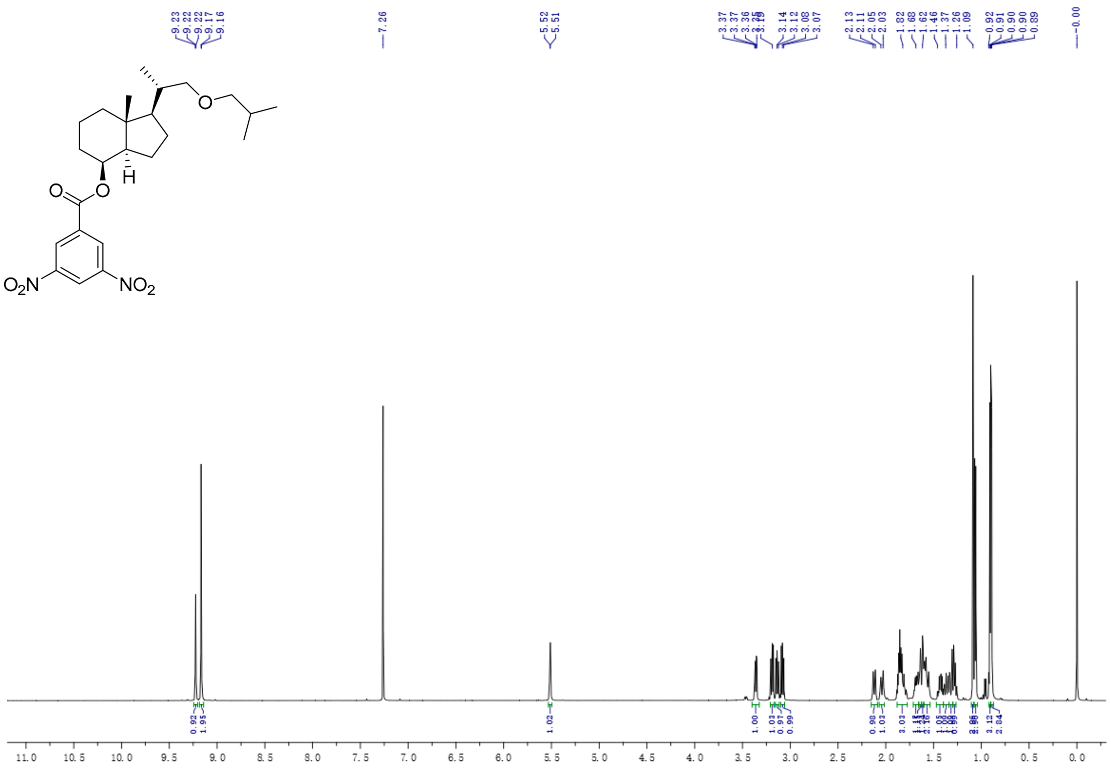

Figure S19. ^{1}H NMR (600 MHz, CDCl$_3$, r.t.) spectrum for **3g**

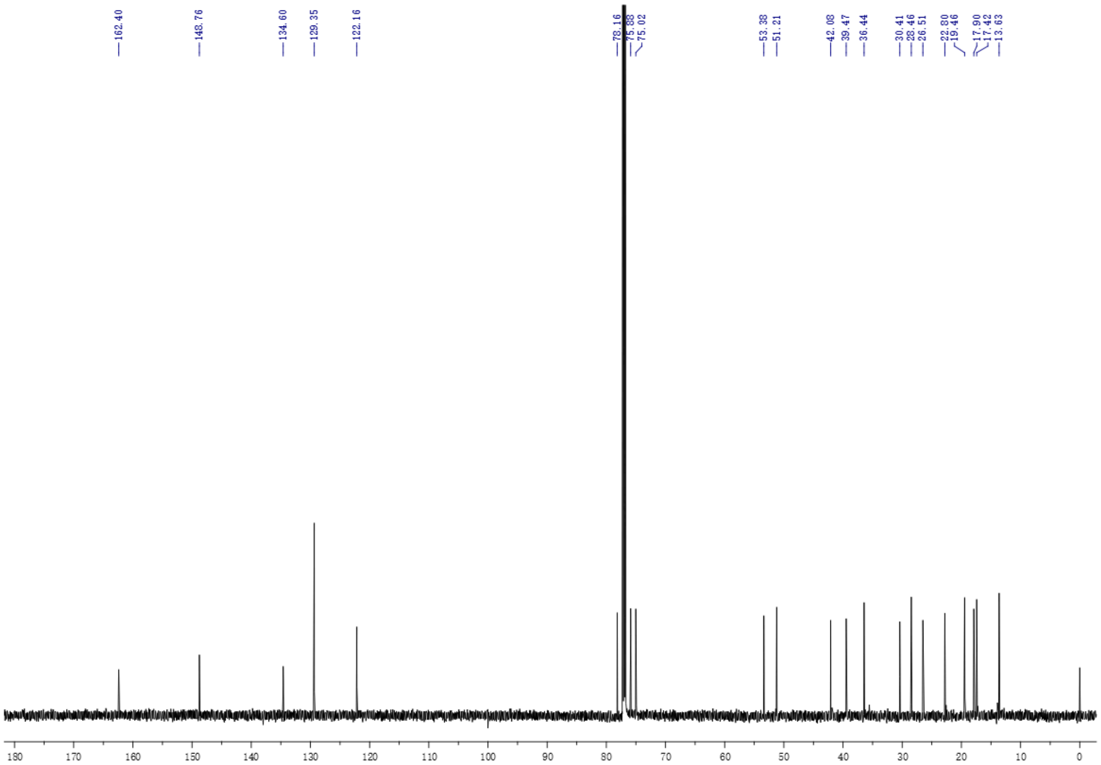

Figure S20. ^{13}C NMR (150 MHz, CDCl$_3$, r.t.) spectrum for **3g**

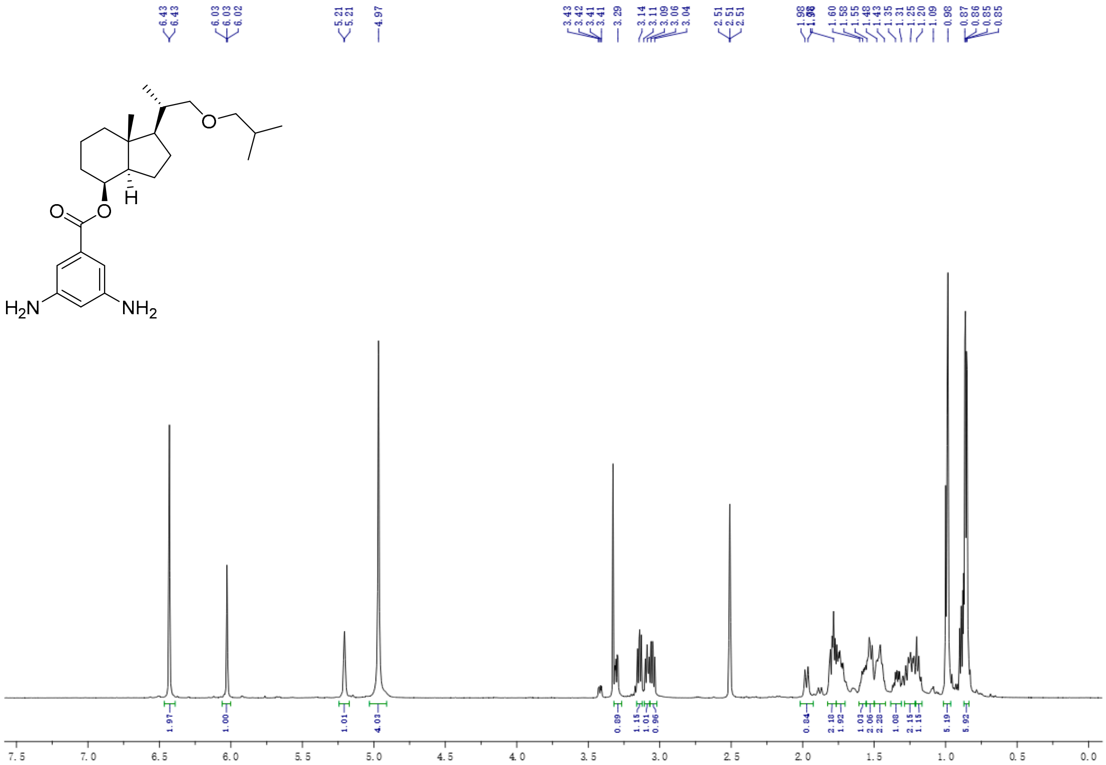

Figure S21. ^{1}H NMR (600 MHz, DMSO-d_6, r.t.) spectrum for **3h**

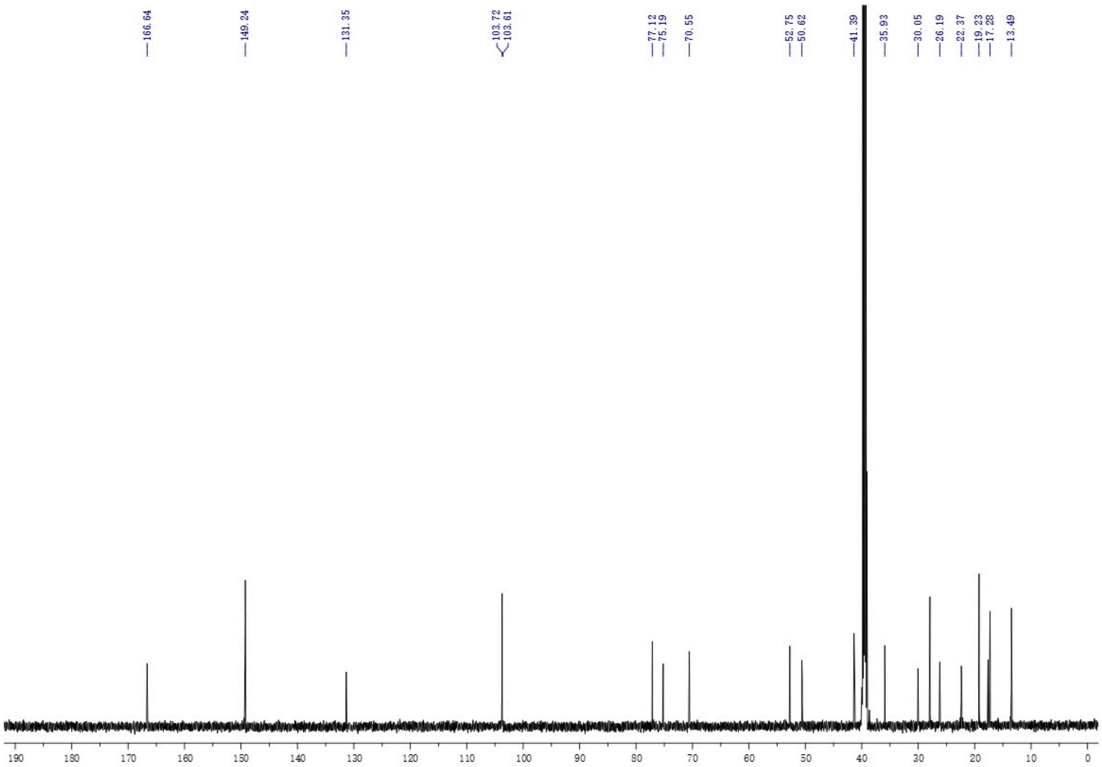

Figure S22. ^{13}C NMR (150 MHz, DMSO-d_6, r.t.) spectrum for **3h**

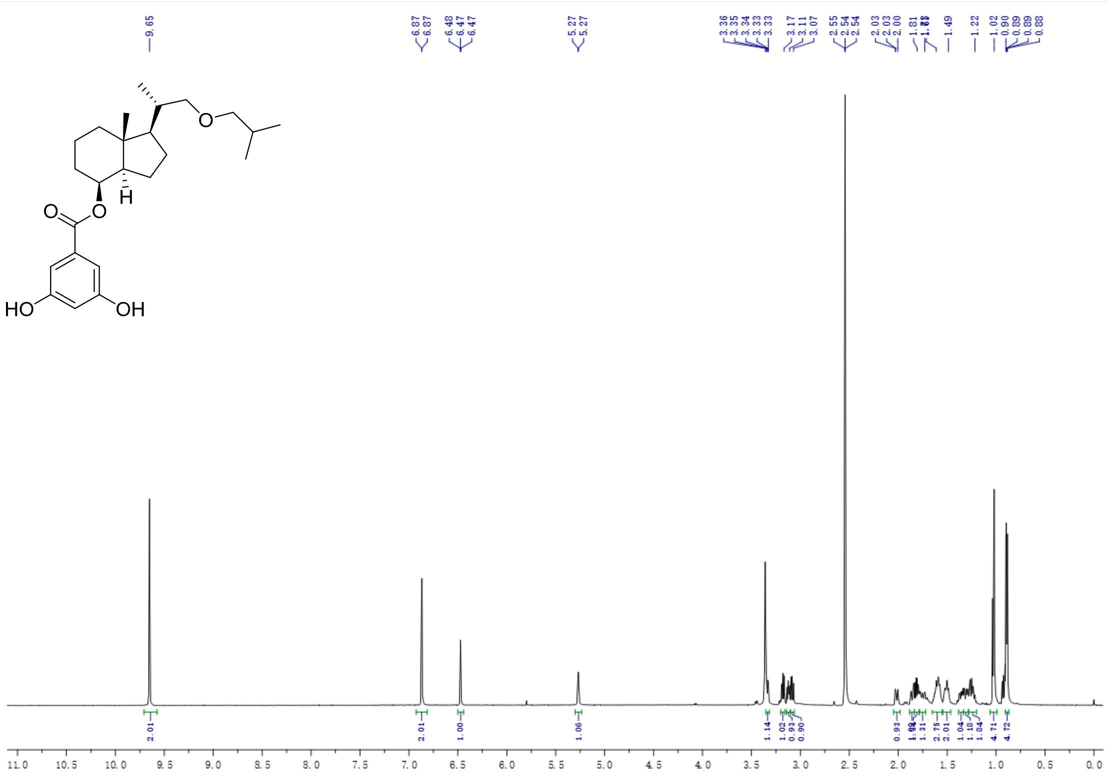

Figure S23. ^{1}H NMR (600 MHz, DMSO-d_6, r.t.) spectrum for **3i**

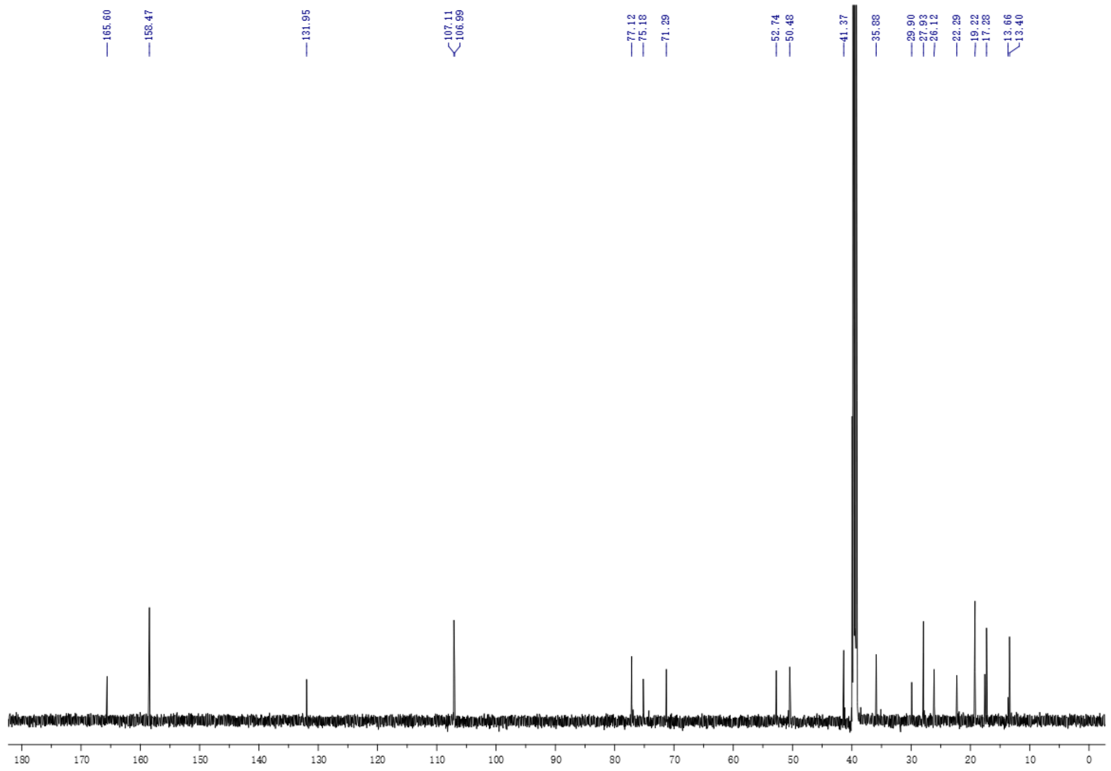

Figure S24. ^{13}C NMR (150 MHz, DMSO-d_6, r.t.) spectrum for **3i**

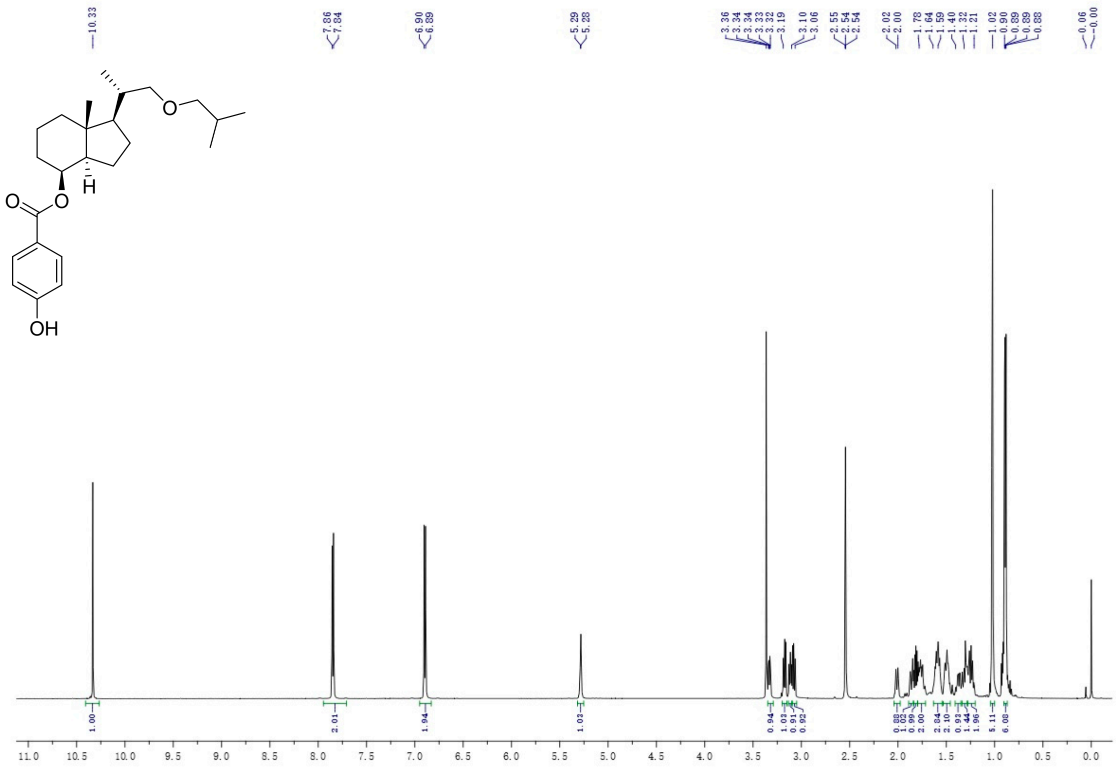

Figure S25. ^{1}H NMR (600 MHz, DMSO-d_6, r.t.) spectrum for **3j**

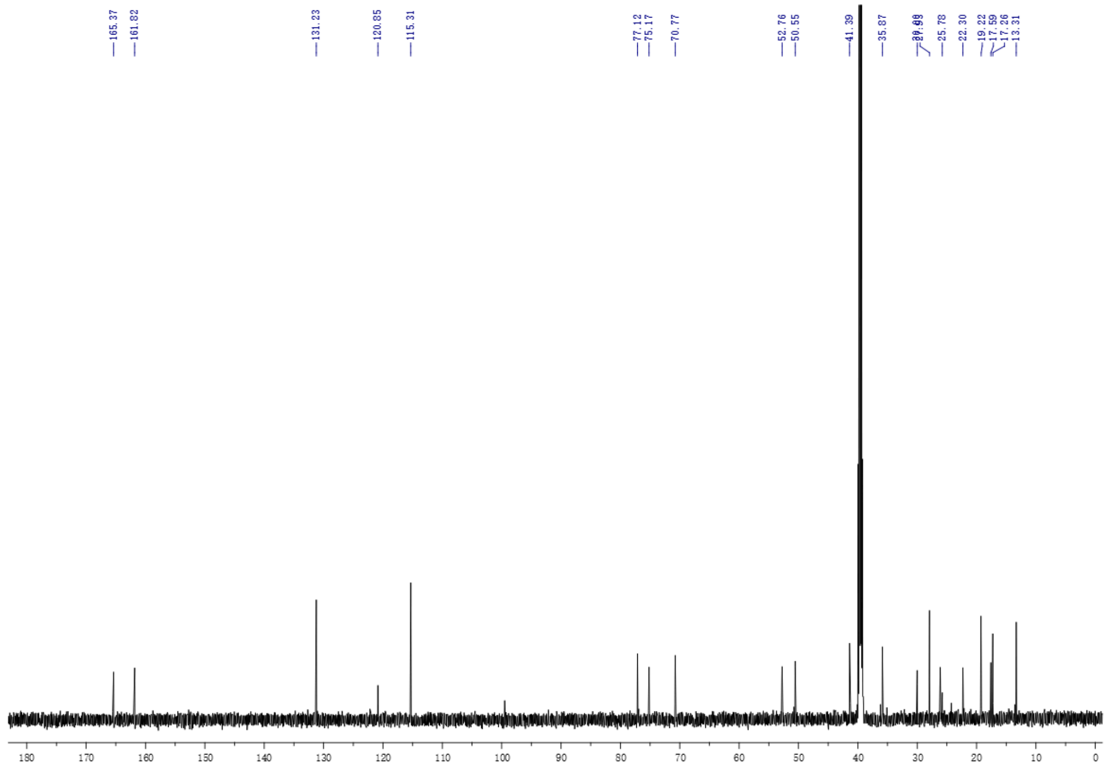

Figure S26. ^{13}C NMR (150 MHz, DMSO-d_6, r.t.) spectrum for **3j**

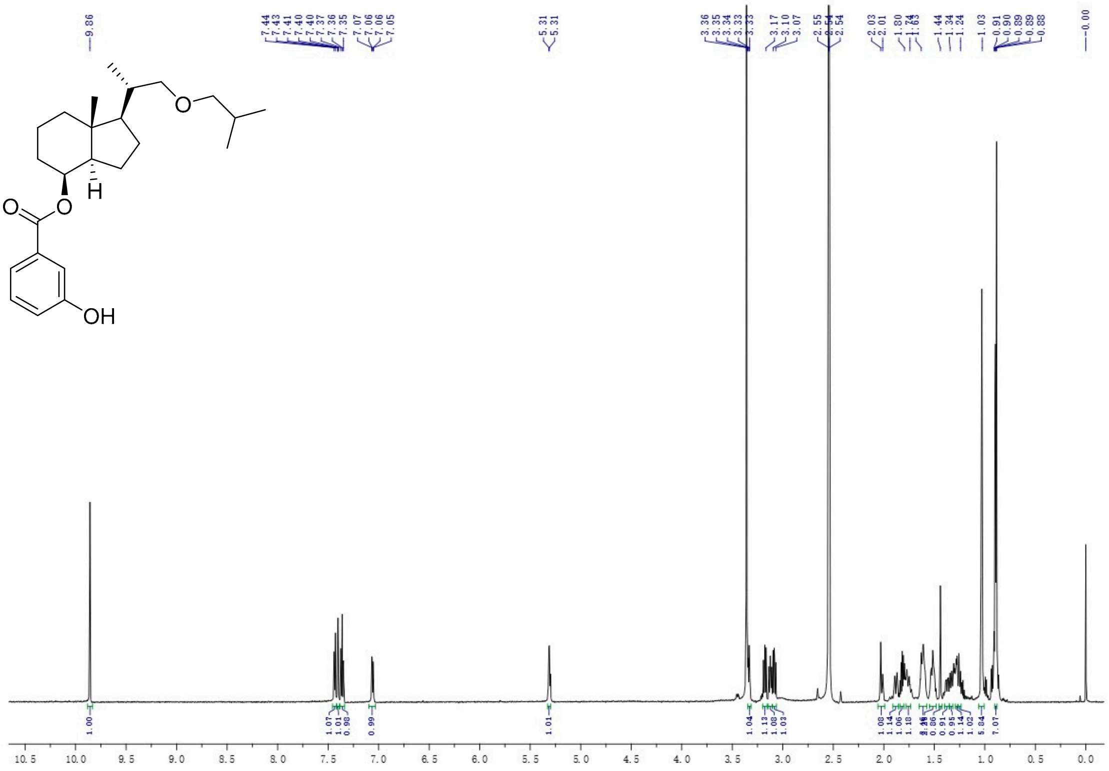

Figure S27. ^{1}H NMR (600 MHz, DMSO-*d6*, r.t.) spectrum for **3k**

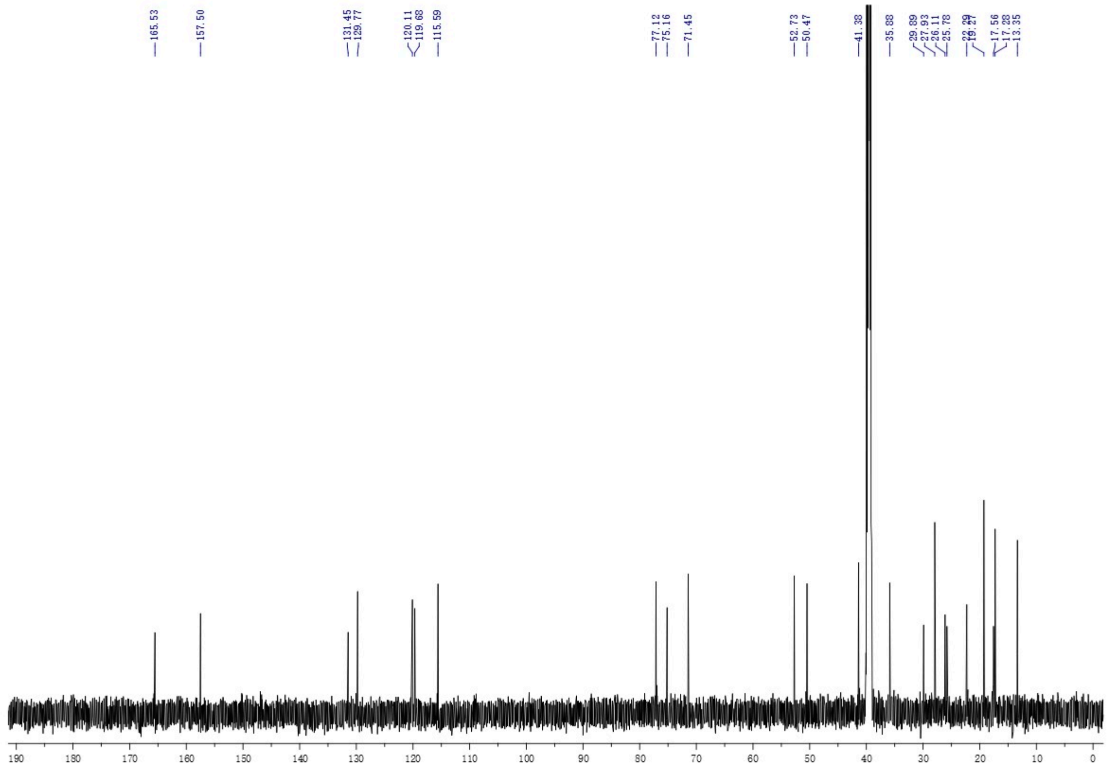

Figure S28. ^{13}C NMR (150 MHz, DMSO-*d6*, r.t.) spectrum for **3k**

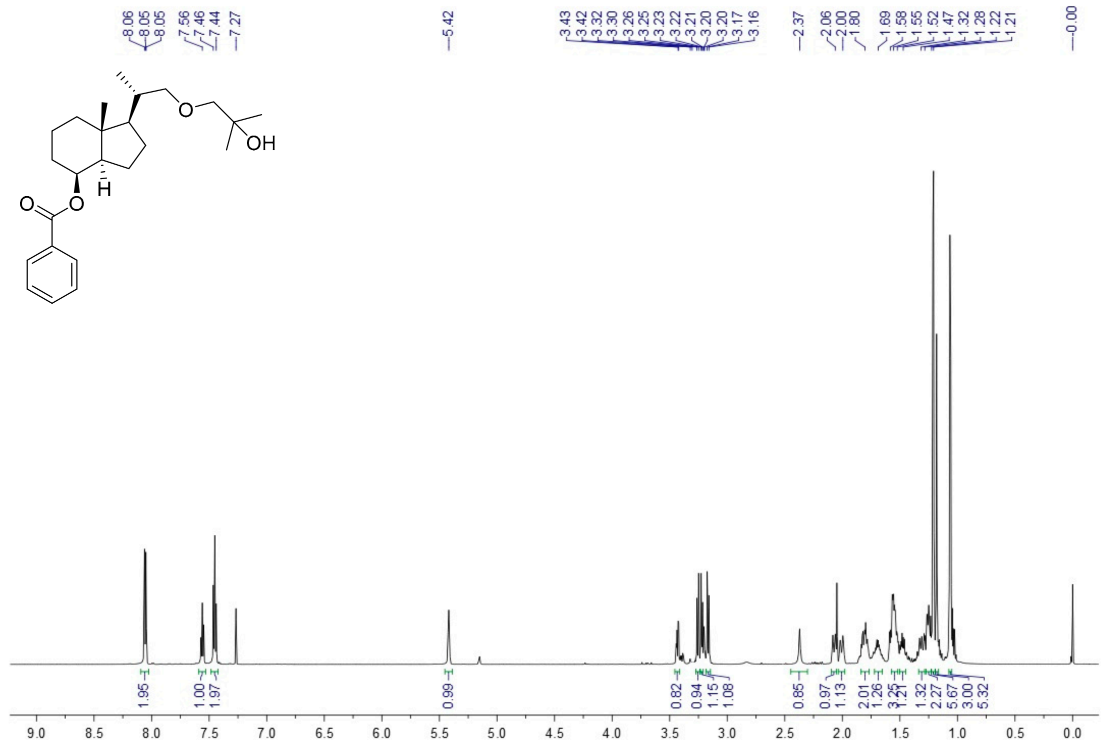

Figure S29. ^{1}H NMR (600 MHz, $CDCl_3$, r.t.) spectrum for **4a**

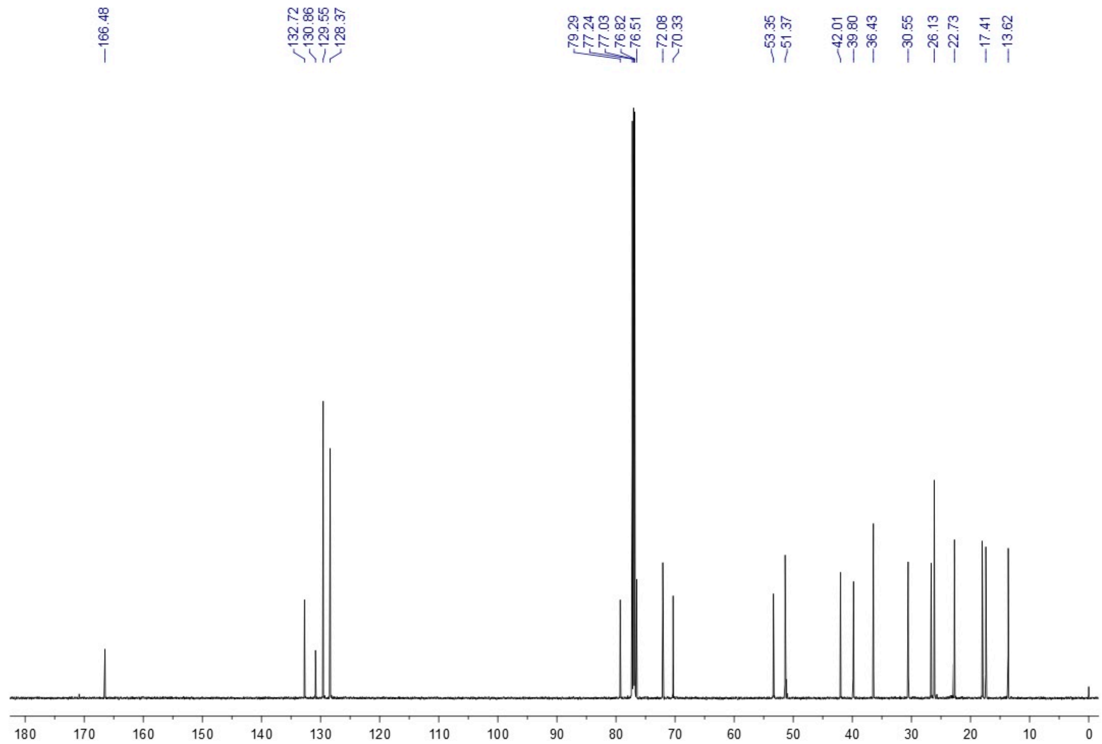

Figure S30. ^{13}C NMR (150 MHz, $CDCl_3$, r.t.) spectrum for **4a**

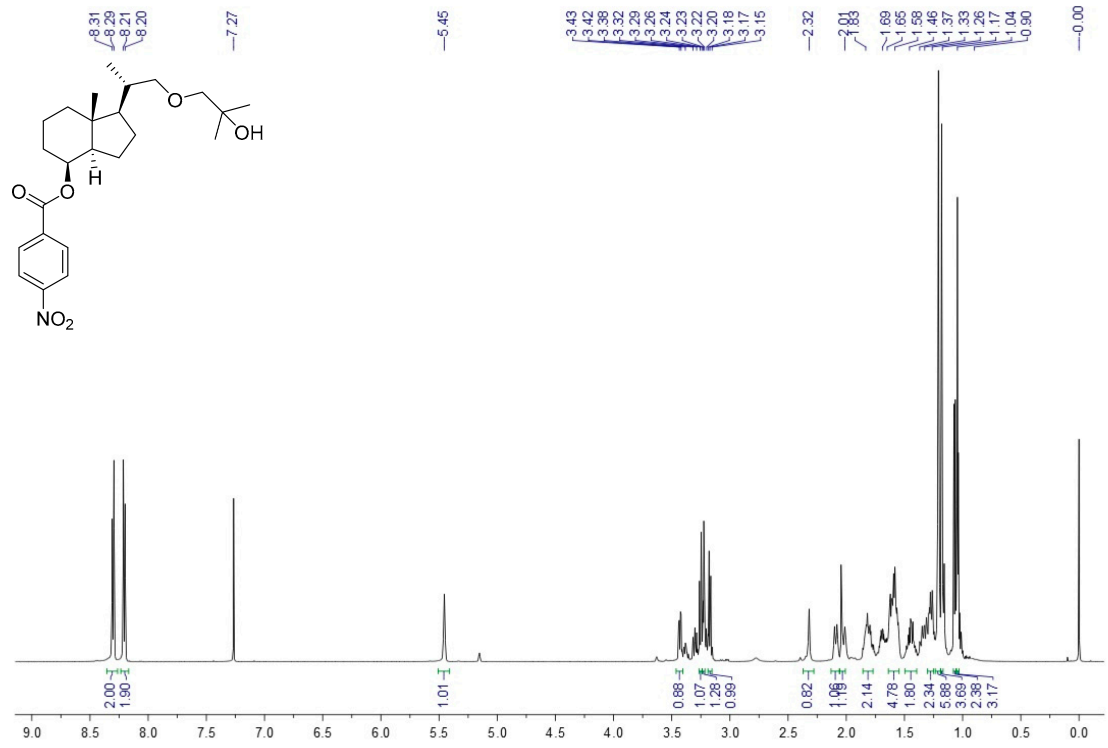

Figure S31. ^{1}H NMR (600 MHz, CDCl$_3$, r.t.) spectrum for **4b**

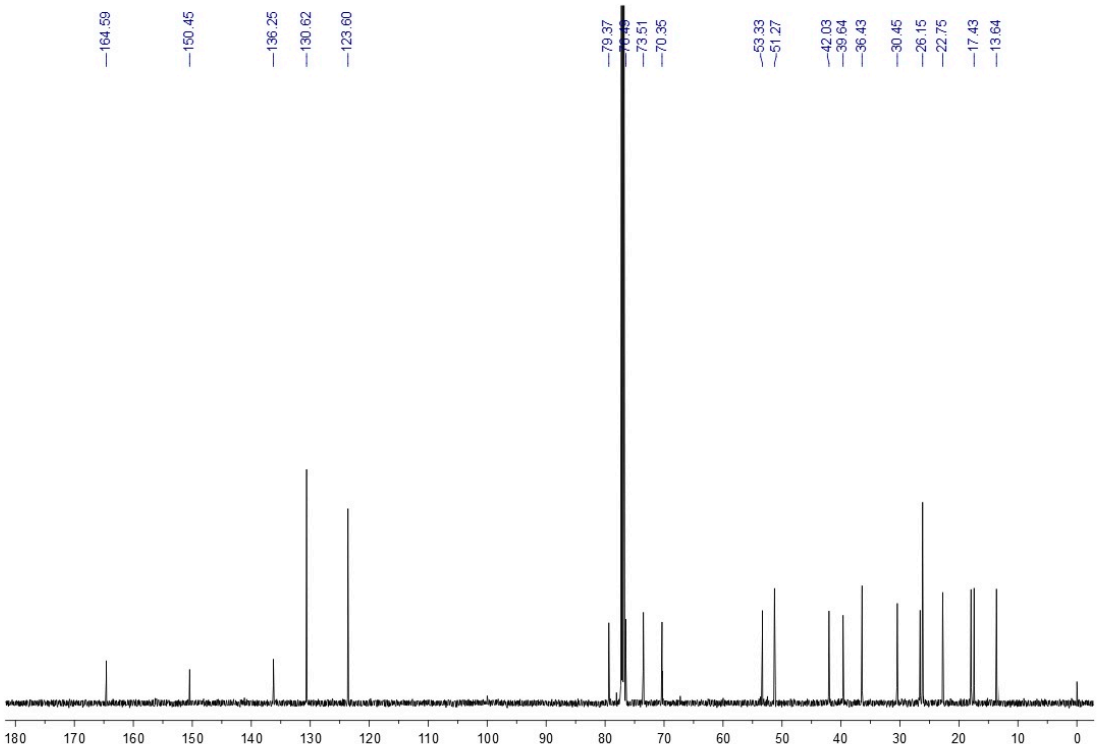

Figure S32. ^{13}C NMR (150 MHz, CDCl$_3$, r.t.) spectrum for **4b**

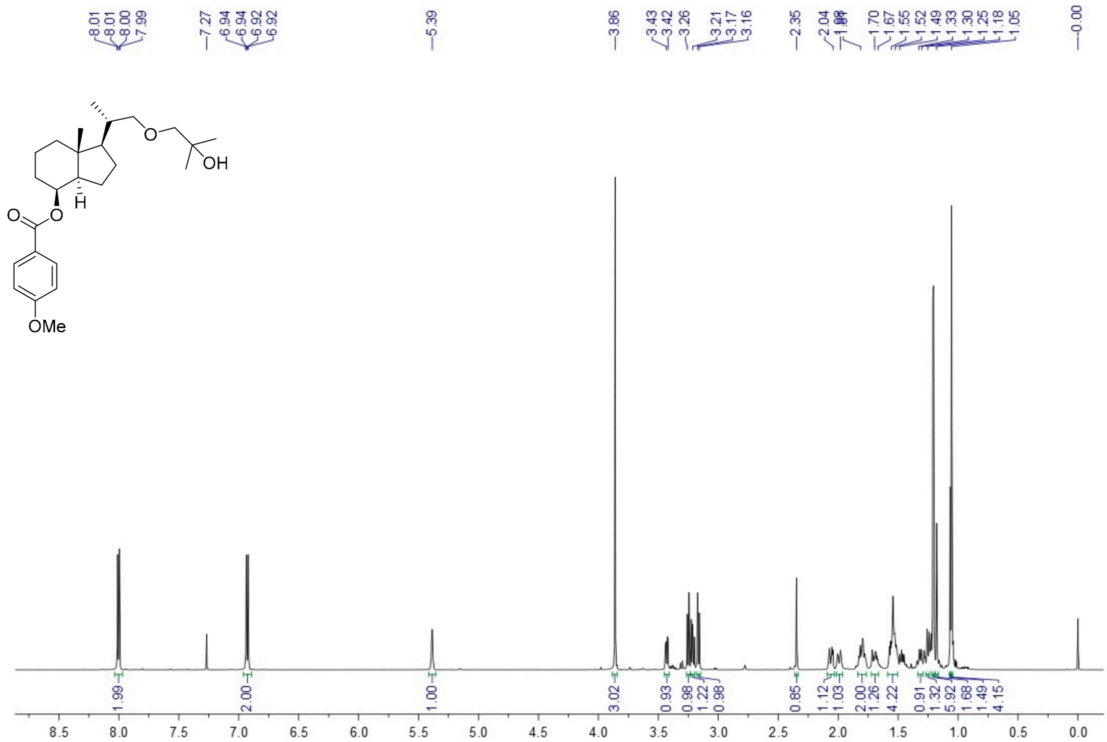

Figure S33. ^{1}H NMR (600 MHz, $CDCl_3$, r.t.) spectrum for **4c**

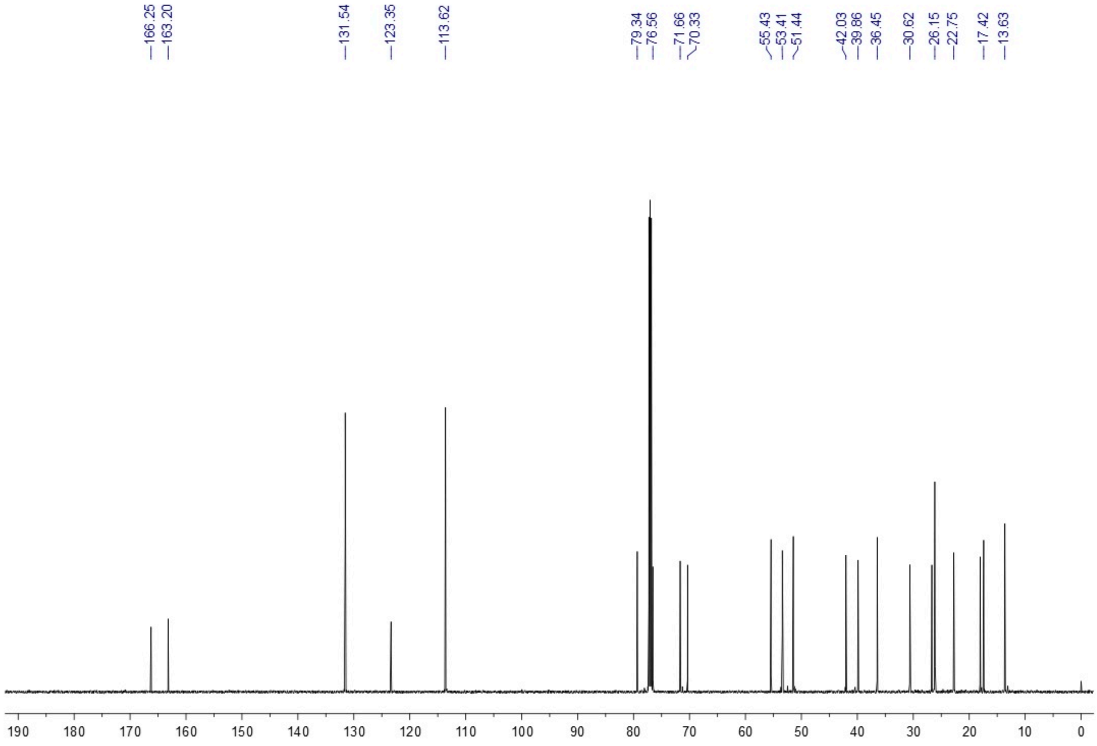

Figure S34. ^{13}C NMR (150 MHz, $CDCl_3$, r.t.) spectrum for **4c**

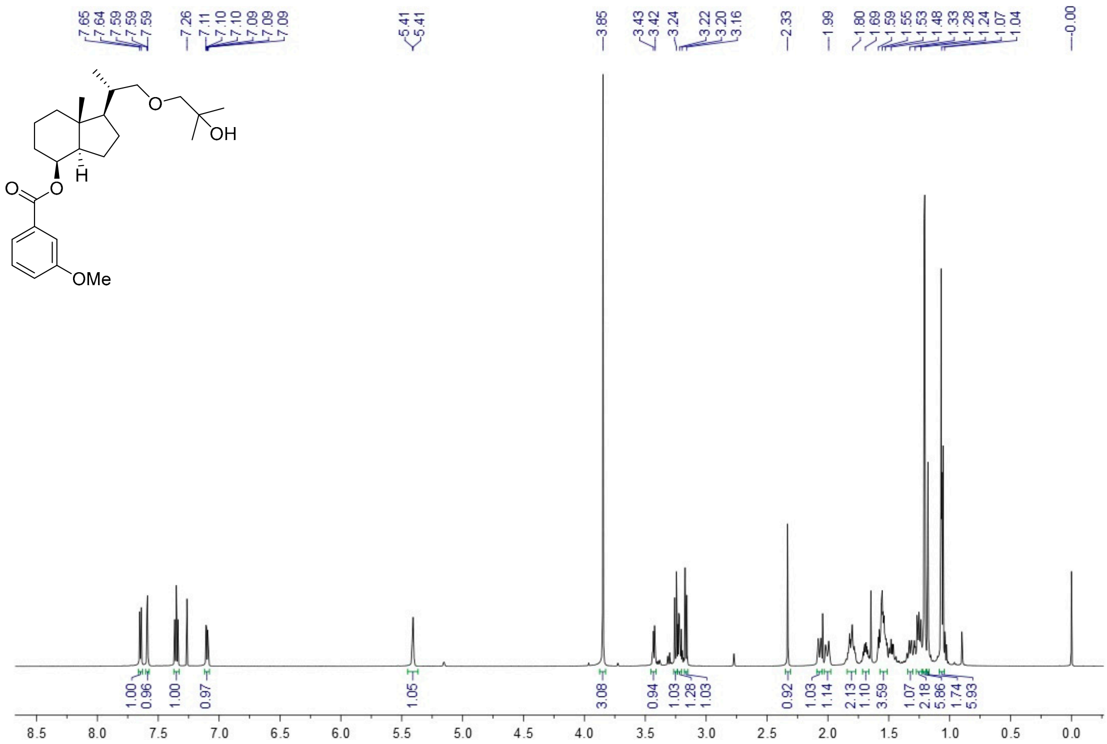

Figure S35. ^{1}H NMR (600 MHz, $CDCl_3$, r.t.) spectrum for **4d**

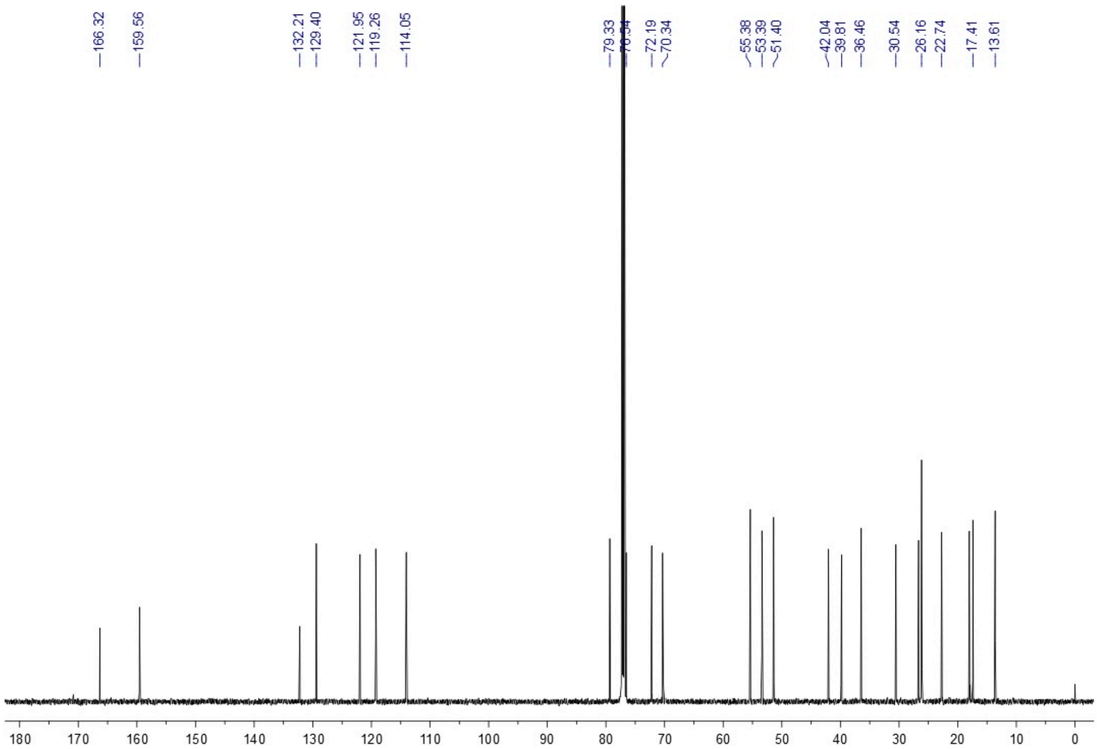

Figure S36. ^{13}C NMR (150 MHz, $CDCl_3$, r.t.) spectrum for **4d**

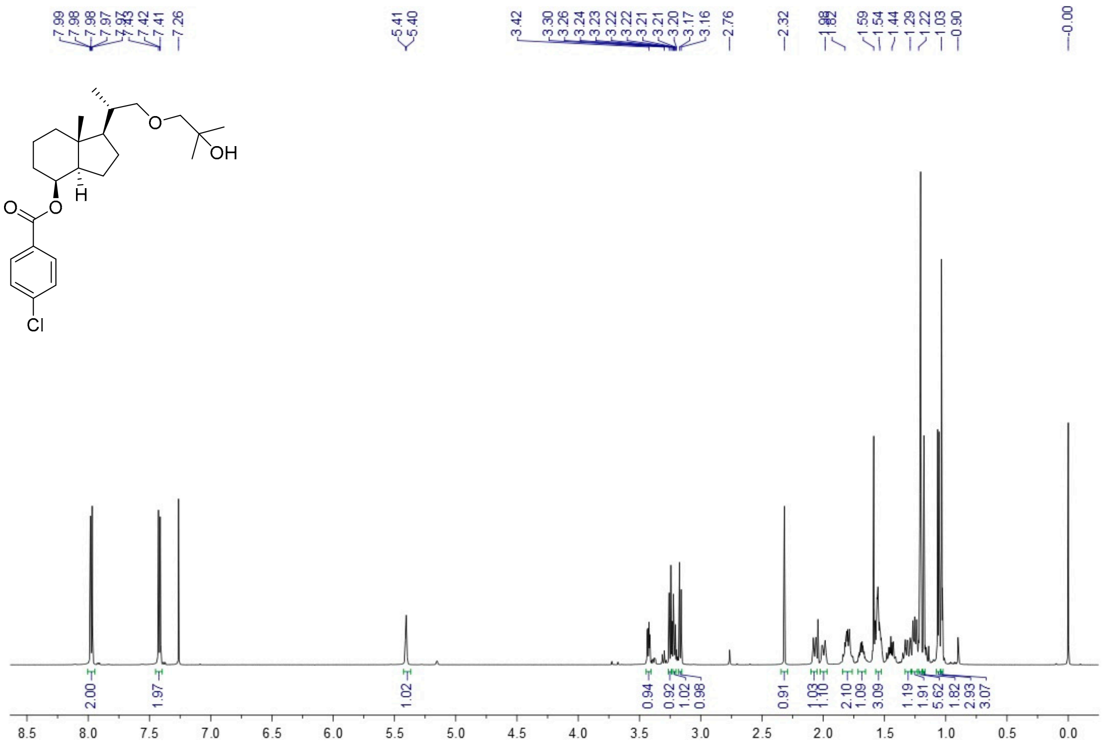

Figure S37. ^{1}H NMR (600 MHz, $CDCl_3$, r.t.) spectrum for **4e**

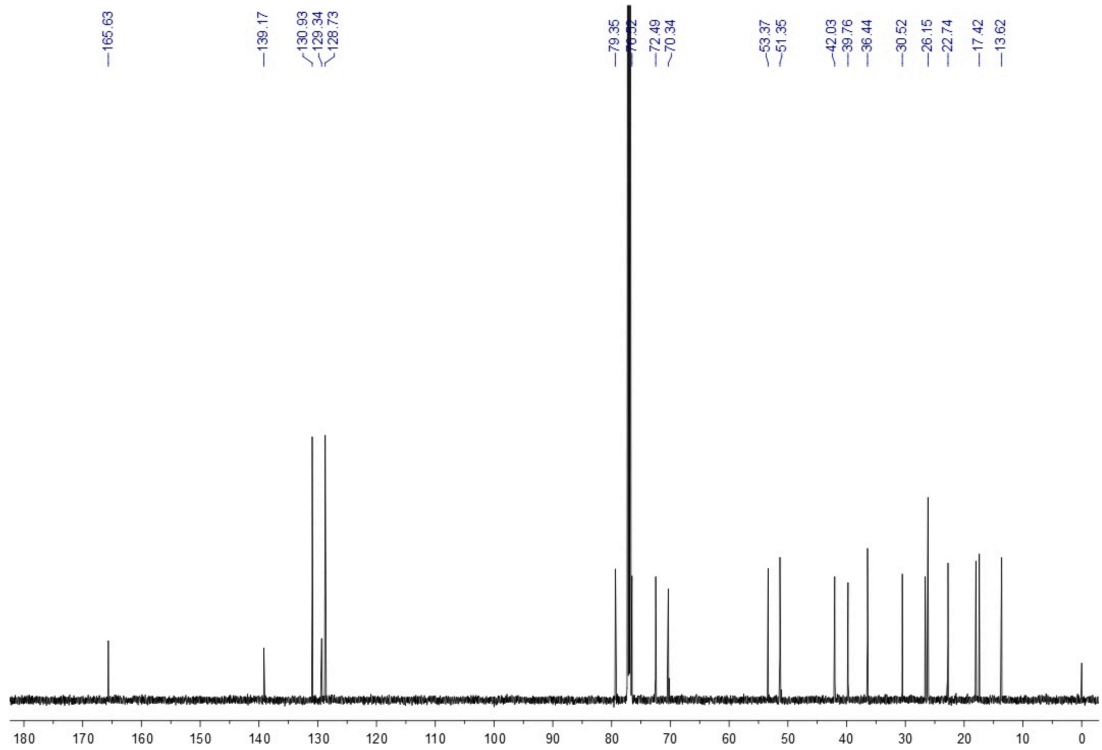

Figure S38. ^{13}C NMR (150 MHz, $CDCl_3$, r.t.) spectrum for **4e**

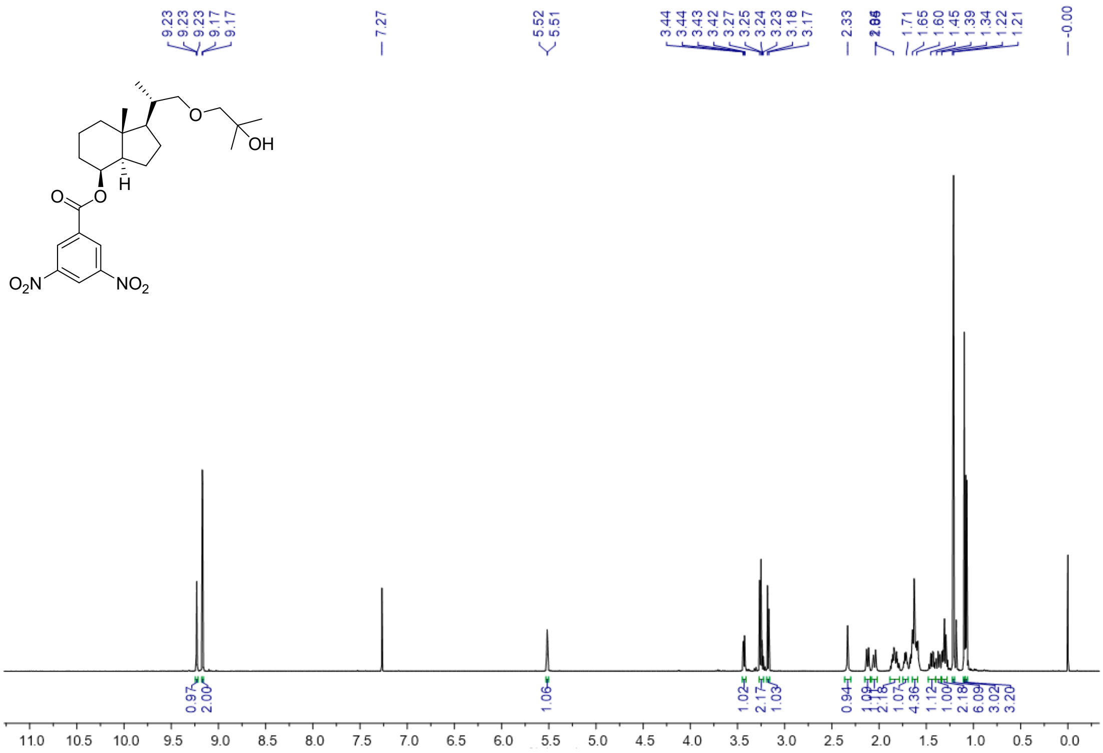

Figure S39. ^{1}H NMR (600 MHz, $CDCl_3$, r.t.) spectrum for **4f**

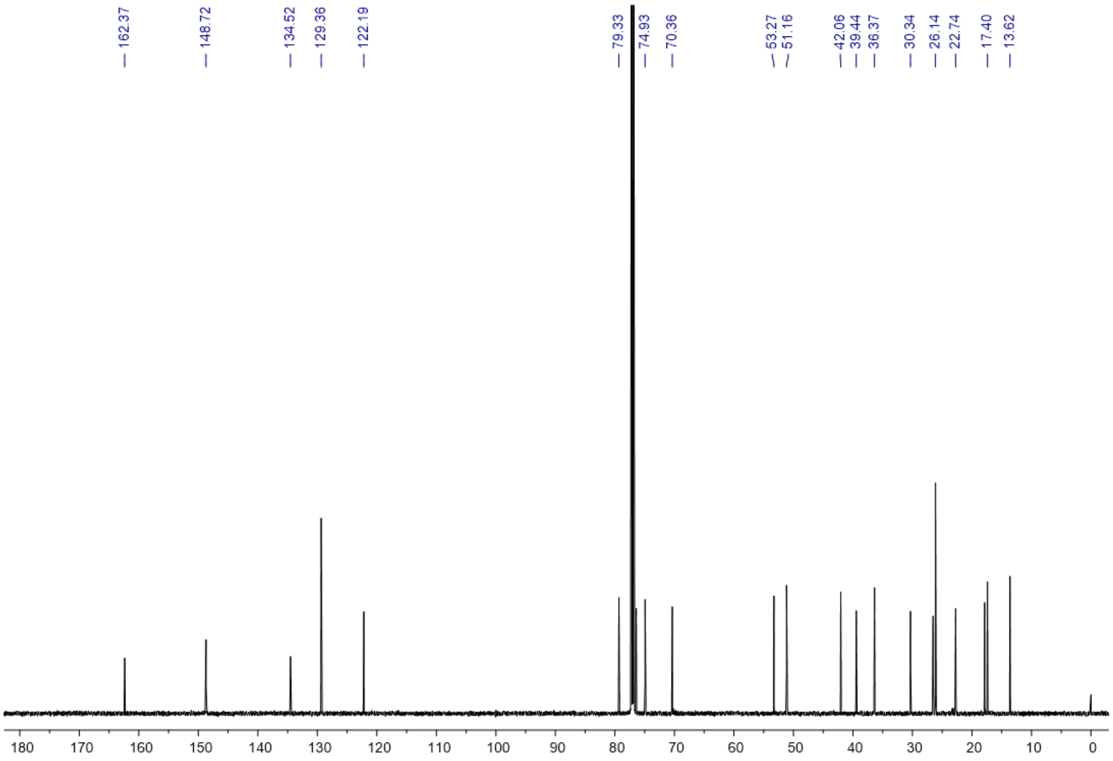

Figure S40. ^{13}C NMR (150 MHz, $CDCl_3$, r.t.) spectrum for **4f**

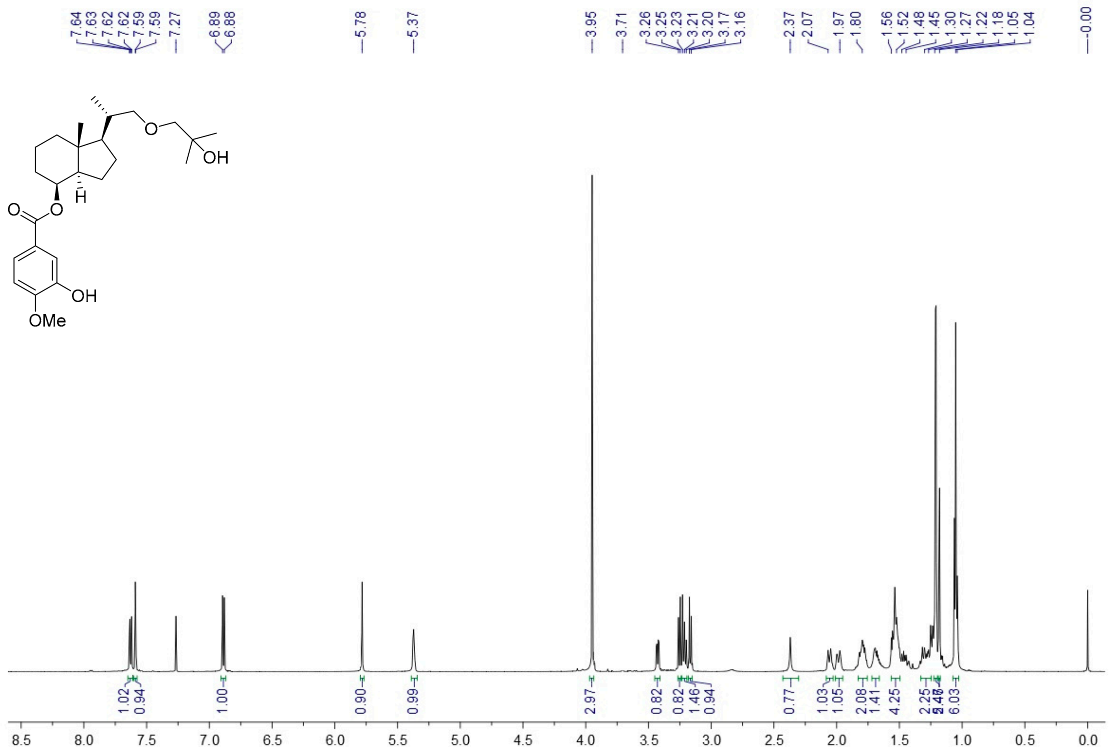

Figure S41. ^{1}H NMR (600 MHz, $CDCl_3$, r.t.) spectrum for **4g**

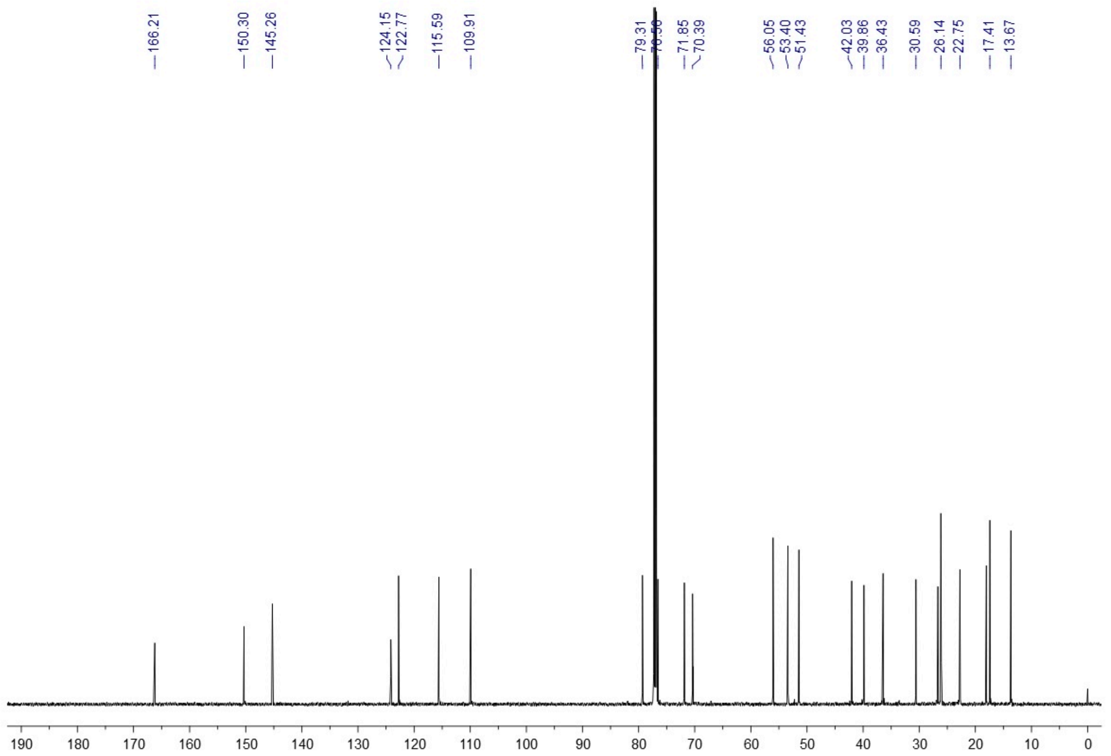

Figure S42. ^{13}C NMR (150 MHz, $CDCl_3$, r.t.) spectrum for **4g**

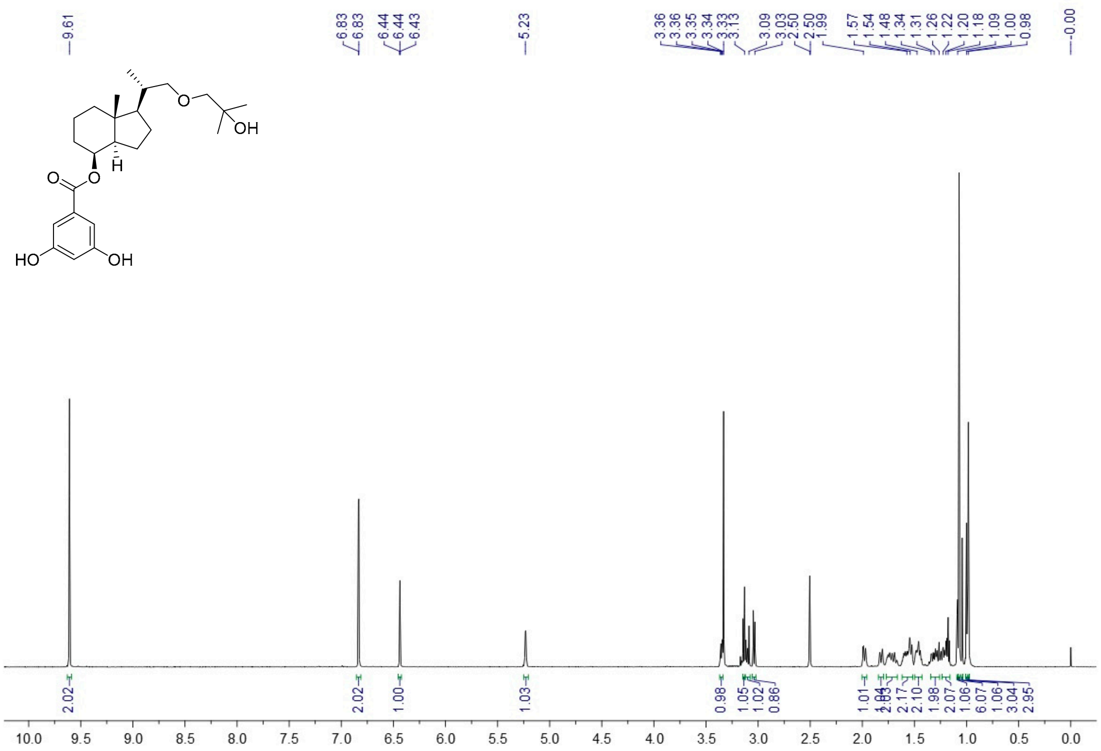

Figure S43. ^{1}H NMR (600 MHz, DMSO-d_6, r.t.) spectrum for **4h**

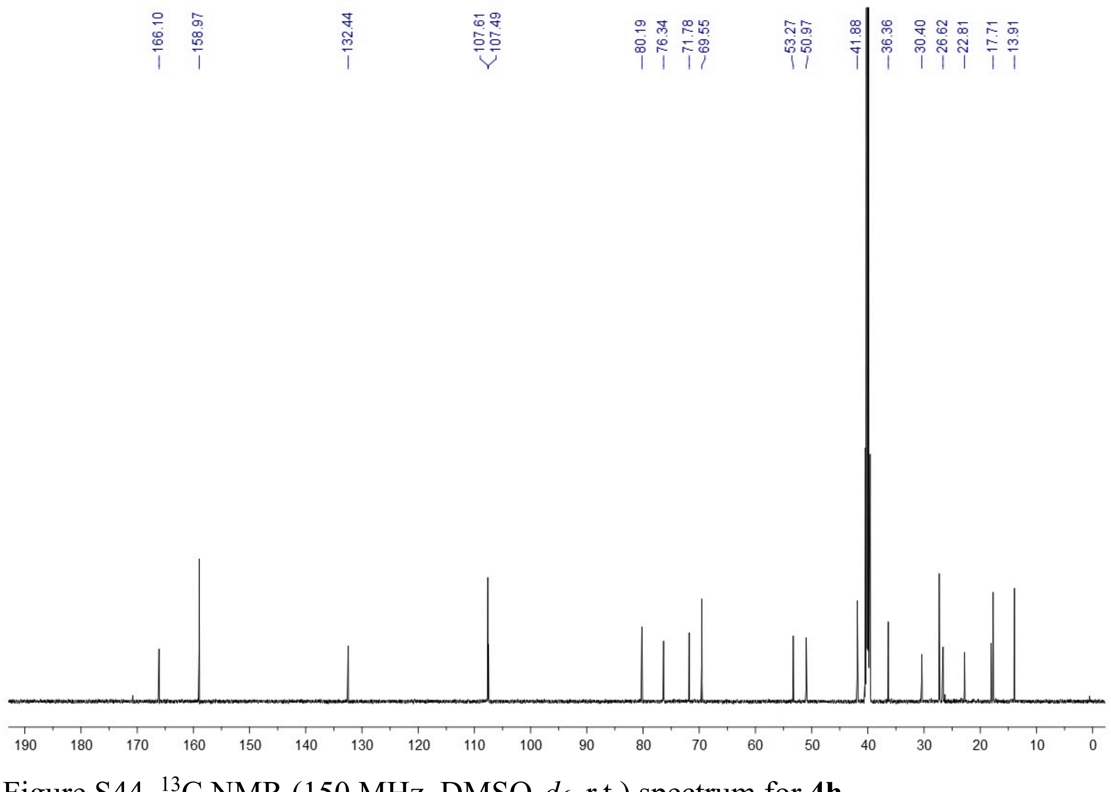

Figure S44. ^{13}C NMR (150 MHz, DMSO-d_6, r.t.) spectrum for **4h**

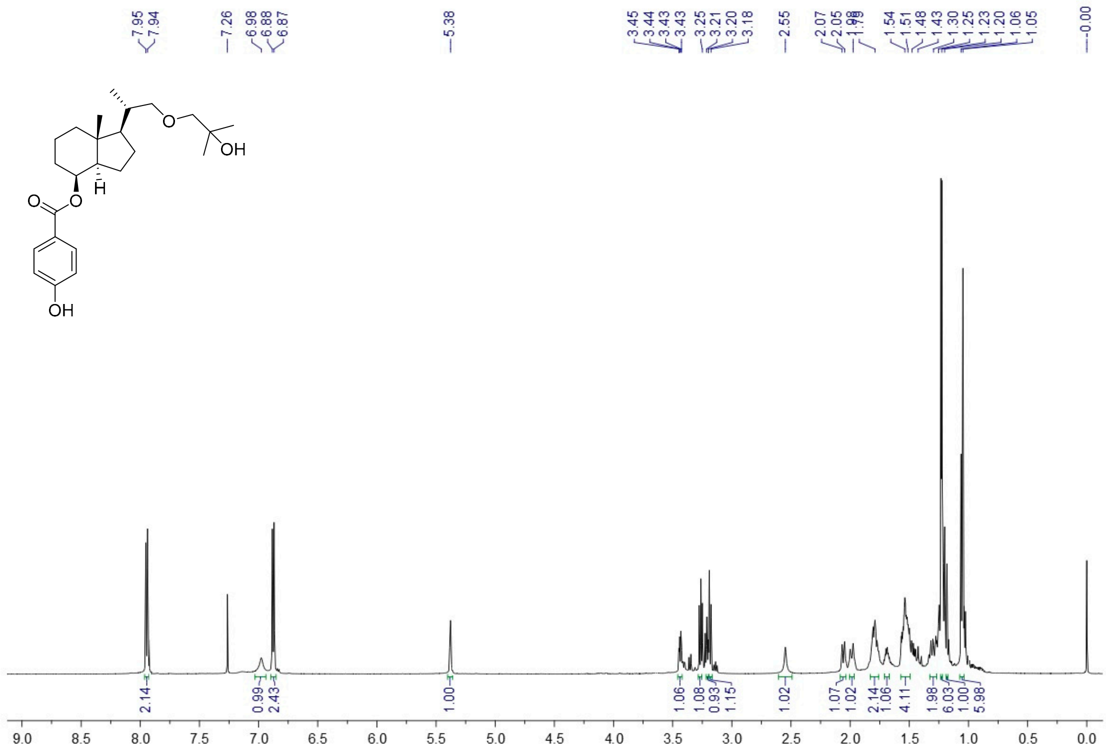

Figure S45. ^{1}H NMR (600 MHz, $CDCl_3$, r.t.) spectrum for **4i**

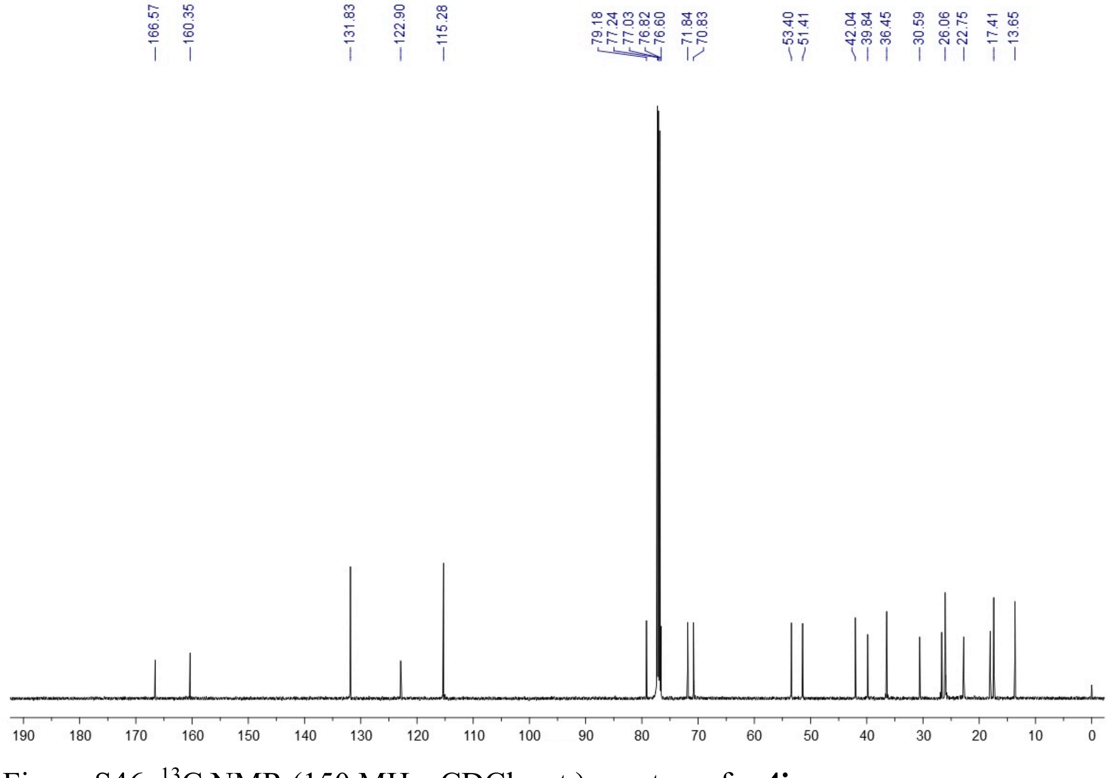

Figure S46. ^{13}C NMR (150 MHz, $CDCl_3$, r.t.) spectrum for **4i**

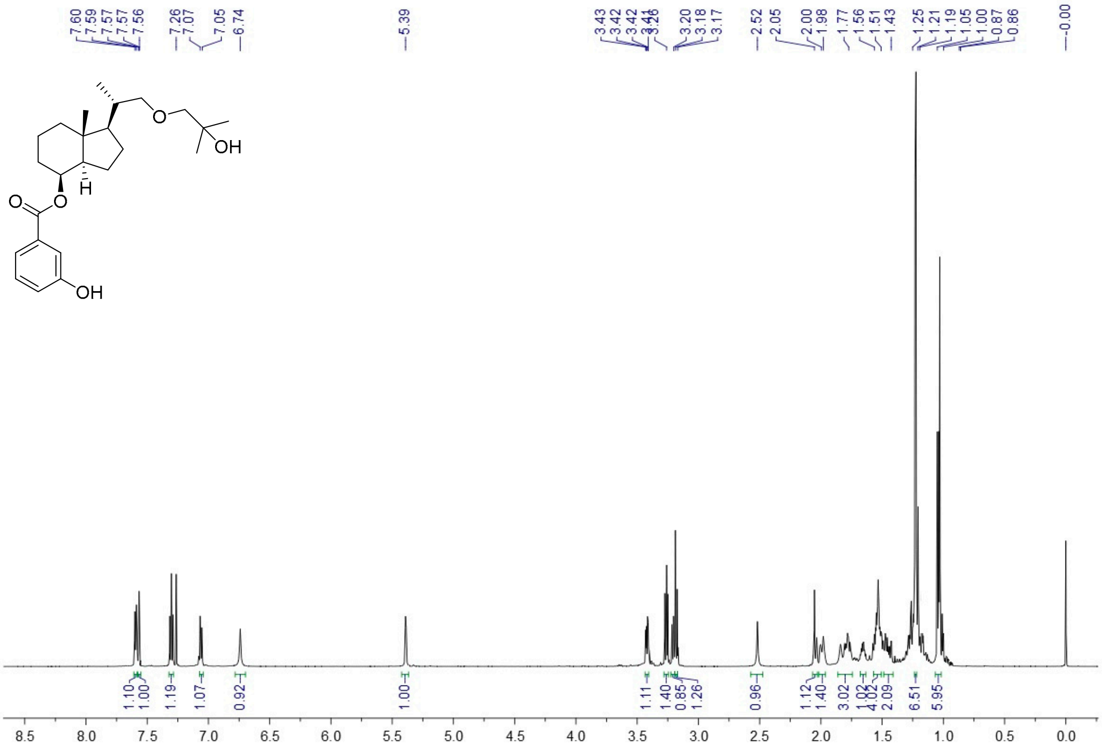

Figure S47. ^{1}H NMR (600 MHz, $CDCl_3$, r.t.) spectrum for **4j**

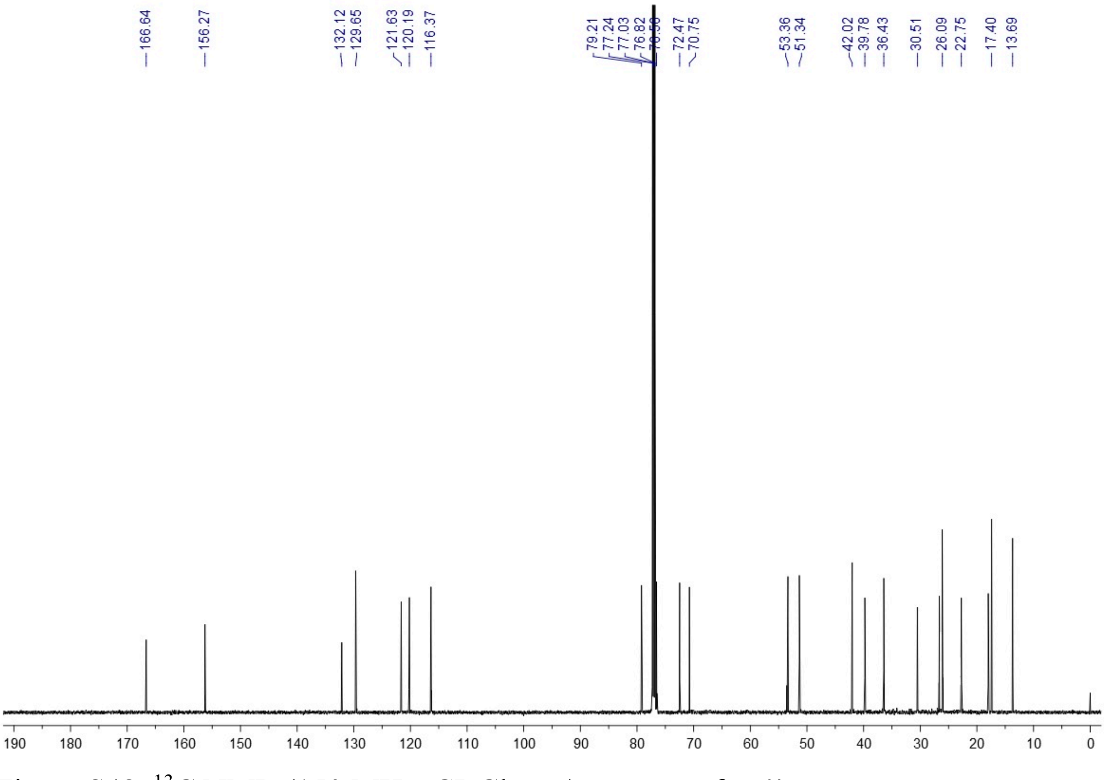

Figure S48. ^{13}C NMR (150 MHz, $CDCl_3$, r.t.) spectrum for **4j**

3. HPLC chromatogram

The compounds were determined using an Agilent 1260 HPLC instrument equipped with a G1311B 1260 Quat Pump, G7129B 1290 Vial sampler, G1316A 1260 column compartment, and G1315D 1260 DAD VL. The data and chromatograms were collected for processing using Agilent OpenLab Control Panel Software (Rev. 1.6.0.655). All sample solutions were analysed on a ZORBAX TC-C18 column (550 mm × 4.6 mm, 5 μm). The column temperature was kept at 30℃ throughout analysis, and the mobile phases consisted of MeOH (A) and water (B). Elution was performed at a flow rate of 1.0 mL/min, and the following A (60 %) and B (40 %).

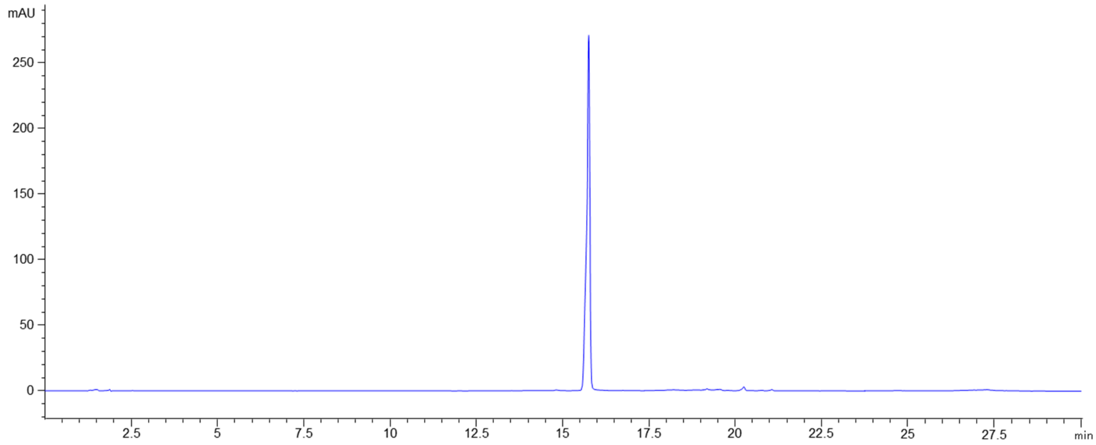

Figure S49. HPLC chromatogram of **3h**

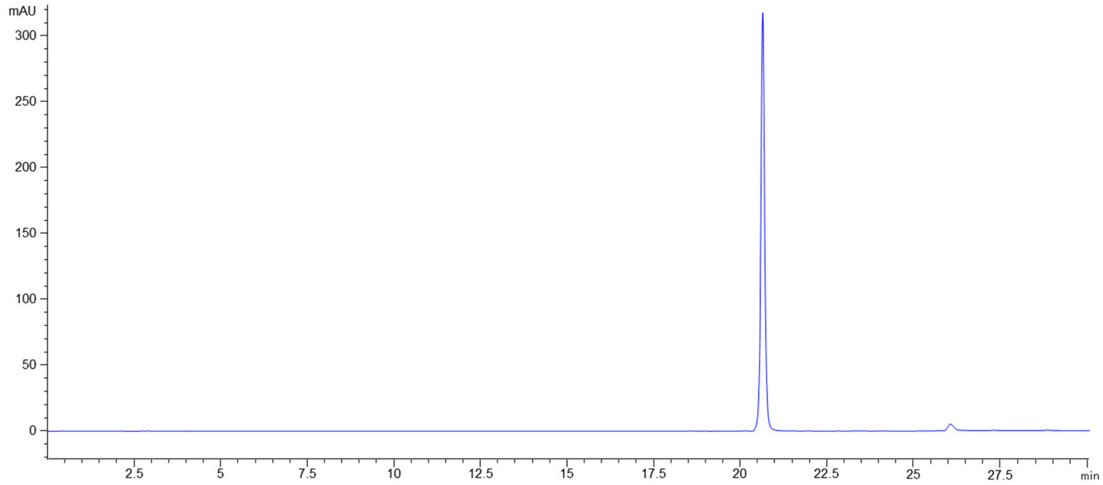

Figure S50. HPLC chromatogram of **3i**

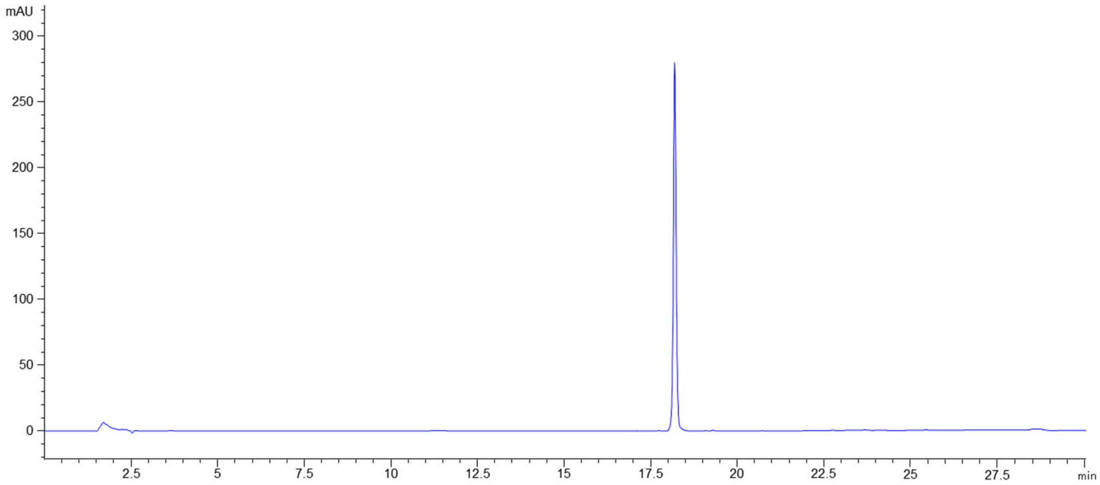

Figure S51. HPLC chromatogram of **3j**

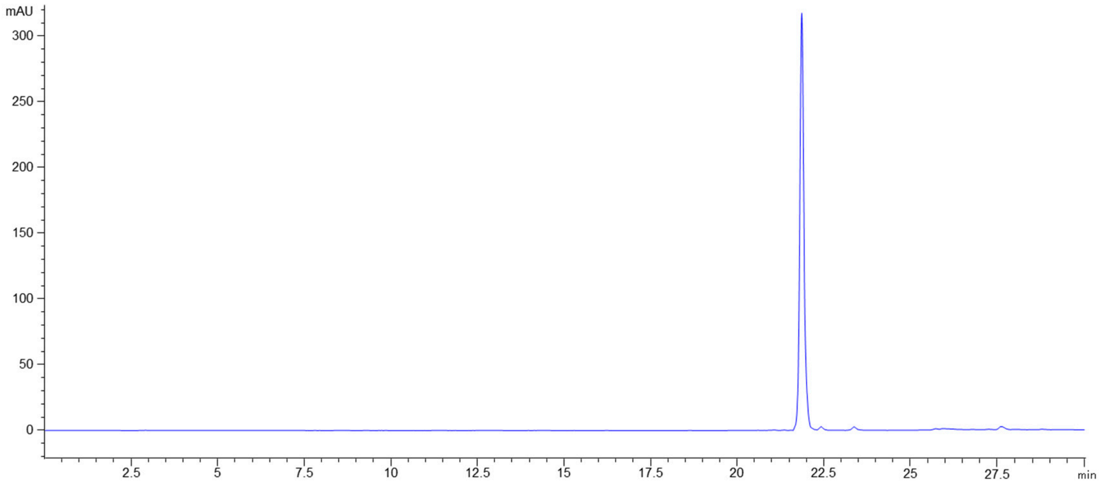

Figure S52. HPLC chromatogram of **3k**

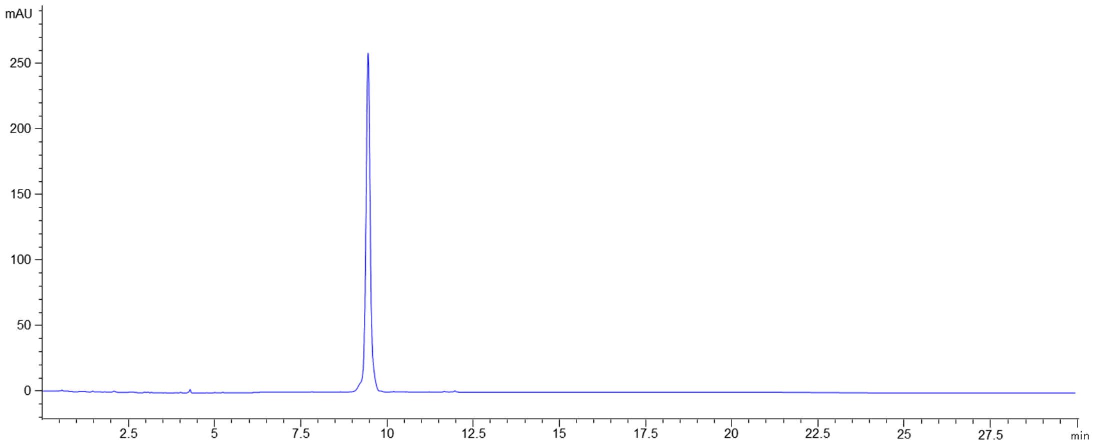

Figure S53. HPLC chromatogram of **4g**

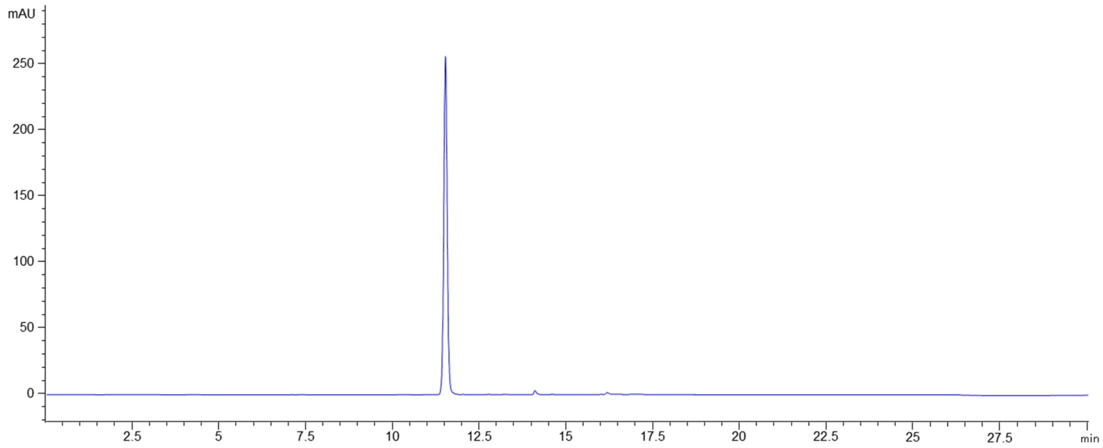

Figure S54. HPLC chromatogram of **4h**

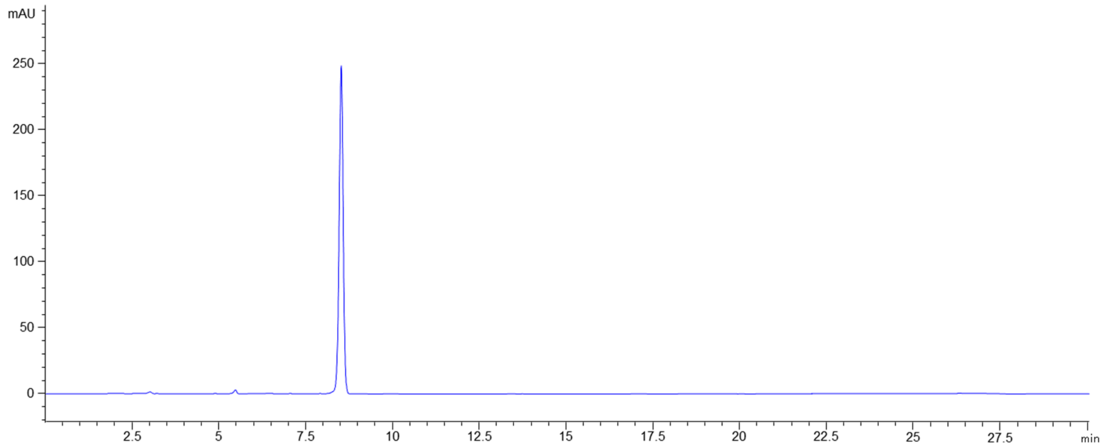

Figure S55. HPLC chromatogram of **4i**

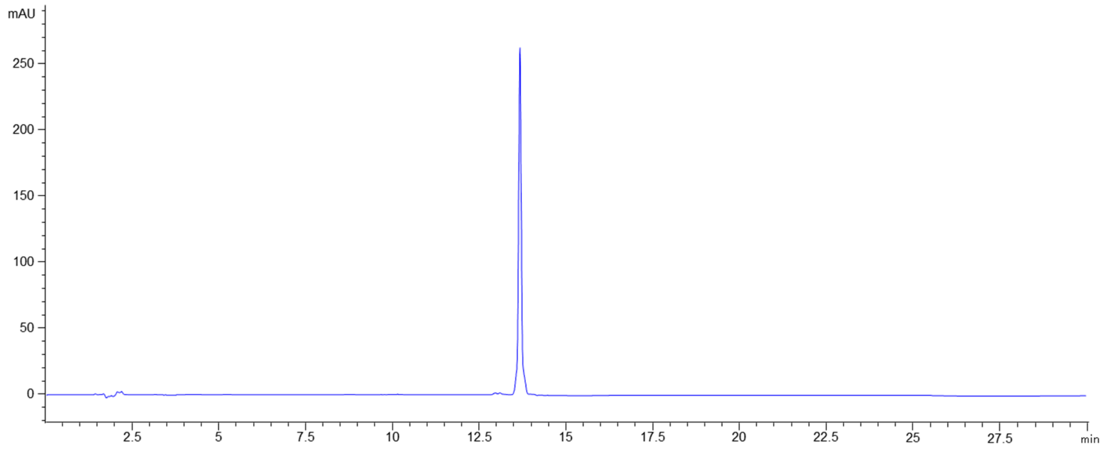

Figure S56. HPLC chromatogram of **4j**
